# Machine learning to optimize use of natriuretic peptides in the diagnosis of acute heart failure

**DOI:** 10.1093/ehjacc/zuaf051

**Published:** 2025-04-12

**Authors:** Dimitrios Doudesis, Kuan Ken Lee, Mohamed Anwar, Adam J Singer, Judd E Hollander, Camille Chenevier-Gobeaux, Yann-Erick Claessens, Desiree Wussler, Dominic Weil, Nikola Kozhuharov, Ivo Strebel, Zaid Sabti, Christopher deFilippi, Stephen Seliger, Evandro Tinoco Mesquita, Jan C Wiemer, Martin Möckel, Joel Coste, Patrick Jourdain, Komukai Kimiaki, Michihiro Yoshimura, Irwani Ibrahim, Shirley Beng Suat Ooi, Win Sen Kuan, Alfons Gegenhuber, Thomas Mueller, Olivier Hanon, Jean-Sébastien Vidal, Peter Cameron, Louisa Lam, Ben Freedman, Tommy Chung, Sean P Collins, Christopher J Lindsell, David E Newby, Alan G Japp, Anoop S V Shah, Humberto Villacorta, A Mark Richards, John J V McMurray, Christian Mueller, James L Januzzi, Nicholas L Mills, the CoDE-HF investigators, Gordon Moe, Carlos Fernando, Hanna K Gaggin, Antoni Bayes-Genis, Roland RJ van Kimmenade, Yigal Pinto, Joost HW Rutten, Anton H van den Meiracker, Luna Gargani, Nicola R Pugliese, Christopher Pemberton, Michael Neumaier, Michael Behnes, Ibrahim Akin, Michele Bombelli, Guido Grassi, Peiman Nazerian, Giovanni Albano, Philipp Bahrmann

**Affiliations:** British Heart Foundation (BHF) Centre for Cardiovascular Science, University of Edinburgh, Edinburgh EH16 4SA, UK; Usher Institute, University of Edinburgh, Edinburgh EH16 4UX, UK; British Heart Foundation (BHF) Centre for Cardiovascular Science, University of Edinburgh, Edinburgh EH16 4SA, UK; British Heart Foundation (BHF) Centre for Cardiovascular Science, University of Edinburgh, Edinburgh EH16 4SA, UK; Department of Emergency Medicine, Renaissance School of Medicine at Stony Brook University, Stony Brook, USA; Department of Emergency Medicine, Sidney Kimmel Medical College of Thomas Jefferson University, Philadelphia, USA; Department of Biochemistry, Cochin Hospital, Assistance Publique-Hopitaux de Paris, Paris, France; Department of Emergency Medicine, Princess Grace Hospital Center, Monaco, Principalty of Monaco; Department of Cardiology, Cardiovascular Research Institute of Basel, University Hospital Basel, Basel, Switzerland; Department of Internal Medicine, University Hospital Basel, University of Basel, Basel, Switzerland; Department of Cardiology, Cardiovascular Research Institute of Basel, University Hospital Basel, Basel, Switzerland; Department of Cardiology, Cardiovascular Research Institute of Basel, University Hospital Basel, Basel, Switzerland; Liverpool Heart and Chest Hospital, Liverpool L14 3PE, UK; Department of Cardiology, Cardiovascular Research Institute of Basel, University Hospital Basel, Basel, Switzerland; Department of Cardiology, Cardiovascular Research Institute of Basel, University Hospital Basel, Basel, Switzerland; Division of Cardiology, University of Maryland School of Medicine, Baltimore, USA; Division of Nephrology, University of Maryland School of Medicine, Baltimore, USA; Cardiology Division, Fluminense Federal University, Niteroi, Rio de Janeiro State, Brazil; B·R·A·H·M·S, Thermo Fisher Scientific, Hennigsdorf, Germany; Department of Emergency and Acute Medicine with Chest Pain Units, Charité—Universitätsmedizin Berlin, Campus Mitte and Virchow, Berlin, Germany; Biostatistics and Epidemiology Unit, Cochin Hospital, Paris, France; Cardiology Department, AP-HP, Paris-Saclay University, Paris, France; Division of Cardiology, The Jikei University Kashiwa Hospital, Kashiwa, Japan; Division of Cardiology, The Jikei University Kashiwa Hospital, Kashiwa, Japan; Emergency Medicine Department, National University Hospital, Singapore, Singapore; Emergency Medicine Department, National University Hospital, Singapore, Singapore; Emergency Medicine Department, National University Hospital, Singapore, Singapore; Department of Internal Medicine, Krankenhaus Bad Ischl, Bad Ischl, Austria; Department of Laboratory Medicine, Hospital Voecklabruck, Voecklabruck, Austria; Department of Geriatrics, Broca Hospital, Assistance Publique-Hôpitaux de Paris, Paris, France; Department of Geriatrics, Broca Hospital, Assistance Publique-Hôpitaux de Paris, Paris, France; Public Health and Preventive Medicine, Monash University, Melbourne, Australia; Public Health and Preventive Medicine, Monash University, Melbourne, Australia; Faculty of Health Sciences, Australian Catholic University, Melbourne, Australia; Heart Research Institute, University of Sydney, Sydney, Australia; Department of Cardiology, Concord Repatriation General Hospital, Sydney, Australia; Department of Emergency Medicine, Vanderbilt University Medical Center, and Veterans Affairs Tennessee Valley Healthcare System, Geriatric Research, Education and Clinical Center (GRECC), Nashville, TN, USA; Department of Biostatistics and Bioinformatics, Duke University, Durham, NC, USA; British Heart Foundation (BHF) Centre for Cardiovascular Science, University of Edinburgh, Edinburgh EH16 4SA, UK; British Heart Foundation (BHF) Centre for Cardiovascular Science, University of Edinburgh, Edinburgh EH16 4SA, UK; British Heart Foundation (BHF) Centre for Cardiovascular Science, University of Edinburgh, Edinburgh EH16 4SA, UK; Department of Non Communicable Disease Epidemiology, London School of Hygiene and Tropical Medicine, London, UK; Cardiology Division, Fluminense Federal University, Niteroi, Rio de Janeiro State, Brazil; Christchurch Heart Institute, University of Otago, Christchurch, New Zealand; Cardiovascular Research Institute, National University Heart Centre Singapore, Singapore; BHF Cardiovascular Research Centre, University of Glasgow, Glasgow, UK; Department of Cardiology, Cardiovascular Research Institute of Basel, University Hospital Basel, Basel, Switzerland; Division of Cardiology, Massachusetts General Hospital, Harvard Medical School, Bain Institute for Clinical Research, Boston, MA, USA; Heart Failure and Biomarker Trials, Baim Institute for Clinical Research, Boston, MA, USA; British Heart Foundation (BHF) Centre for Cardiovascular Science, University of Edinburgh, Edinburgh EH16 4SA, UK; Usher Institute, University of Edinburgh, Edinburgh EH16 4UX, UK

**Keywords:** Machine learning, Heart failure, Natriuretic peptide

## Abstract

**Aims:**

B-type natriuretic peptide (BNP) and mid-regional pro-atrial natriuretic peptide (MR-proANP) testing are guideline-recommended to aid in the diagnosis of acute heart failure. Nevertheless, the diagnostic performance of these biomarkers is uncertain.

**Methods and results:**

We performed a systematic review and individual patient-level data meta-analysis to evaluate the diagnostic performance of BNP and MR-proANP. We subsequently developed and externally validated a decision-support tool called CoDE-HF that combines natriuretic peptide concentrations with clinical variables using machine learning to report the probability of acute heart failure. Fourteen studies from 12 countries provided individual patient-level data in 8493 patients for BNP and 3899 patients for MR-proANP, in whom, 48.3% (4105/8493) and 41.3% (1611/3899) had an adjudicated diagnosis of acute heart failure, respectively. The negative predictive value (NPV) of guideline-recommended thresholds for BNP (100 pg/mL) and MR-proANP (120 pmol/L) was 93.6% (95% confidence interval 88.4–96.6%) and 95.6% (92.2–97.6%), respectively, whilst the positive predictive value (PPV) was 68.8% (62.9–74.2%) and 64.8% (56.3–72.5%). Significant heterogeneity in the performance of these thresholds was observed across important subgroups. CoDE-HF was well calibrated with excellent discrimination in those without prior acute heart failure for both BNP and MR-proANP [area under the curve of 0.914 (0.906–0.921) and 0.929 (0.919–0.939), and Brier scores of 0.110 and 0.094, respectively]. CoDE-HF with BNP and MR-proANP identified 30% and 48% as low-probability [NPV of 98.5% (97.1–99.3%) and 98.5% (97.7–99.0%)], and 30% and 28% as high-probability [PPV of 78.6% (70.4–85.0%) and 75.1% (70.9–78.9%)], respectively, and performed consistently across subgroups.

**Conclusion:**

The diagnostic performance of guideline-recommended BNP and MR-proANP thresholds for acute heart failure varied significantly across patient subgroups. A decision-support tool that combines natriuretic peptides and clinical variables was more accurate and supports more individualized diagnosis.

**Study registration:**

PROSPERO number, CRD42019159407.

## Graphical Abstract

**Graphical Abstract:**
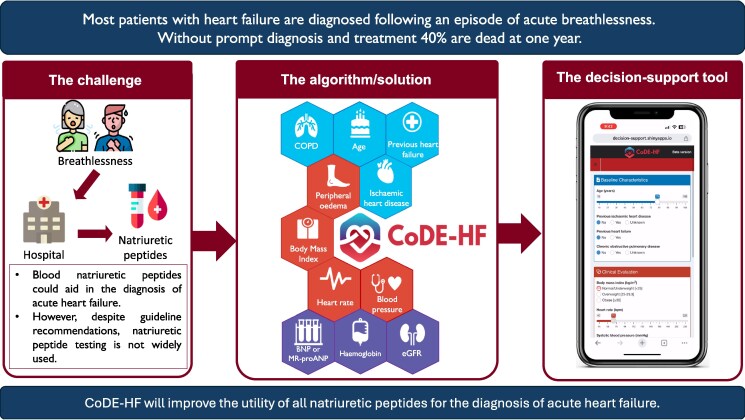

## Introduction

Suspected acute heart failure is one of the commonest reasons for emergency department attendance and unplanned hospital admission.^1,2^ Accurate and timely diagnosis is challenging because many other conditions can present with similar symptoms and signs. National and international guidelines recommend the use of natriuretic peptide testing with uniform thresholds to aid in the diagnosis of acute heart failure.^3–8^ However, natriuretic peptide concentrations are known to be influenced by various patient factors such as body-mass index, renal function and age, each of which may affect diagnostic performance.^9–11^ This has, in part, limited the reliability of natriuretic peptides in clinical practice.

There are currently three natriuretic peptides recommended for the diagnosis of acute heart failure—N-terminal pro-B-type natriuretic peptide (NT-proBNP), B-type natriuretic peptide (BNP) and mid-regional pro-atrial natriuretic peptide (MR-proANP).^6^ We previously demonstrated that guideline-recommended thresholds of NT-proBNP have comparatively lower accuracy in older patients, those with obesity, renal dysfunction and prior heart failure.^12^ We subsequently developed and validated a decision-support tool called CoDE-HF (Collaboration for the Diagnosis and Evaluation of Heart Failure) (https://decision-support.shinyapps.io/code-hf/) to calculate an individualized probability of acute heart failure for each patient.^12^ CoDE-HF uses machine learning to incorporate NT-proBNP concentrations as a continuous variable alongside other objective physiological and patient factors that are routinely collected during the initial clinical assessment. We demonstrated that CoDE-HF ruled-in and ruled-out acute heart failure more accurately than any approach using NT-proBNP thresholds alone. However, NT-proBNP testing is not available in all healthcare systems; whether the CoDE-HF approach could improve performance of BNP and MR-proANP is unclear. Accordingly, the aim of this study was to evaluate the diagnostic performance of current guideline-recommended BNP and MR-proANP thresholds for acute heart failure across patient subgroups and to develop and validate the CoDE-HF decision-support tool for BNP and MR-proANP.

## Methods

### Study population

A systematic review was performed to identify studies that evaluated BNP and MR-proANP in the diagnosis of acute heart failure. A previous review by Roberts et al.^3^ was updated by searching Embase, Medline and the Cochrane Central Register of Controlled Trials for studies published up to 18 August 2021 using the following keywords: ‘heart failure’ and ‘natriuretic peptide’ (see Supplementary material online, *Text S1*). Studies were included if they satisfied the following inclusion criteria: (i) enrolled patients ≥18 years with suspected acute heart failure in an acute care setting, (ii) measured BNP or MR-proANP on blood samples obtained during the initial assessment, and (iii) adjudicated the diagnosis of acute heart failure using an acceptable reference standard. A pre-specified protocol (PROSPERO register: CRD42019159407) was used by two investigators (KKL and MA) to independently screen all studies identified in the systematic literature search. and conflicts were adjudicated by a third investigator (NLM).

The corresponding authors of all eligible cohorts were contacted to request anonymized individual patient-level data on BNP and MR-proANP concentrations, adjudicated diagnosis of acute heart failure, demographics (age, sex, ethnicity), past medical history (heart failure, ischaemic heart disease, diabetes, hypertension, hyperlipidemia, smoking, asthma, chronic obstructive pulmonary disease (COPD), chronic kidney disease), physiological variables (heart rate and blood pressure), and clinical haematology and biochemistry profiles. The accuracy and completeness of the individual patient-level data were checked with all corresponding authors prior to harmonisation. All studies were conducted in accordance with the Declaration of Helsinki and with ethical approval to permit sharing of individual patient-level data to conduct this analysis.

### BNP and MR-proANP threshold analysis

A two-stage approach was used to calculate meta-estimates with 95% confidence intervals of the sensitivity, specificity, negative predictive value (NPV) and positive predictive value (PPV) of guideline-recommended BNP and MR-proANP thresholds for acute heart failure (100 pg/mL and 120 pmol/L, respectively).^4,6^ These metrics were calculated separately within each study, then pooled across studies in a binomial-normal random effects model using the method of DerSimonian and Laird.^13^ The performance of these thresholds was further evaluated in the overall population and subsequently in pre-specified subgroups that are known to influence natriuretic peptide levels and the diagnosis of acute heart failure [age, sex, ethnicity, body mass index, renal function, anaemia and the presence of comorbidities (prior heart failure, hypertension, hyperlipidemia, diabetes mellitus, atrial fibrillation, COPD)]. The diagnostic performance of BNP and MR-proANP concentrations was subsequently evaluated across various levels to establish a rule-out threshold that identifies the highest proportion of patients as low-probability with an NPV ≥98%, and a rule-in threshold that identifies the highest proportion of patients as high-probability with a PPV ≥75%.

### Model development and validation

A decision-support tool [Collaboration for the Diagnosis and Evaluation of Heart Failure (CoDE-HF)] was developed and validated using extreme gradient boosting (XGBoost)^14^ to compute a value (0–100) corresponding to an individual patient’s probability of acute heart failure. CoDE-HF was developed and validated for both BNP and MR-proANP separately.

The model was developed for individuals with and without prior heart failure separately due to differences in the demographics, comorbidities, and prevalence of acute heart failure in these two groups. BNP and MR-proANP concentrations were used as a continuous measure together with selected clinical variables associated with acute heart failure, which were found to have the highest relative importance in our model training phase [age, estimated glomerular filtration rate (eGFR), haemoglobin, body mass index, heart rate, blood pressure, peripheral oedema, prior history of heart failure, COPD and ischaemic heart disease].

Ten datasets were multiply imputed using joint-modelling multiple imputation with random study-specific covariance matrices fitted with a Markov chain Monte Carlo algorithm to account for missing data in the cohorts.^15^ Ten iterations of 10-fold cross-validation were performed for each model. The median score across the iterations and imputed datasets was used as the CoDE-HF score for each patient. High- and low-probability thresholds for CoDE-HF were pre-specified as the scores that classified the greatest proportion of patients with a rule-in performance of 75% PPV and 90% specificity, and a rule-out performance of 98% NPV and 90% sensitivity, respectively.

The performance of each model was subsequently evaluated using a range of diagnostic metrics including the area under the receiver operator curve (AUC), Brier score, proportion of patients identified as high- and low-probability, and the PPV and NPV in the overall cohort and across subgroups. Brier score is a measure of both discrimination and calibration and is calculated by taking the mean squared difference between predicted probabilities and the observed outcome. A lower Brier score indicates better model performance, with scores close to zero indicating perfect calibration and discrimination, while scores closer to one indicate poor performance.^16^

A decision curve analysis and internal-external cross-validation were performed to evaluate the performance of CoDE-HF. In brief, this approach iteratively leaves one study out at a time for external validation and uses the remaining studies for model development.^17^ Imputation was not performed in the external validation. The incidence of all-cause death was evaluated stratified by CoDE-HF into probability groups. All analyses were performed in R version 4.2.0.

### Patient and public involvement

Members of a patient and public panel were involved in the interpretation of results. There are plans to disseminate the results of the research to relevant patient communities.

## Results

### Study population

Fourteen studies from 12 countries provided individual patient-level data in 8493 patients for BNP [mean age 69 (±16) years, 46% women], and 3899 patients for MR-proANP [mean age 66 (±17) years, 42% women], in whom, 48.3% (4105/8493) and 41.3% (1611/3899) had a diagnosis of acute heart failure confirmed by adjudication, respectively (*Table 1*, Supplementary material online, *Figure S1* and *Tables S1*–S3).^18–31^ Patients with a prior history of heart failure had a higher prevalence of acute heart failure than those without (75% vs. 33% and 74% vs. 27% for BNP and MR-proANP, respectively) (see Supplementary material online, *Table S4*).

**Table 1:** Baseline characteristics of patients stratified by diagnosis of acute heart failure

	BNP			MR-proANP		
	Overall	Patients with acute heart failure	Patients without acute heart failure	Overall	Patients with acute heart failure	Patients without acute heart failure
**Number of participants**	8493	4105	4388	3899	1611	2288
**Men**	4559 (53.7)	2287 (55.7)	2272 (51.8)	2258 (57.9)	1005 (62.4)	1253 (54.8)
**Age, years**						
<50	1126 (13.3)	271 (6.6)	855 (19.6)	680 (17.4)	94 (5.8)	586 (25.6)
50–75	3639 (43.1)	1569 (38.4)	2070 (47.4)	1826 (46.8)	639 (39.7)	1187 (51.9)
>75	3687 (43.6)	2244 (54.9)	1443 (33.0)	1393 (35.7)	878 (54.5)	515 (22.5)
**Ethnicity**						
Black	964 (27.7)	389 (24.7)	575 (30.1)	473 (19.1)	118 (13.9)	355 (21.8)
Caucasian	2282 (65.5)	1088 (69.2)	1194 (62.5)	1338 (54.0)	566 (66.9)	772 (47.3)
Other	237 (6.8)	96 (6.1)	141 (7.4)	667 (26.9)	162 (19.1)	505 (30.9)
**Past medical history**						
Prior heart failure	2943 (36.3)	2219 (56.3)	724 (17.3)	1199 (31.2)	884 (55.3)	315 (14.0)
Ischaemic heart disease	2632 (36.4)	1687 (49.7)	945 (24.7)	1150 (30.0)	746 (47.0)	404 (18.0)
Diabetes mellitus	1756 (26.5)	1029 (32.5)	727 (21.1)	1047 (27.0)	558 (34.7)	489 (21.5)
Hypertension	4167 (62.7)	2242 (72.4)	1925 (54.2)	2529 (65.4)	1241 (77.6)	1288 (56.8)
Hyperlipidemia	1350 (37.5)	751 (44.4)	599 (31.4)	1421 (40.2)	705 (49.5)	716 (33.9)
Current or ex-smoker	864 (31.7)	306 (26.0)	558 (35.9)	672 (27.5)	187 (22.5)	485 (30.1)
Asthma	372 (19.0)	47 (6.4)	325 (26.5)	488 (22.2)	44 (6.3)	444 (29.6)
COPD	2077 (34.4)	725 (26.1)	1352 (41.5)	1060 (27.5)	353 (22.2)	707 (31.3)
Atrial fibrillation	1380 (21.5)	1033 (32.6)	347 (10.6)	722 (19.8)	556 (34.8)	166 (8.1)
Chronic kidney disease	931 (22.2)	696 (34.9)	235 (10.7)	793 (20.7)	572 (36.1)	221 (9.8)
**Body mass index, kg/m^2^**						
<25	2503 (40.6)	1242 (41.3)	1261 (40.0)	1311 (40.1)	572 (39.6)	739 (40.5)
25–29	1727 (28.0)	892 (29.7)	835 (26.5)	973 (29.8)	474 (32.8)	499 (27.3)
≥30	1928 (31.3)	873 (29.0)	1055 (33.5)	986 (30.2)	398 (27.6)	588 (32.2)
**Physiological parameters**						
Heart rate, beats per minute	92.1 (23.4)	91.6 (25.4)	92.6 (21.3)	92.3 (23.3)	92.0 (26.0)	92.5 (21.1)
Systolic blood pressure, mmHg	139.8 (28.9)	140.7 (31.3)	139.0 (26.5)	139.6 (27.8)	140.4 (30.4)	139.0 (25.8)
Diastolic blood pressure, mmHg	79.2 (18.0)	79.9 (19.5)	78.5 (16.5)	80.7 (17.2)	81.6 (18.9)	80.1 (15.8)
**Clinical haematology and biochemistry**						
Haemoglobin, g/dL	13.1 (4.9)	12.7 (4.5)	13.4 (5.2)	13.1 (2.1)	12.8 (2.1)	13.4 (2.1)
eGFR, mL/min/1.73 m^2^	65.9 (30.5)	56.8 (27.6)	74.5 (30.6)	72.0 (32.0)	58.8 (29.4)	81.8 (30.3)
BNP, pg/mL	255.1 [60.0, 801.0]	729.0 [353.0, 1265.0]	70.4 [23.1, 189.0]	—	—	—
MR-proANP, pmol/L	—	—	—	191.0 [71.3, 385.0]	390.7 [266.8, 598.5]	87.5 [47.5, 175.6]

Presented as No. (%), mean (SD) or median [inter-quartile range].

Abbreviations: BNP, B-type natriuretic peptide; COPD, chronic obstructive pulmonary disease; eGFR, estimated glomerular filtration rate; MR-proANP, Mid-regional pro-atrial natriuretic peptide.

### Guideline-recommended BNP threshold

**Figure 1:**
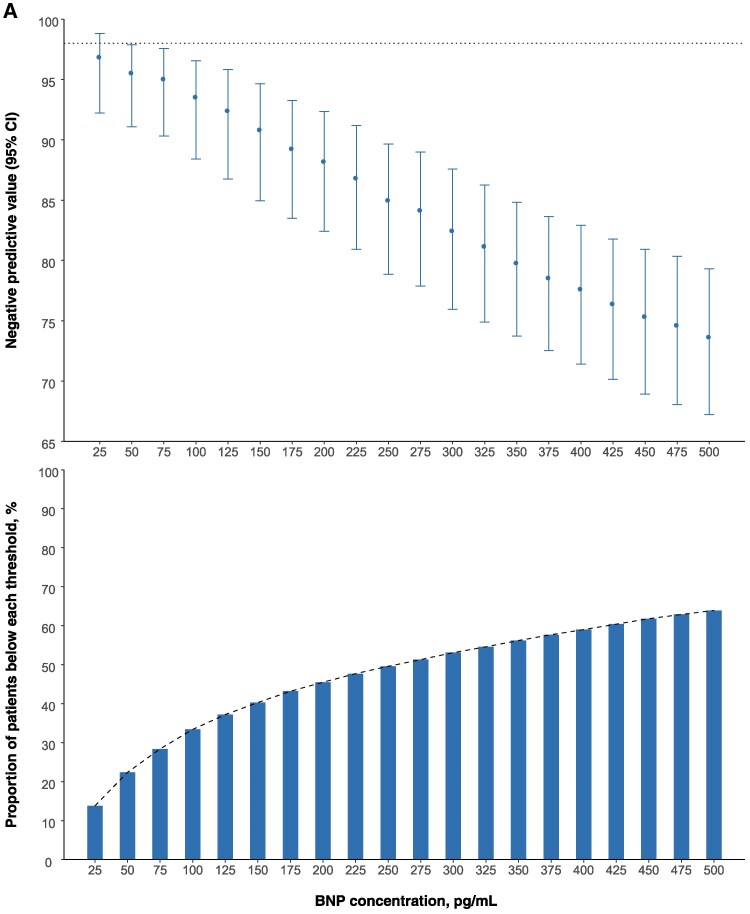

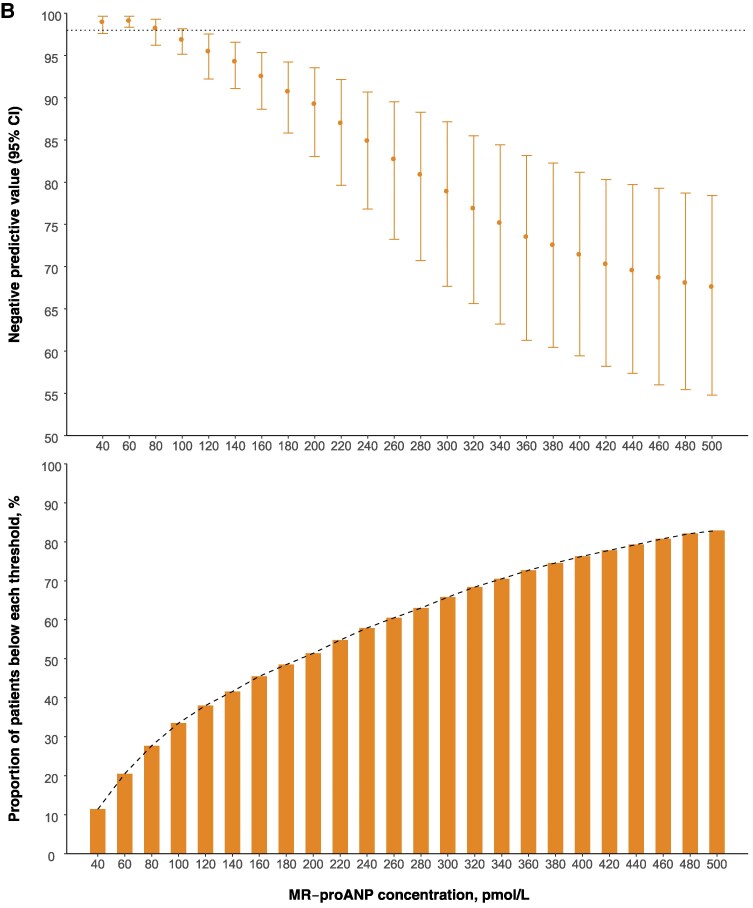
BNP and MR-proANP thresholds for acute heart failure. (*A*) (top) NPVs of BNP concentrations to rule-out a diagnosis of acute heart failure. (bottom) Cumulative proportion of patients presenting with suspected acute heart failure with BNP concentrations below each threshold. (*B*) (top) NPVs of MR-proANP concentrations to rule-out a diagnosis of acute heart failure. (bottom) Cumulative proportion of patients presenting with suspected acute heart failure with MR-proANP concentrations below each threshold. * dashed horizontal line corresponds to NPV of 98%.

**Figure 2:**
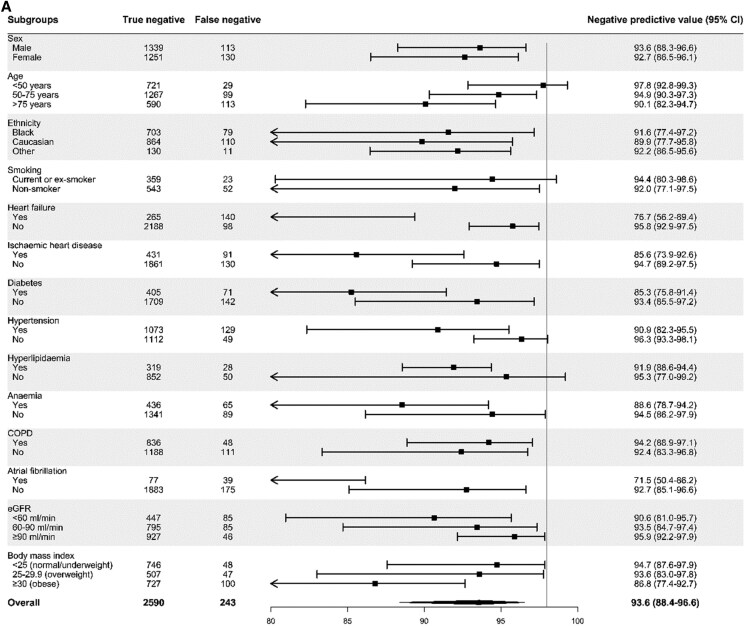

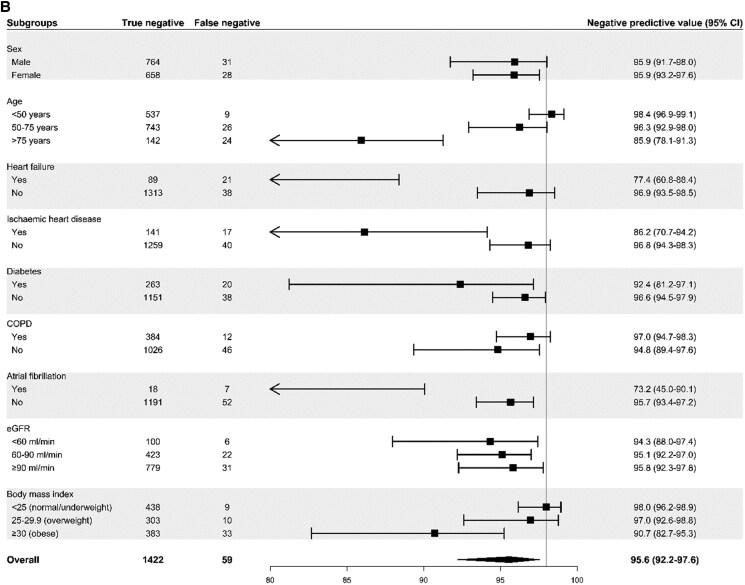
NPV of guideline-recommended BNP and MR-proANP thresholds across patient subgroups. (*A*) NPV of the BNP threshold of 100 pg/mL across patient subgroups. (*B*) NPV of the MR-proANP threshold of 120 pmol/L across patient subgroups. COPD, chronic obstructive pulmonary disease; eGFR, estimated glomerular filtration rate.

Pooled meta-estimates of NPV, sensitivity, PPV and specificity of the guideline-recommended BNP threshold of 100 pg/mL were 93.6% (95% CI, 88.4–96.6%), 96.0% (93.2–97.6%), 68.8% (62.9–74.2%), and 56.5% (48.4–64.3%) respectively (*Figure 1* and *Table 2*). The AUC for BNP as a continuous measure was 0.885 (0.878–0.892). BNP concentrations were below 100 pg/mL in 2833 (33%) patients. There was marked heterogeneity in the performance of this threshold across patient subgroups (*Figure 2*). The NPV and sensitivity was lower in those with prior heart failure [76.7% (56.2–89.4%) and 96.4% (92.7–98.3%)], atrial fibrillation [71.5% (50.4–86.2%) and 96.9% (93.7–98.5%)] and obesity [86.8% (77.4–92.7%) and 88.9% (84.1–92.4%)]. We subsequently evaluated alternative BNP thresholds and found that none achieved our pre-specified optimal rule-out criteria (NPV of 98% and sensitivity of 90%). The PPV of a BNP concentration ≥100 pg/mL was also heterogeneous with lower performance in patients without prior heart failure [56.0% (48.0–63.8%)], those with COPD [53.7% (38.2–68.5%)] and those with normal renal function [60.3% (52.3–67.8%)] (see Supplementary material online, *Figure S2*).

**Table 2:** Diagnostic performance of BNP, MR-proANP and CoDE-HF thresholds for acute heart failure

A. Rule-out thresholds and CoDE-HF scores.								
	Threshold or score	True positive	False positive	True negative	False negative	NPV (95% CI)	Sensitivity (95% CI)	Proportion ruled out
**All patients**								
BNP	100 pg/mL	3862	1798	2590	243	93.6 (88.4–96.6)	96.0 (93.2–97.6)	33%
MR-proANP	120 pmol/L	1552	866	1422	59	95.6 (92.2–97.6)	96.3 (95.3–97.2)	38%
**Patients without prior heart failure**								
CoDE-HF—BNP	5.4	1704	1943	1508	20	98.5 (97.1–99.3)	98.9 (98.0–99.3)	30%
CoDE-HF—MR-proANP	8.1	695	675	1259	19	98.6 (97.5–99.2)	97.9 (96.5–98.8)	48%

B. Rule-in thresholds and CoDE-HF scores.								
	Threshold or score	True positive	False positive	True negative	False negative	PPV (95% CI)	Specificity (95% CI)	Proportion ruled in
**All patients**								
BNP	100 pg/mL	3862	1798	2590	243	68.8 (62.9–74.2)	56.5 (48.4–64.3)	67%
MR-proANP	120 pmol/L	1552	866	1422	59	64.8 (56.3–72.5)	63.5 (54.4–71.7)	62%
**Patients without prior heart failure**								
CoDE-HF—BNP	58.0	1240	329	3122	484	78.6 (70.4–85.0)	90.2 (86.8–92.8)	30%
CoDE-HF—MR-proANP	46.0	548	179	1755	166	77.5 (72.6–81.7)	90.0 (84.1–93.9)	28%
**Patients with prior heart failure**								
CoDE-HF—BNP	90.7	1093	60	664	1126	94.9 (90.9–97.1)	92.6 (87.7–95.7)	39%
CoDE-HF—MR-proANP	91.7	459	25	290	425	95.7 (93.3–97.2)	90.4 (73.6–96.9)	40%

### Guideline-recommended MR-proANP threshold

Pooled meta-estimates of NPV, sensitivity, PPV and specificity of the guideline-recommended MR-proANP threshold of 120 pmol/L were 95.6% (92.2–97.6%), 96.3% (95.3–97.2%), 64.8% (56.3–72.5%), and 63.5% (54.4–71.7%), respectively (*Figure 1* and *Table 2*). The AUC for MR-proANP as a continuous measure was 0.901 (0.891–0.910). MR-proANP concentrations were below 120 pmol/L in 1481 (38%) patients. Similar to BNP, there was marked heterogeneity in the performance of this threshold across patient subgroups (*Figure 2*). NPV was lower in those with prior heart failure [77.4% (60.8–88.4%)] and atrial fibrillation [73.2% (45.0–90.1%)], and the NPV and sensitivity were lower in those with obesity [90.7% (82.7–95.3%) and 91.7% (88.6–94.0%)]. A lower MR-proANP threshold of 80 pmol/L achieved our pre-specified optimal rule-out criteria (NPV of 98% and sensitivity of 90%) and ruled out 1079 (28%) patients. However, performance remained heterogeneous across patient subgroups (see Supplementary material online, *Figure S3*). The PPV of an MR-proANP concentration ≥120 pmol/L was also heterogeneous with lower PPV in patients without prior heart failure [53.1% (44.1–62.0%)] or atrial fibrillation [59.5% (54.2–64.6%)], and in those with COPD [50.0% (40.7–59.3%)] (see Supplementary material online, *Figure S2*).

### The CoDE-HF score

**Figure 3:**
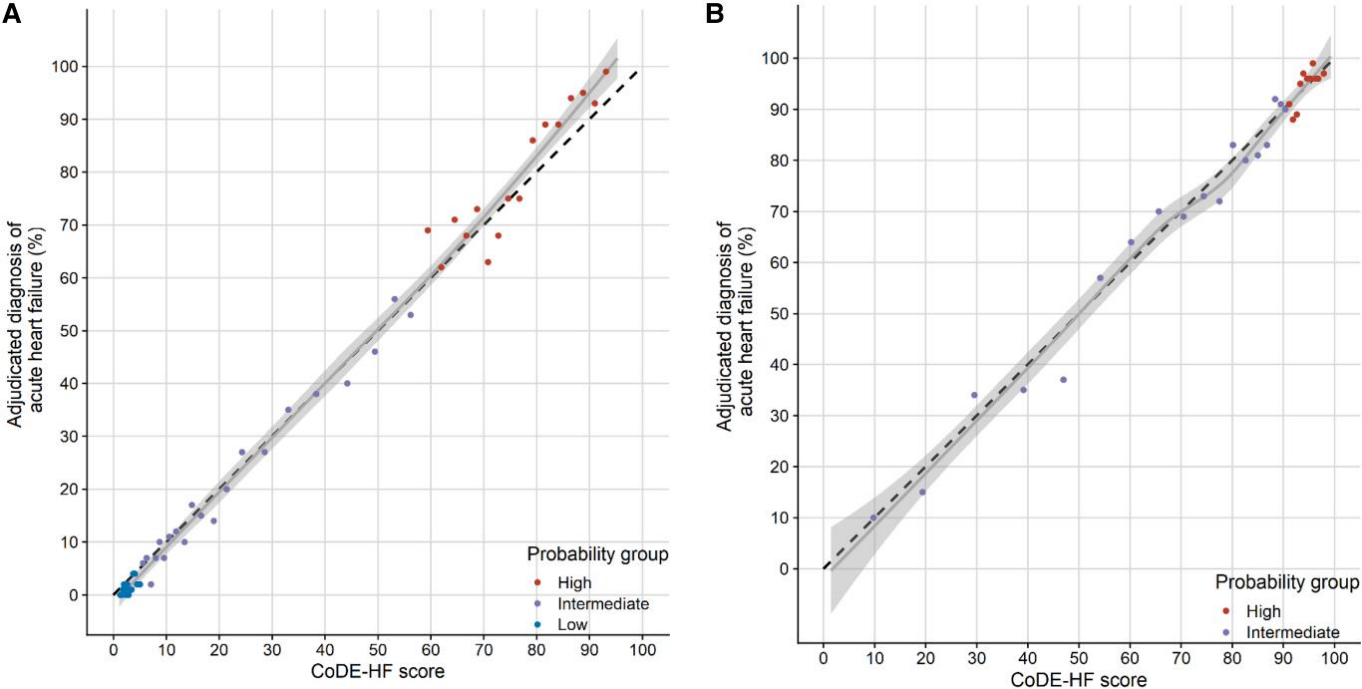
Calibration plot of CoDE-HF with BNP in patients with (*A*) no previous heart failure and (*B*) previous heart failure.

CoDE-HF with BNP had an AUC of 0.914 (0.906–0.921) and a Brier score of 0.110 in patients without prior heart failure and an AUC of 0.848 (0.831–0.864) and Brier score of 0.123 in those with prior heart failure (*Figure 3* and Supplementary material online, *Figure S4*). CoDE-HF with MR-proANP achieved an AUC 0.929 (0.919–0.939) and Brier score of 0.094 in patients without prior heart failure, and AUC 0.857 (0.831–0.882) and Brier score of 0.122 in patients with prior heart failure (see Supplementary material online, *Figures S5*–S6).

**Figure 4:**
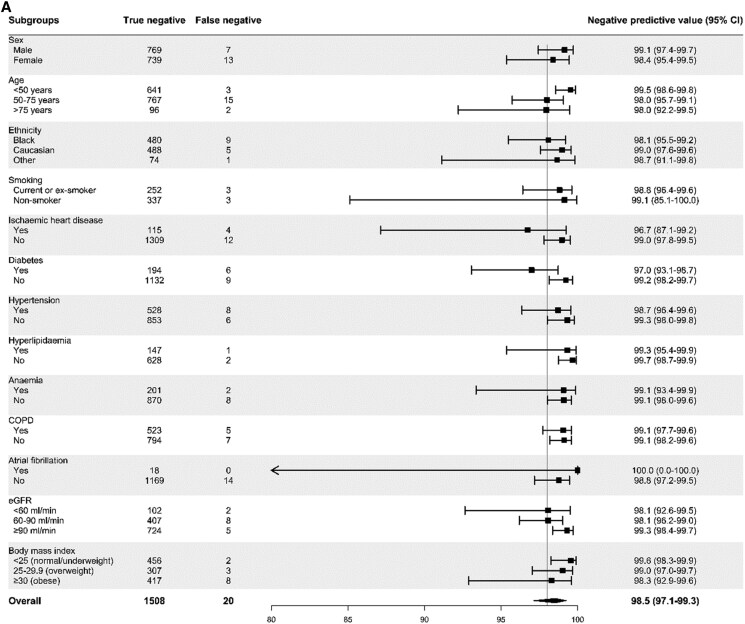

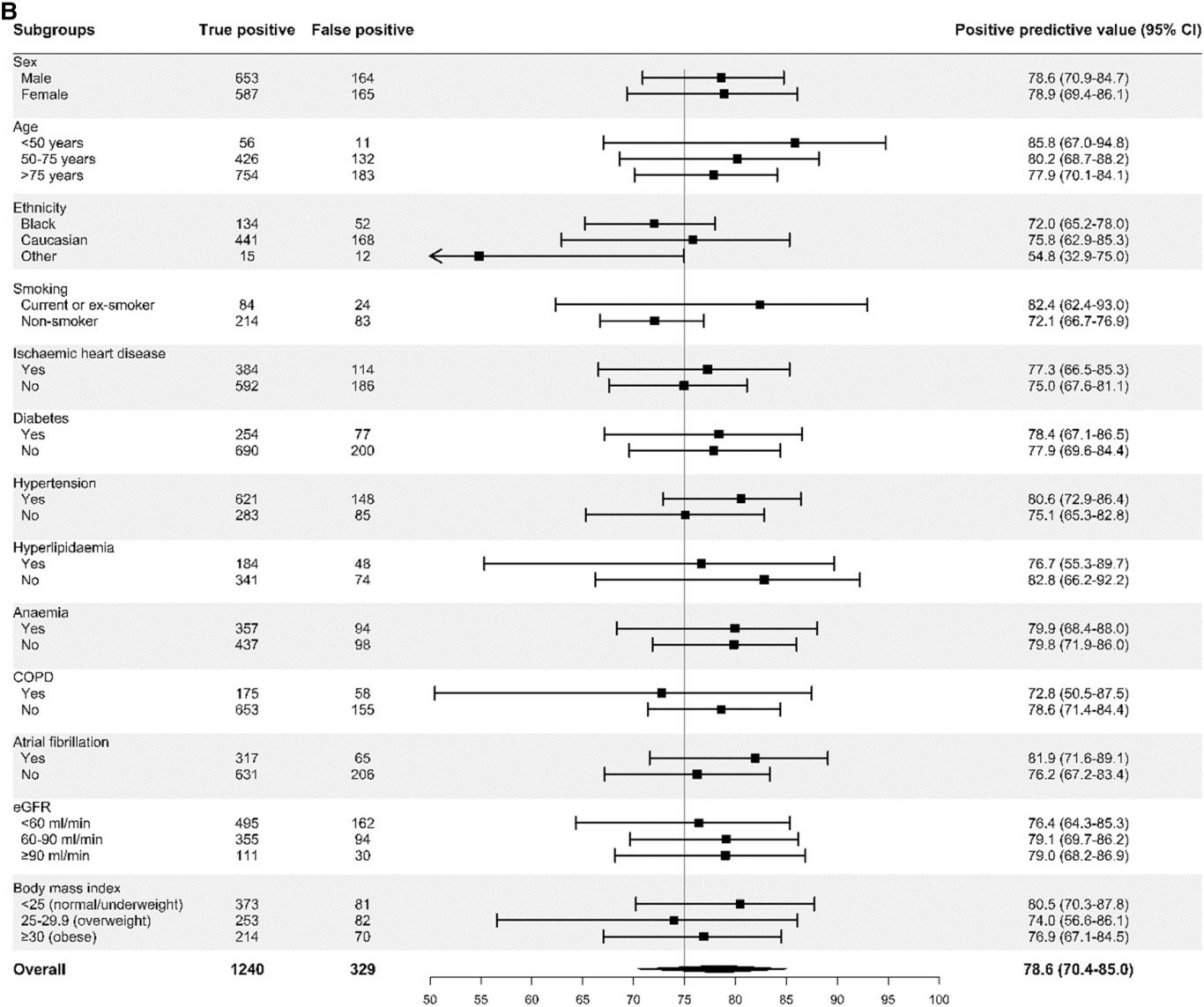

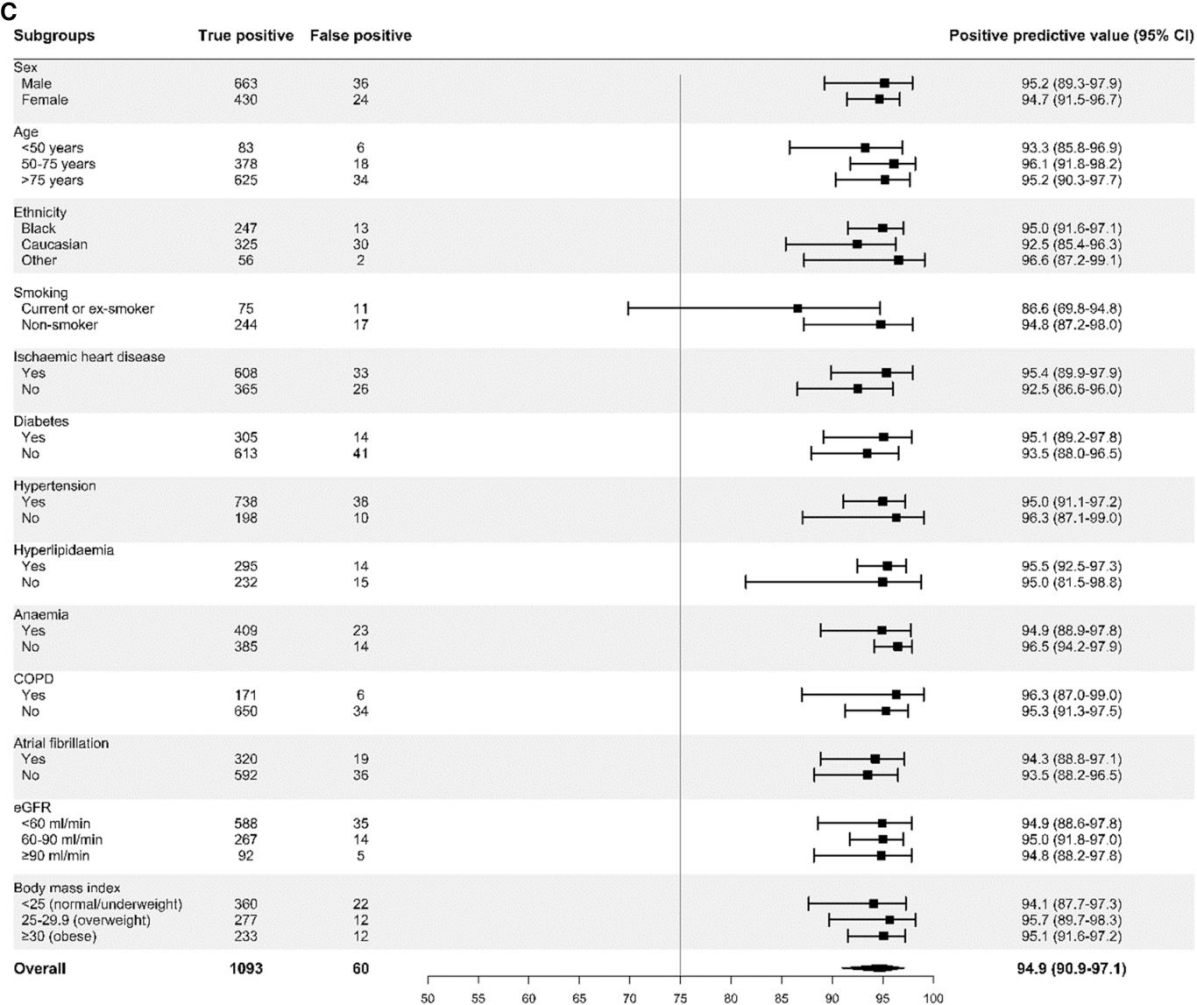
Diagnostic performance of the CoDE-HF score across patient subgroups. CoDE-HF incorporates BNP concentrations as a continuous measure and predefined simple objective clinical variables (age, eGFR, haemoglobin, body mass index, heart rate, blood pressure, peripheral oedema, prior history of heart failure, COPD and ischaemic heart disease) to provide an individualized assessment of the likelihood of the diagnosis of acute heart failure. (*A*) NPV of the CoDE-HF rule-out score of 5.4 in patients without prior heart failure across patient subgroups. (*B*) PPV of the CoDE-HF rule-in score of 58.0 in patients without prior heart failure across patient subgroups. (*C*) PPV of the CoDE-HF rule-in score of 90.7 in patients with prior heart failure across patient subgroups.

For BNP, a CoDE-HF score of 5.4 achieved an NPV of 98.5% (97.1–99.3%) and a sensitivity of 98.9% (98.0–99.3%), whilst a score of 58.0 achieved a PPV of 78.6% (70.4–85.0%) and a specificity of 90.2% (86.8–92.8%) in those without prior heart failure (*Table 2* and Supplementary material online, *Table S5*). These rule-out and rule-in scores had a more consistent performance across all subgroups compared with BNP thresholds (*Figure 4*). If these scores were applied in patients without prior heart failure, CoDE-HF with BNP would identify 30% as low-probability and 30% as high-probability of acute heart failure, respectively. In patients with prior heart failure, no score achieved our target rule-out criteria in the training cohort. A CoDE-HF score of 90.7 achieved a PPV of 94.9% (90.9–97.1%) and a specificity of 92.6% (87.7–95.7%) (*Figure 4*).

For MR-proANP, a CoDE-HF score of 8.1 achieved an NPV of 98.5% (97.7–99.0%) and sensitivity of 97.3% (95.5–98.4%), whilst a score of 46.0 achieved a PPV of 75.1% (70.9–78.9%) and a specificity of 90.4% (86.1–93.5%) in those without prior heart failure (*Table 2* and Supplementary material online, *Table S6*). Similarly, these rule-out and rule-in scores had more consistent performance across subgroups than the biomarker threshold alone (see Supplementary material online, *Figure S7*). If these scores were applied in patients without prior heart failure, CoDE-HF with MR-proANP would identify 48% as low-probability and 28% as high-probability of acute heart failure. In patients with prior heart failure, a CoDE-HF score of 91.7 achieved a PPV of 94.2% (89.5–96.9%) and a specificity of 90.1% (81.4–95.0%) (see Supplementary material online, *Figure S7*).

In a decision curve analysis, CoDE-HF had a superior net benefit compared with the BNP and MR-proANP alone across all threshold probabilities (see Supplementary material online, *Figure S8*). Internal-external cross-validation demonstrated good performance across cohorts for all models (see Supplementary material online, *Figures S9*–S10).

Patients who were identified as low-probability by CoDE-HF had a substantially lower rate of all-cause mortality at 30-days and 1 year compared with those who were identified as intermediate and high-probability for both BNP (30-day all-cause mortality: 0.8% vs. 5.1% and 11.5%; 1 year all-cause mortality: 7.0% vs. 21.9% and 34.6%, respectively) and MR-proANP (30-day all-cause mortality: 1.0% vs. 5.6% and 8.9%; 1 year all-cause mortality: 5.8% vs. 19.8% and 30.6%, respectively) (see Supplementary material online, *Figure S11*).

## Discussion

In this individual patient-level meta-analysis, we evaluated the diagnostic performance of guideline-recommended BNP and MR-proANP thresholds in over 9303 patients across 14 studies, and subsequently developed and validated a decision-support tool that uses these natriuretic peptides as a continuous variable with patient factors for the diagnosis of acute heart failure. Several findings are reported that could affect clinical practice. First, the guideline-recommended thresholds of BNP and MR-proANP to rule out acute heart failure had heterogeneous performance across important patient subgroups. NPV was substantially lower in those with prior heart failure, atrial fibrillation, and ischaemic heart disease where false negative rates were as high as one in five. Second, there was no threshold at which BNP achieved an NPV of 98%. For MR-proANP, an optimized threshold of 80 pmol/L achieved an NPV of 98%; however, performance remained heterogenous across patient subgroups. Finally, the CoDE-HF decision-support tool was developed and validated for BNP and MR-proANP using machine learning to combine these natriuretic peptides with simple and objective patient factors to calculate an individualized probability of acute heart failure. CoDE-HF had a more consistent performance across patient subgroups compared with BNP or MR-proANP thresholds alone.

This is the largest study using pooled data to evaluate the diagnostic performance of BNP and MR-proANP for acute heart failure to date. All studies confirmed the diagnosis of acute heart failure using a standardized adjudication process. The availability of individual patient-level data allowed us to evaluate the performance of guideline-recommended thresholds across patient subgroups. Furthermore, this enabled the evaluation of these natriuretic peptides across a range of alternative thresholds and the development of a decision-support tool using machine learning.

We have previously developed the CoDE-HF decision-support tool using NT-proBNP.^12^ We have now further developed CoDE-HF for BNP and MR-proANP and demonstrate that the use of machine learning improves the diagnostic performance of all three natriuretic peptides. This is intuitive given that all natriuretic peptides share a similar mechanism of release from the myocardium in response to myocardial pressure and volume overload, and are similarly influenced by patient factors such as age, heart rhythm, renal function and obesity.^32–38^ This is particularly important given the increasing prevalence of heart failure in ageing populations with an increasing number of comorbidities. The availability of a simple decision-support tool that incorporates routinely collected clinical variables to aid in the interpretation of these biomarkers could improve the efficiency and accuracy of the assessment of patients in busy emergency departments.

CoDE-HF has the potential to improve equity of care and patient outcomes by accurately identifying those who would benefit from expedited treatment, specialist referrals and investigations such as echocardiography in patients with a high-probability of the diagnosis. Indeed, recent randomized-controlled trial evidence shows that many treatments for heart failure result in rapid onset of benefit and prompt initiation of evidence-based therapies can result in improved outcomes for patients with heart failure.^39–41^ Patients with a low-probability of acute heart failure could be discharged from the Emergency Department safely or investigated for other differential diagnoses more promptly resulting in cost savings for healthcare institutions. Furthermore, different thresholds of CoDE-HF score to identify those at high- and low-probability of acute heart failure can be selected by individual healthcare institutions based on the availability of local resources and tolerance for risk. Since CoDE-HF utilizes routinely collected variables, it can be embedded within the electronic patient records to facilitate more accurate and efficient patient assessment.

We are aware of numerous validated prognostic risk scores for patients with an established diagnosis of heart failure.^31,42,43^ However, there are only a few that have been developed to aid in the diagnosis of acute heart failure.^44,45^ Whilst these diagnostic scores have many strengths, they incorporate more subjective variables such as the clinicians’ estimation of the pre-test probability, patients’ description of symptoms, and natriuretic peptides as a binary variable, which does not take into account the dynamic and non-linear interaction between natriuretic peptides and other measures. These previous attempts at developing and validating diagnostic scores have also included a limited number of patients from a single healthcare setting, which precluded the assessment of diagnostic performance within important patient subgroups and limits external generalisability.

Several potential limitations should be considered in this study. First, acute heart failure is ultimately a clinical diagnosis and therefore, it is likely that there is some inherent heterogeneity in the adjudication of this diagnosis across different studies. Second, the adjudicated diagnosis of acute heart failure did not differentiate between the different underlying aetiologies of heart failure or between heart failure with reduced ejection fraction, heart failure with mildly reduced ejection fraction, and heart failure with preserved ejection fraction. Nevertheless, the CoDE-HF decision-support tool was designed to aid in the initial triage of all patients with suspected acute heart failure regardless of aetiology. Our approach aligns with how a diagnostic tool is used in acute care and the emergency department. Further testing and determination of ejection fraction occurs after an acute heart failure diagnosis is made. Third, the prevalence of acute heart failure varies significantly across studies and may have influenced the diagnostic performance of BNP, MR-proANP and CoDE-HF. This heterogeneity reflects the diverse range of settings and populations in which natriuretic peptides and the decision-support tool will be applied in clinical practice and strengthens the generalizability of the study findings.^46^ However, further prospective validation in consecutive patient populations would be useful. Finally, there is significant missingness in some of the studies included in this analysis. Where possible, multiple imputation was performed to maximize the use of data in the development of the machine learning model.

## Conclusion

Guideline-recommended thresholds of BNP and MR-proANP have heterogeneous performance across important patient subgroups. The CoDE-HF decision-support tool was developed and validated for BNP and MR-proANP and ruled-in and ruled-out acute heart failure more accurately than natriuretic peptide thresholds alone.

## Supplementary Material

zuaf051_Supplementary_Data

## Data Availability

The R code to develop and validate the CoDE-HF score can be made available to academic researchers upon request to the corresponding author. Deidentified individual participant data can be made available to researchers subject to approval of the principal investigators of the individual studies included in this analysis.

## References

[1] National Heart Failure Audit 2019 Report. University College London: NICOR (National Institute for Cardiovascular Outcomes Research). 2019. https://www.nicor.org.uk/wp-content/uploads/2019/09/Heart-Failure-2019-Report-final.pdf.

[2] Pang PS, Collins SP, Miró Ò, Bueno H, Diercks DB, Di Somma S, et al Editor’s choice-the role of the emergency department in the management of acute heart failure: an international perspective on education and research. Eur Heart J Acute Cardiovasc Care 2017;6:421–429. 26265736 10.1177/2048872615600096

[3] Roberts E, Ludman AJ, Dworzynski K, Al-Mohammad A, Cowie MR, McMurray JJV, et al The diagnostic accuracy of the natriuretic peptides in heart failure: systematic review and diagnostic meta-analysis in the acute care setting. BMJ 2015;350:h910. 25740799 10.1136/bmj.h910 PMC4353288

[4] National Institute for Health and Care Excellence. Acute heart failure: diagnosis and management. Clinical Guideline 187. 2014. https://www.nice.org.uk/guidance/cg187.

[5] Januzzi JL Jr, Chen-Tournoux AA, Christenson RH, Doros G, Hollander JE, Levy PD, et al N-Terminal Pro-B-Type natriuretic peptide in the emergency department: the ICON-RELOADED study. J Am Coll Cardiol 2018;71:1191–1200. 29544601 10.1016/j.jacc.2018.01.021

[6] McDonagh TA, Metra M, Adamo M, Gardner RS, Baumbach A, Böhm M, et al 2021 ESC guidelines for the diagnosis and treatment of acute and chronic heart failure: developed by the task force for the diagnosis and treatment of acute and chronic heart failure of the European Society of Cardiology (ESC) with the special contribution of the heart failure association (HFA) of the ESC. Eur J Heart Fail 2022;24:4–131. 35083827 10.1002/ejhf.2333

[7] Mueller C, McDonald K, de Boer RA, Maisel A, Cleland JGF, Kozhuharov N, et al Heart failure association of the European Society of Cardiology practical guidance on the use of natriuretic peptide concentrations. Eur J Heart Fail 2019;21:715–731. 31222929 10.1002/ejhf.1494

[8] Yancy CW, Jessup M, Bozkurt B, Butler J, Casey DE, Colvin MM, et al 2017 ACC/AHA/HFSA focused update of the 2013 ACCF/AHA guideline for the management of heart failure: a report of the American College of Cardiology/American Heart Association task force on clinical practice guidelines and the heart failure society of America. Circulation 2017;136:e137–e161. 28455343 10.1161/CIR.0000000000000509

[9] Baggish AL, van Kimmenade RR, Januzzi JL Jr. The differential diagnosis of an elevated amino-terminal pro-B-type natriuretic peptide level. Am J Cardiol 2008;101:43–48. 18243858 10.1016/j.amjcard.2007.11.019

[10] Christiansen MN, Kober L, Weeke P, Vasan RS, Jeppesen JL, Smith JG, et al Age-specific trends in incidence, mortality, and comorbidities of heart failure in Denmark, 1995 to 2012. Circulation 2017;135:1214–1223. 28174193 10.1161/CIRCULATIONAHA.116.025941

[11] Kozhuharov N, Sabti Z, Wussler D, Nowak A, Badertscher P, Twerenbold R, et al Prospective validation of N-terminal pro B-type natriuretic peptide cut-off concentrations for the diagnosis of acute heart failure. Eur J Heart Fail 2019;21:813–815. 31020757 10.1002/ejhf.1471

[12] Lee KK, Doudesis D, Anwar M, Astengo F, Chenevier-Gobeaux C, Claessens Y-E, et al Development and validation of a decision support tool for the diagnosis of acute heart failure: systematic review, meta-analysis, and modelling study. BMJ 2022;377:e068424. 35697365 10.1136/bmj-2021-068424 PMC9189738

[13] DerSimonian R, Laird N. Meta-analysis in clinical trials. Control Clin Trials 1986;7:177–188. 3802833 10.1016/0197-2456(86)90046-2

[14] Chen T, Guestrin C. XGBoost: A Scalable Tree Boosting System. ArXiv . 10.1145/2939672.2939785, 9 March 2016, preprint: not peer reviewed.

[15] Quartagno M, Carpenter JR. Multiple imputation for IPD meta-analysis: allowing for heterogeneity and studies with missing covariates. Stat Med 2016;35:2938–2954. 26681666 10.1002/sim.6837 PMC5064632

[16] Steyerberg EW, Vickers AJ, Cook NR, Gerds T, Gonen M, Obuchowski N, et al Assessing the performance of prediction models: a framework for traditional and novel measures. Epidemiology 2010;21:128–138. 20010215 10.1097/EDE.0b013e3181c30fb2 PMC3575184

[17] Debray TP, Riley RD, Rovers MM, Reitsma JB, Moons KG. Individual participant data (IPD) meta-analyses of diagnostic and prognostic modeling studies: guidance on their use. PLoS Med 2015;12:e1001886. 26461078 10.1371/journal.pmed.1001886 PMC4603958

[18] Bahrmann P, Bahrmann A, Hofner B, Christ M, Achenbach S, Sieber CC, et al Multiple biomarker strategy for improved diagnosis of acute heart failure in older patients presenting to the emergency department. Eur Heart J Acute Cardiovasc Care 2015;4:137–147. 25002708 10.1177/2048872614541904

[19] Behnes M, Brueckmann M, Ahmad-Nejad P, Lang S, Wolpert C, Elmas E, et al Diagnostic performance and cost effectiveness of measurements of plasma N-terminal pro brain natriuretic peptide in patients presenting with acute dyspnea or peripheral edema. Int J Cardiol 2009;135:165–174. 18603317 10.1016/j.ijcard.2008.03.045

[20] Bombelli M, Maloberti A, Rossi S, Rea F, Corrao G, Bonicelli Della Vite C, et al Clinical value of NT-proBNP assay in the emergency department for the diagnosis of heart failure (HF) in very elderly people. Arch Gerontol Geriatr 2015;61:296–300. 25991044 10.1016/j.archger.2015.05.001

[21] Chenevier-Gobeaux C, Claessens YE, Voyer S, Desmoulins D, Ekindjian OG. Influence of renal function on N-terminal pro-brain natriuretic peptide (NT-proBNP) in patients admitted for dyspnoea in the emergency department: comparison with brain natriuretic peptide (BNP). Clin Chim Acta 2005;361:167–175. 15993397 10.1016/j.cccn.2005.05.021

[22] deFilippi CR, Seliger SL, Maynard S, Christenson RH. Impact of renal disease on natriuretic peptide testing for diagnosing decompensated heart failure and predicting mortality. Clin Chem 2007;53:1511–1519. 17586595 10.1373/clinchem.2006.084533

[23] Gargani L, Frassi F, Soldati G, Tesorio P, Gheorghiade M, Picano E, et al Ultrasound lung comets for the differential diagnosis of acute cardiogenic dyspnoea: a comparison with natriuretic peptides. Eur J Heart Fail 2008;10:70–77. 18077210 10.1016/j.ejheart.2007.10.009

[24] Ibrahim I, Kuan WS, Frampton C, Troughton R, Liew OW, Chong JPC, et al Superior performance of N-terminal pro brain natriuretic peptide for diagnosis of acute decompensated heart failure in an Asian compared with a western setting. Eur J Heart Fail 2017;19:209–217. 27620387 10.1002/ejhf.612

[25] Januzzi JL, van Kimmenade R, Lainchbury J, Bayes-Genis A, Ordonez-Llanos J, Santalo-Bel M, et al NT-proBNP testing for diagnosis and short-term prognosis in acute destabilized heart failure: an international pooled analysis of 1256 patients: the international collaborative of NT-proBNP study. Eur Heart J 2006;27:330–337. 16293638 10.1093/eurheartj/ehi631

[26] Maisel A, Mueller C, Nowak R, Peacock WF, Landsberg JW, Ponikowski P, et al Mid-region pro-hormone markers for diagnosis and prognosis in acute dyspnea: results from the BACH (biomarkers in acute heart failure) trial. J Am Coll Cardiol 2010;55:2062–2076. 20447528 10.1016/j.jacc.2010.02.025

[27] Moe GW, Howlett J, Januzzi JL, Zowall H. Canadian multicenter improved management of patients with congestive heart failure study I. N-terminal pro-B-type natriuretic peptide testing improves the management of patients with suspected acute heart failure: primary results of the Canadian prospective randomized multicenter IMPROVE-CHF study. Circulation 2007;115:3103–3110. 17548729 10.1161/CIRCULATIONAHA.106.666255

[28] Mueller T, Gegenhuber A, Poelz W, Haltmayer M. Diagnostic accuracy of B type natriuretic peptide and amino terminal proBNP in the emergency diagnosis of heart failure. Heart 2005;91:606–612. 15831643 10.1136/hrt.2004.037762 PMC1768863

[29] Nazerian P, Vanni S, Zanobetti M, Polidori G, Pepe G, Federico R, et al Diagnostic accuracy of emergency Doppler echocardiography for identification of acute left ventricular heart failure in patients with acute dyspnea: comparison with Boston criteria and N-terminal prohormone brain natriuretic peptide. Acad Emerg Med 2010;17:18–26. 20078435 10.1111/j.1553-2712.2009.00630.x

[30] Rutten JH, Steyerberg EW, Boomsma F, van Saase JLCM, Deckers JW, Hoogsteden HC, et al N-terminal pro-brain natriuretic peptide testing in the emergency department: beneficial effects on hospitalization, costs, and outcome. Am Heart J 2008;156:71–77. 18585499 10.1016/j.ahj.2008.02.021

[31] Wussler D, Kozhuharov N, Sabti Z, Walter J, Strebel I, Scholl L, et al External validation of the MEESSI acute heart failure risk score: a cohort study. Ann Intern Med 2019;170:248–256. 30690646 10.7326/M18-1967

[32] Das SR, Drazner MH, Dries DL, Vega GL, Stanek HG, Abdullah SM, et al Impact of body mass and body composition on circulating levels of natriuretic peptides: results from the Dallas heart study. Circulation 2005;112:2163–2168. 16203929 10.1161/CIRCULATIONAHA.105.555573

[33] Levin ER, Gardner DG, Samson WK. Natriuretic peptides. N Engl J Med 1998;339:321–328. 9682046 10.1056/NEJM199807303390507

[34] McCullough PA, Duc P, Omland T, McCord J, Nowak RM, Hollander JE, et al B-type natriuretic peptide and renal function in the diagnosis of heart failure: an analysis from the breathing not properly multinational study. Am J Kidney Dis 2003;41:571–579. 12612980 10.1053/ajkd.2003.50118

[35] Mehra MR, Uber PA, Park MH, Scott RL, Ventura HO, Harris BC, et al Obesity and suppressed B-type natriuretic peptide levels in heart failure. J Am Coll Cardiol 2004;43:1590–1595. 15120816 10.1016/j.jacc.2003.10.066

[36] Takami Y, Horio T, Iwashima Y, Takiuchi S, Kamide K, Yoshihara F, et al Diagnostic and prognostic value of plasma brain natriuretic peptide in non-dialysis-dependent CRF. Am J Kidney Dis 2004;44:420–428. 15332214

[37] Tsutamoto T, Wada A, Sakai H, Ishikawa C, Tanaka T, Hayashi M, et al Relationship between renal function and plasma brain natriuretic peptide in patients with heart failure. J Am Coll Cardiol 2006;47:582–586. 16458140 10.1016/j.jacc.2005.10.038

[38] Wang TJ, Larson MG, Levy D, Benjamin EJ, Leip EP, Wilson PWF, et al Impact of obesity on plasma natriuretic peptide levels. Circulation 2004;109:594–600. 14769680 10.1161/01.CIR.0000112582.16683.EA

[39] Mebazaa A, Davison B, Chioncel O, Cohen-Solal A, Diaz R, Filippatos G, et al Safety, tolerability and efficacy of up-titration of guideline-directed medical therapies for acute heart failure (STRONG-HF): a multinational, open-label, randomised, trial. Lancet 2022;400:1938–1952. 36356631 10.1016/S0140-6736(22)02076-1

[40] Kondo T, Jhund PS, McMurray JJV. Drug therapy for heart failure with reduced ejection fraction: what is the ‘right’ dose? Eur J Heart Fail 2022;24:421–430. 35119172 10.1002/ejhf.2447 PMC9303189

[41] Vaduganathan M, Claggett BL, Jhund P, de Boer RA, Hernandez AF, Inzucchi SE, et al Time to clinical benefit of dapagliflozin in patients with heart failure with mildly reduced or preserved ejection fraction: a prespecified secondary analysis of the DELIVER randomized clinical trial. JAMA Cardiol 2022;7:1259–1263. 36190011 10.1001/jamacardio.2022.3750 PMC9531091

[42] Pocock SJ, Ariti CA, McMurray JJ, Maggioni A, Køber L, Squire IB, et al Predicting survival in heart failure: a risk score based on 39 372 patients from 30 studies. Eur Heart J 2013;34:1404–1413. 23095984 10.1093/eurheartj/ehs337

[43] Rahimi K, Bennett D, Conrad N, Williams TM, Basu J, Dwight J, et al Risk prediction in patients with heart failure: a systematic review and analysis. JACC Heart Fail 2014;2:440–446. 25194291 10.1016/j.jchf.2014.04.008

[44] Baggish AL, Siebert U, Lainchbury JG, Cameron R, Anwaruddin S, Chen A, et al A validated clinical and biochemical score for the diagnosis of acute heart failure: the ProBNP investigation of dyspnea in the emergency department (PRIDE) acute heart failure score. Am Heart J 2006;151:48–54. 16368291 10.1016/j.ahj.2005.02.031

[45] Steinhart B, Thorpe KE, Bayoumi AM, Moe G, Januzzi JL, Mazer CD. Improving the diagnosis of acute heart failure using a validated prediction model. J Am Coll Cardiol 2009;54:1515–1521. 19815122 10.1016/j.jacc.2009.05.065

[46] Searle J, Frick J, Mockel M. Acute heart failure facts and numbers: acute heart failure populations. ESC Heart Fail 2016;3:65–70. 27818780 10.1002/ehf2.12092 PMC5074292

