# Supplementary material for: Machine learning to optimize use of natriuretic peptides in the diagnosis of acute heart failure
Source: Eur Heart J Acute Cardiovasc Care. 2025 Apr 12;14(8):474–88. doi: 10.1093/ehjacc/zuaf051 (PMC12342151; doi:10.1093/ehjacc/zuaf051)
Supplement: zuaf051_Supplementary_Data [file zuaf051_supplementary_data.docx]

##### Supplement

Machine learning to optimise use of natriuretic peptides

in the diagnosis of acute heart failure

Dimitrios Doudesis, PhD^1, 2^*, Kuan Ken Lee, MD^1^*, Mohamed Anwar, MD^1^, Adam J Singer, MD^3^,
Judd E Hollander, MD^4^, Camille Chenevier-Gobeaux, PharmD PhD^5^, Yann-Erick Claessens, MD^6^, Desiree Wussler, MD^7, 8^, Dominic Weil, MD^7^, Nikola Kozhuharov, MD^7, 9^, Ivo Strebel, PhD^7^, Zaid Sabti, MD^7^, Christopher deFilippi, MD^10^, Stephen Seliger, MD^11^, Evandro Tinoco Mesquita, MD^12^, Jan C Wiemer, PhD^13^, Martin Möckel, MD^14^, Joel Coste, MD^15^, Patrick Jourdain, MD^16^, Komukai Kimiaki, MD^17^, Michihiro Yoshimura, MD^17^, Irwani Ibrahim, MD^18^, Shirley Beng Suat Ooi, MD^18^, Win Sen Kuan, MD^18^, Alfons Gegenhuber, MD^19^ Thomas Mueller, MD^20^, Olivier Hanon, MD^21^, Jean-Sébastien Vidal, MD^21^, Peter Cameron, MD^22^, Louisa Lam, PhD^22, 23^, Ben Freedman, MB^24^, Tommy Chung, PhD^25^, Sean P Collins, MD^26^, Christopher J Lindsell, PhD^27^, David E Newby, MD^1^, Alan G Japp, MD^1^, Anoop SV Shah, MD^1, 28^, Humberto Villacorta, MD^12^, A Mark Richards, MD^29, 30^, John JV McMurray, MD^31^, Christian Mueller, MD^7^, James L Januzzi, MD^32, 33^ and Nicholas L Mills, MD^1, 2^

*on behalf of the CoDE-HF investigators^†^*

**Corresponding Author:**

Dr Dimitrios Doudesis

BHF/University Centre for Cardiovascular Science

The University of Edinburgh

Edinburgh EH16 4SA

United Kingdom

E-mail: [Dimitrios.Doudesis@ed.ac.uk](mailto:Dimitrios.Doudesis@ed.ac.uk)

**Supplementary Texts:** 2

**Supplementary Tables:** 6

**Supplementary Figures:** 11

# **I. List of Investigators.**

**CoDE-HF Investigators**

**Chief Investigator:** Prof Nicholas L Mills, MD^1,2^

**Research team:** Dr Dimitrios Doudesis, PhD,^1,2^ Dr Kuan Ken Lee, MD,^1^ Dr Mohammed Anwar, MD,^1^ Ms Federica Astengo, BSc,^1^ Prof David E Newby, MD,^1^ Dr Alan Japp, MD,^1^ Dr Anoop SV Shah, MD.^1,3^

**Collaborators:** Prof Adam Singer, MD,^4^ Prof Judd Hollander, MD,^5^ Dr Camille Chenevier-Gobeaux, PharmD PhD,^6^ Prof Yann-Erick Claessens, MD,^7^ Dr Desiree Wussler, MD,^8,9^ Dr Nikola Kozhuharov, MD,^8,10^ Dr Ivo Strebel, PhD,^8^ Dr Zaid Sabti, MD,^8^ Dr Christopher deFilippi, MD,^11^ Dr Stephen Seliger, MD,^12^ Prof Gordon Moe, MD,^13^ Dr Carlos Fernando, MD,^13^ Prof Evandro Tinoco Mesquita, MD PhD,^14^ Prof Antoni Bayes-Genis, MD,^15^ Dr Roland RJ van Kimmenade, MD,^16^ Prof Yigal Pinto, MD,^17^ Dr Hannah K Gaggin, MD,^18,19^ Dr Jan C Wiemer, PhD,^20^ Prof Martin Möckel, MD,^21^ Dr Joel Coste, MD PhD,^22^ Prof Patrick Jourdain, MD,^23^ Dr Joost HW Rutten, MD,^24^ Dr Anton H. van den Meiracker, MD,^25^ Dr Komukai Kimiaki, MD,^26^ Dr Michihiro Yoshimura, MD,^26^ Dr Luna Gargani, MD,^27^ Dr Nicola R Pugliese, MD,^28^ Dr Christopher Pemberton, PhD,^29^ Dr Irwani Ibrahim, MD,^30^ Dr Alfons Gegenhuber, MD,^31^ Dr Thomas Mueller, MD,^32^ Dr Michael Neumaier, MD,^33^ Prof Michael Behnes, MD,^34^ Prof Ibrahim Akin, MD,^34^ Prof Michele Bombelli, MD,^35^ Prof Guido Grassi, MD,^35^ Prof Olivier Hanon, MD PhD,^36^ Dr Jean-Sébastien Vidal, MD PhD,^36^ Dr Peiman Nazerian, MD,^37^ Dr Giovanni Albano, MD,^37^ Prof Peter Cameron, MD,^38^ Dr Louisa Lam, PhD,^38,39^ Dr Philipp Bahrmann, MD,^40^ Prof Ben Freedman, MB PhD,^41^ Dr Tommy Chung, PhD,^42^ Dr Sean P Collins, MD,^43^ Prof Christopher John Lindsell, PhD.^44^

**Writing group:** Dr Dimitrios Doudesis, PhD,^1,2^ Dr Kuan Ken Lee, MD,^1^ Dr Alan Japp, MD,^1^ Prof Humberto Villacorta, MD PhD,^14^ Prof A Mark Richards, MD,^29,45^ Prof John JV McMurray, MD,^46^ Prof Christian Mueller, MD,^8^ Prof James L Januzzi, MD,^18,19^ Prof Nicholas L Mills, MD.^1,2^

**Affiliations:**

^1^ British Heart Foundation (BHF) Centre for Cardiovascular Science, University of Edinburgh, Edinburgh, UK.

^2^ Usher Institute, University of Edinburgh, Edinburgh, UK.

^3^ London School of Hygiene and Tropical Medicine, London, UK.

^4^ Department of Emergency Medicine, Stony Brook University, New York, USA.

^5^ Department of Emergency Medicine, Thomas Jefferson University, Philadelphia, USA.

^6^ Department of Biochemistry, Cochin Hospital, Assistance Publique-Hopitaux de Paris, Paris, France.

^7^ Department of Emergency Medicine, Princess Grace Hospital Center, Monaco, Principalty of Monaco.

^8^ Cardiovascular Research Institute of Basel, Department of Cardiology, University Hospital Basel, Basel, Switzerland.

^9^ Department of Internal Medicine, University Hospital Basel, University of Basel, Switzerland.

^10^ Liverpool Heart and Chest Hospital, Liverpool, United Kingdom.

^11^ Division of Cardiology, University of Maryland School of Medicine, Baltimore, USA.

^12^ Division of Nephrology, University of Maryland School of Medicine, Baltimore, USA.

^13^ University of Toronto, St Michael’s Hospital, Toronto, Ontario, Canada.

^14^ Cardiology Division, Fluminense Federal University, Niteroi, Rio de Janeiro State, Brazil.

^15^ Heart Institute, Hospital Universitari Germans Trias i Pujol, Badalona, CIBERCV, Spain.

^16^ Department of Cardiology, Radboud University Medical Center, Nijmegen, The Netherlands.

^17^ University of Amsterdam, Amsterdam, The Netherlands.

^18^ Harvard Medical School, Boston, Massachusetts, USA.

^19^ Division of Cardiology, Massachusetts General Hospital, Boston, Massachusetts, USA.

^20^ B·R·A·H·M·S, Thermo Fisher Scientific, Hennigsdorf, Germany.

^21^ Department of Emergency and Acute Medicine with Chest Pain Units, Charité – Universitätsmedizin Berlin, Campus Mitte and Virchow, Berlin, Germany.

^22^ Cochin Hospital, Paris, France.

^23^ Cardiology Department, AP-HP, Paris-Saclay University, Paris, France.

^24^ Department of Internal Medicine, Radboud University Medical Center, Nijmegen, The Netherlands.

^25^ Department of Internal Medicine, Division of Pharmacology and Vascular Medicine, Erasmus Medical Center, Rotterdam, The Netherlands.

^26^ Division of Cardiology, The Jikei University Kashiwa Hospital, Kashiwa, Japan.

^27^ Institute of Clinical Physiology, National Research Council, Pisa, Italy.

^28^ Department of Clinical and Experimental Medicine, University of Pisa, Pisa, Italy.

^29^ Christchurch Heart Institute, University of Otago, Christchurch, New Zealand.

^30^ Emergency Medicine Department, National University Hospital, Singapore.

^31^ Department of Internal Medicine, Krankenhaus Bad Ischl, Bad Ischl, Austria.

^32^ Department of Laboratory Medicine, Hospital Voecklabruck, Austria.

^33^ Institute for Clinical Chemistry, University Medical Centre Mannheim, Faculty of Medicine Mannheim, University of Heidelberg, Mannheim, Germany.

^34^ First Department of Medicine, University Medical Centre Mannheim, Faculty of Medicine Mannheim, University of Heidelberg, Mannheim, Germany.

^35^ Clinica Medica, University of Milano – Bicocca, Monza, Italy.

^36^ Department of Geriatrics, Broca Hospital, Assistance Publique-Hôpitaux de Paris, Paris, France.

^37^ Department of Emergency Medicine, Azienda Ospedaliero-Universitaria Careggi, Florence, Italy.

^38^ Public Health and Preventive Medicine, Monash University, Australia.

^39^ Faculty of Health Science, Australian Catholic University, Australia.

^40^ Institute for Biomedicine of Aging, Friedrich-Alexander-University, Nuremberg, Germany.

^41^ Heart Research Institute, University of Sydney, Sydney, Australia.

^42^ Concord Repatriation General Hospital, NSW, Australia.

^43^ Department of Emergency Medicine, Vanderbilt University Medical Center, Nashville, Tennessee, USA.

^44^ Department of Biostatistics, Vanderbilt University Medical Center, Nashville, Tennessee, USA.

^45^ Cardiovascular Research Institute, National University Heart Centre Singapore, Singapore.

^46^ BHF Cardiovascular Research Centre, University of Glasgow, Glasgow, UK.

# **II. Supplementary Text**

## **Supplementary Text 1. Literature Search Strategy.**

**Embase**

1. *heart failure/ or acute heart failure/ or *cardiogenic shock/ or *diastolic dysfunction/ or *forward heart failure/ or *high output heart failure/ or *systolic dysfunction/
2. *Congestive Cardiomyopathy/ or exp *Congestive Heart Failure/
3. exp *Heart Ventricle Failure/
4. ((heart or cardiac or myocardial) adj2 (failure or decompensation)).ti.
5. ((congestive or acute or decompensat$) adj2 " heart failure").ti,ab.
6. ((dilated or congestive) adj2 cardiomyopath$).ti.
7. "cardiogenic shock".ti.
8. ((ventricular or ventricle$) adj2 (failure or insufficien$ or dysfunction$)).ti.
9. (("left ventricular" or "left ventricle") adj2 (failure or insufficien$ or dysfunction$)).ti,ab.
10. lvsd.ti,ab.
11. or/1-10
12. letter.pt. or letter/
13. note.pt.
14. editorial.pt.
15. case report/ or case study/
16. (letter or comment*).ti.
17. or/12-16
18. randomized controlled trial/ or random*.ti,ab.
19. 17 not 18
20. animal/ not human/
21. nonhuman/
22. exp Animal Experiment/
23. exp Experimental Animal/
24. animal model/
25. exp Rodent/
26. (rat or rats or mouse or mice).ti.
27. or/19-26
28. 11 not 27
29. limit 28 to english language
30. exp *natriuretic factor/
31. (natriuretic adj2 peptide$).ti,ab.
32. (natriuretic adj2 factor*).ti,ab.
33. (BNP or ANP or pro-BNP or pro-ANP or pro BNP or pro ANP).ti,ab.
34. or/30-33
35. 29 and 33

**Medline**

1. exp Heart Failure/
2. Cardiomyopathy, Dilated/
3. Shock, Cardiogenic/
4. exp Ventricular Dysfunction/
5. Cardiac Output, Low/
6. ((heart or cardiac or myocardial) adj2 (failure or decompensation)).ti.
7. ((congestive or acute or decompensat$) adj2 " heart failure").ti,ab.
8. ((dilated or congestive) adj2 cardiomyopath$).ti.
9. "cardiogenic shock".ti.
10. ((ventricular or ventricle$) adj2 (failure or insufficien$ or dysfunction$)).ti.
11. (("left ventricular" or "left ventricle") adj2 (failure or insufficien$ or dysfunction$)).ti,ab.
12. lvsd.ti,ab.
13. or/1-12
14. letter/
15. editorial/
16. news/
17. exp historical article/
18. Anecdotes as Topic/
19. comment/
20. case report/
21. (letter or comment*).ti.
22. or/14-21
23. randomized controlled trial/ or random*.ti,ab.
24. 22 not 23
25. animals/ not humans/
26. exp Animals, Laboratory/
27. exp Animal Experimentation/
28. exp Models, Animal/
29. exp Rodentia/
30. (rat or rats or mouse or mice).ti.
31. or/24-30
32. 13 not 31
33. limit 32 to english language
34. exp *Natriuretic Peptides/
35. (natriuretic adj2 peptide$).ti,ab.
36. (natriuretic adj2 factor*).ti,ab.
37. (BNP or ANP or pro-BNP or pro-ANP or pro BNP or pro ANP).ti,ab.
38. 34 or 35 or 36 or 37
39. 33 and 38

**Cochrane central register of controlled trials**

#1 MeSH descriptor: [Heart Failure] explode all trees

#2 MeSH descriptor: [Cardiomyopathy, Dilated] explode all trees

#3 MeSH descriptor: [Shock, Cardiogenic] explode all trees

#4 MeSH descriptor: [Ventricular Dysfunction] explode all trees

#5 MeSH descriptor: [Cardiac Output, Low] explode all trees

#6 (heart or cardiac or myocardial) near/2 (failure or decompensation):ti

#7 ((congestive or acute or decompensat*) near/2 "heart failure"):ti,ab

#8 (dilated or congestive) near/2 cardiomyopath*:ti

#9 cardiogenic shock:ti

#10 (ventricular or ventricle*) near/2 (failure or insufficien* or dysfunction*):ti

#11 (("left ventricle" or "left ventricular") near/2 (failure or insufficienc* or dysfunction*)):ti,ab

#12 lvsd:ti,ab

#13 MeSH descriptor: [Pulmonary Edema] this term only cardiogenic near/2 ("pulmonary edema" or "pulmonary oedema" or "lung edema" or "lung

#14 oedema"):ti,ab

#15 #1 or #2 or #3 or #4 or #5 or #6 or #7 or #8 or #9 or #10 or #11 or #12 or #13 or #14

#16 MeSH descriptor: [Natriuretic Peptides] explode all trees

#17 (natriuretic near/2 peptide*):ti,ab

#18 (natriuretic near/2 factor*):ti,ab

#19 (BNP or ANP or pro-BNP or pro-ANP or pro BNP or pro ANP):ti,ab

#20 #16 or #17 or #18 or #19

#21 #15 and #20

## **Supplementary Text 2. Statistical models for the development of the CoDE-HF score**

**S2.1 Extreme gradient boosting model**

XGBoost is a supervised machine learning technique initially proposed by Chen and Guestrin.(1) In brief, gradient boosting employs an ensemble technique to iteratively improve model accuracy for regression and classification problems. This ensemble-based algorithm is achieved by creating sequential models, using decision trees as learners where subsequent models attempt to correct errors of the preceding models.(2,3) In the boosting method, individuals that were misclassified by the previous model are assigned a higher weight to increase their chance of being selected in subsequent models. Each model is subsequently fitted in a step-wise fashion to minimise loss function such as absolute error or squared error (the amount predicted values differ from the true values). XGBoost refers to the re-engineering of gradient boosting to significantly improve the speed of the algorithm by pushing the limits of computational resources. The output of the XGBoost model is a probability that is computed by performing an inverse-logit transformation of the sum of the weights of the terminal nodes of the trained model.

The mathematical formula for the gradient boosting model can be described as:

|  | $\hat{y}_{i}= \sum_{k=1}^{K} f_{k} \left( x_{i} \right), f_{k}\in F$ | (1) |
| --- | --- | --- |

where f is an function that map each variable vector *x_i_* (*x_i_* = { *x_i_*, *x_2_*, …, *x_n_* }, *i* = 1, 2, N) to the outcome *y_i_*, *K* is the number of Classification and Regression Trees (CART) and *F* is the space of function containing all CART.^5^

XGBoost optimises an objective function of the form:

|  | $Obj= \sum_{i=1}^{N} l\left( y_{i}, \hat{y}_{i} \right)+ \sum_{k=1}^{K} {\Omega(f}_{k})$ | (2) |
| --- | --- | --- |

Where the first term is a loss function, *l*, which evaluates how well the model fits the data by measuring the difference between the prediction *ŷ_i_* and the outcome *y_i_*. The second term, the regularization term, is used by XGBoost to avoid overfitting by penalizing the complexity of the model. Furthermore, to improve and fully leverage the advantages of XGBoost we tuned the hyper-parameters of the algorithm defined below through a grid search strategy using 10-fold cross-validation.

The hyper-parameter values for the model in patients without prior heart failure for the BNP and MR-proANP, respectively, were: the number of iterations (trees) was set to 216 and 61, the learning rate (shrinkage parameter applied to each tree in the expansion) was set to 0.03 and 0.1, the interaction depth (maximum depth of each tree, expresses the highest level of variable interactions allowed) was set to 3 and 6, the minimum number of observations in the terminal nodes was set to 3.9 and 4.7, the fraction of the training set observations randomly selected for each subsequent tree was set to 0.71 and 0.68 and the fraction of variables randomly sampled for each tree was set to 0.57 and 0.53.

The hyper-parameter values for the model in patients with prior heart failure for the BNP and MR-proANP, respectively, were: the number of iterations (trees) was set to 235 and 135, the learning rate (shrinkage parameter applied to each tree in the expansion) was set to 0.06 and 0.05, the interaction depth (maximum depth of each tree, expresses the highest level of variable interactions allowed) was set to 3 and 6, the minimum number of observations in the terminal nodes was set to 3.3 and 3.5, the fraction of the training set observations randomly selected for each subsequent tree was set to 0.99 and 0.95 and the fraction of variables randomly sampled for each tree was set to 0.61 and 0.92.

The algorithm was developed using the R package ‘xgboost’

(<https://cran.r-project.org/web/packages/xgboost/>).

**S2.1.1 Relative feature importance plots**

a) Relative feature importance plot for the model developed for patients without prior heart failure for BNP.


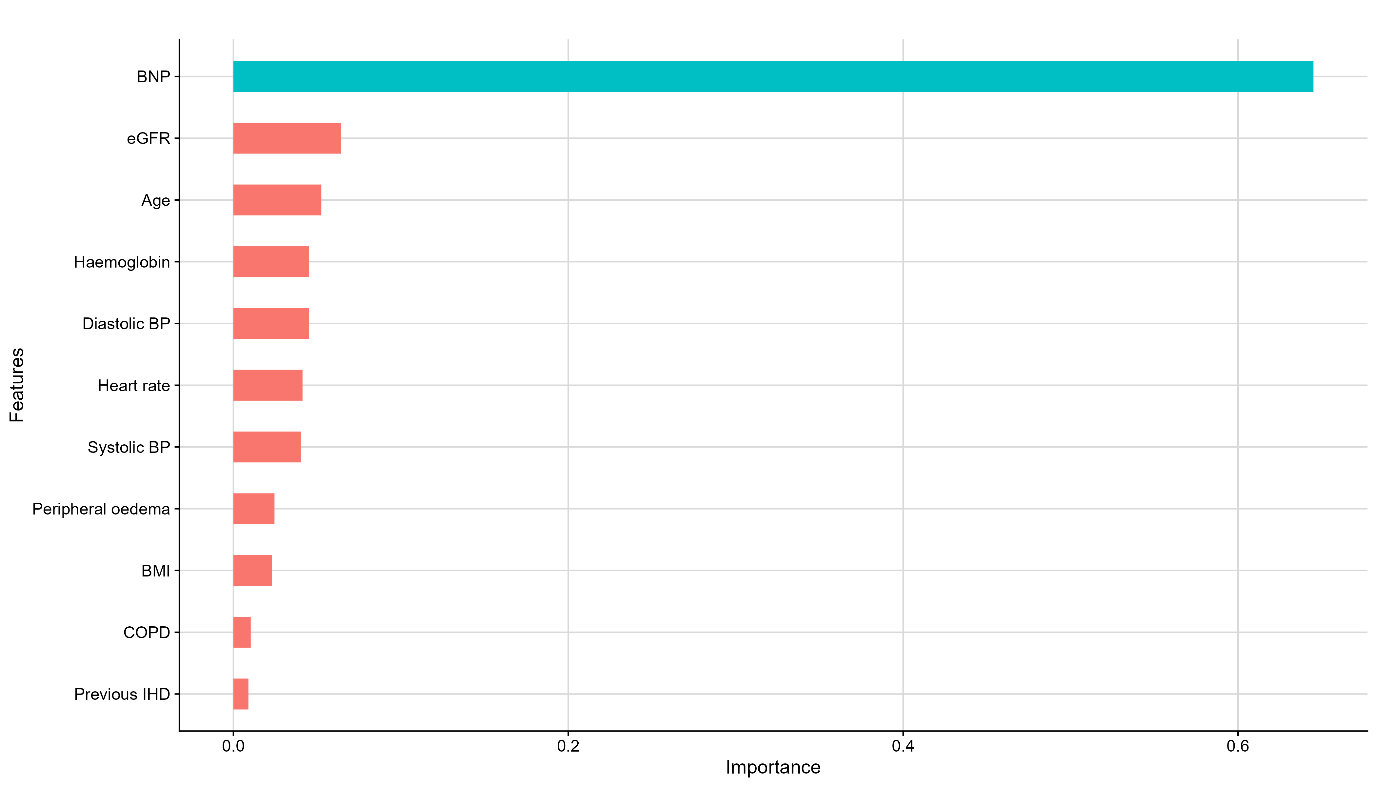
b) Relative feature importance plot for the model developed for patients with prior heart failure for BNP.


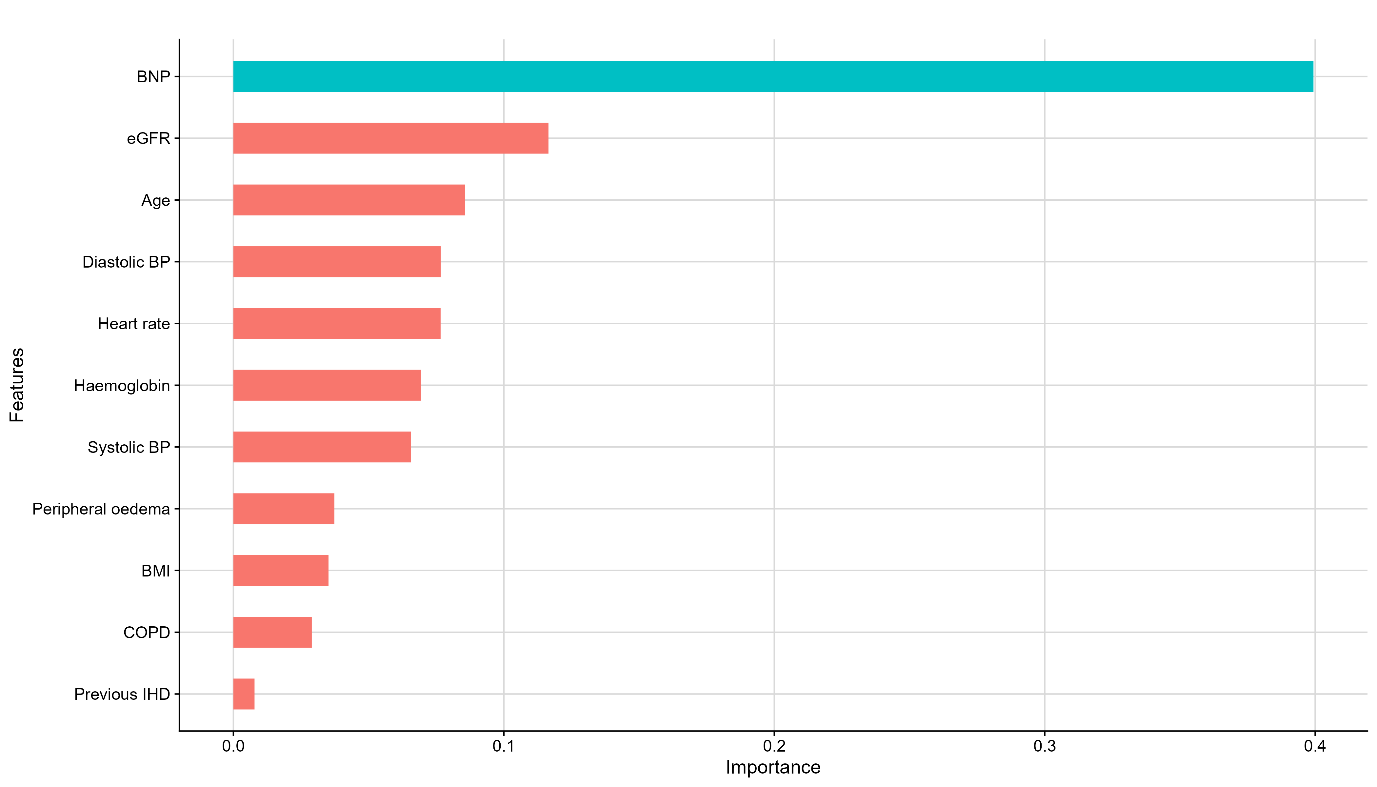


c) Relative feature importance plot for the model developed for patients without prior heart failure for MR-proANP.


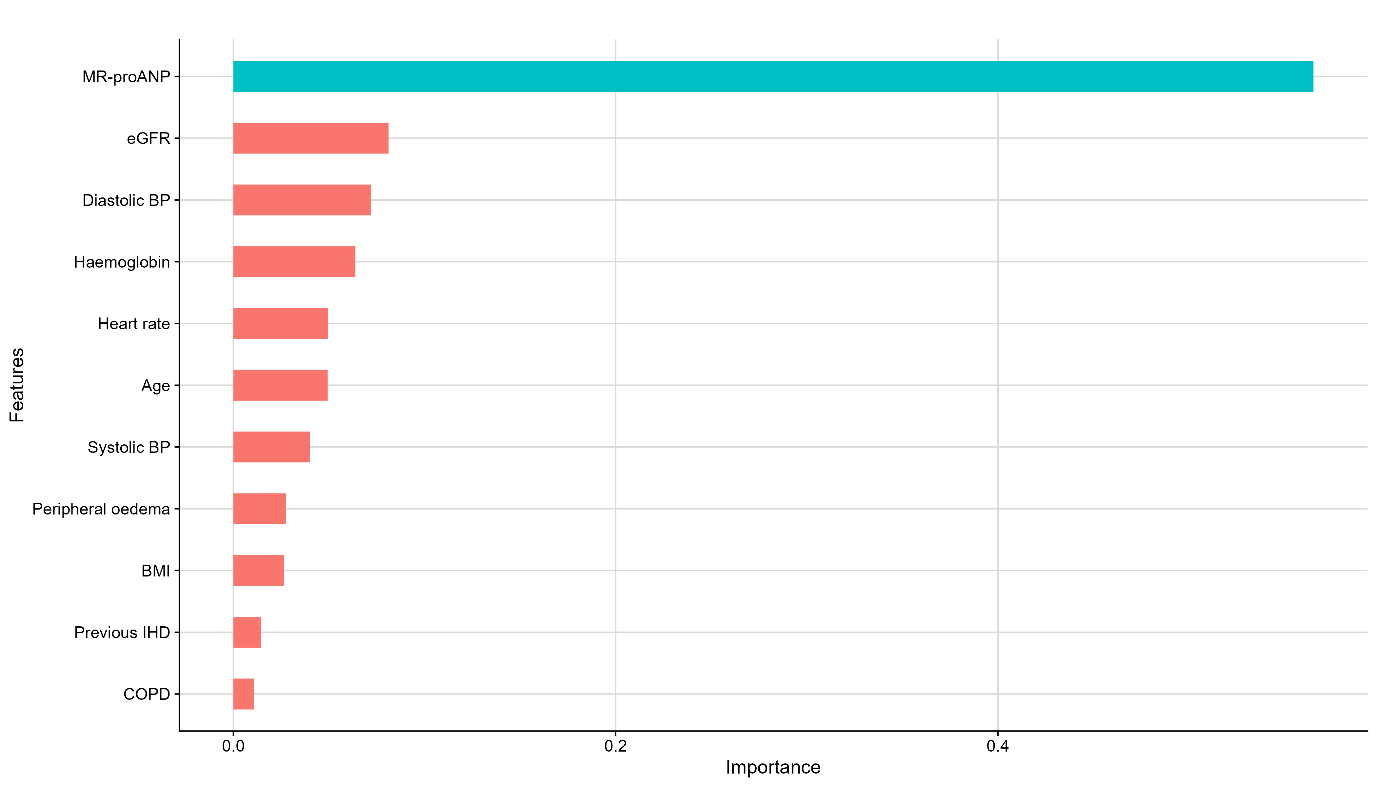
d) Relative feature importance plot for the model developed for patients with prior heart failure for MR-proANP.


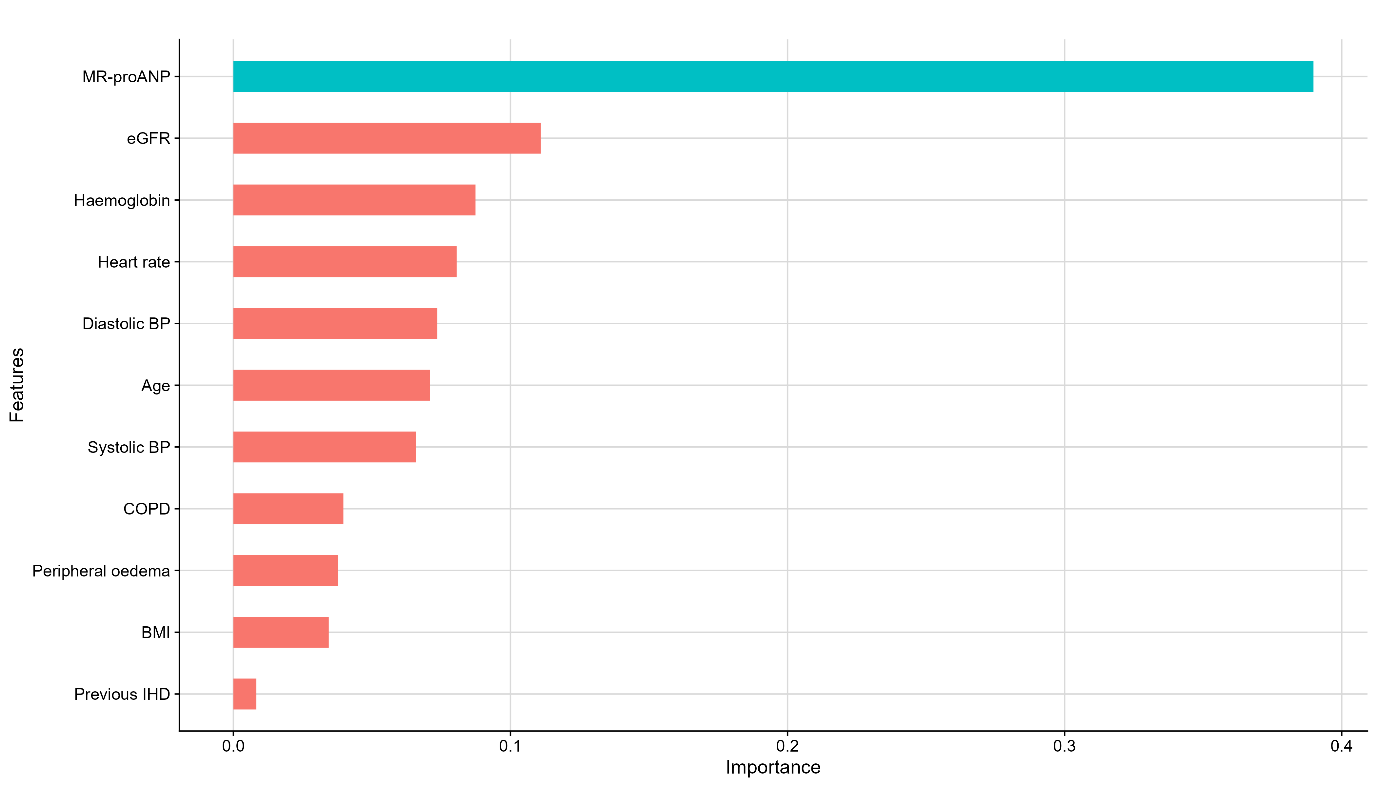


# **III. Supplementary Tables**

## **Supplementary Table 1. Characteristics of each included study.**

| Author, year | Study design | Cohort size | Setting | BNP assay | MR-proANP assay | Diagnostic adjudication for acute heart failure |
| --- | --- | --- | --- | --- | --- | --- |
| Chenevier-Gobeaux et al, 2005 | Prospective cohort | 380 | Emergency department | Triage Biosite | B·R·A·H·M·S | Independent adjudication by two ED clinicians on the basis of clinical examination, medical history, ECG, chest X-ray and blood analysis (including plasma creatinine). |
| Chung et al, 2006 | Prospective cohort | 143 | Emergency department | Triage Biosite |  | In all patients, an independent cardiologist who specialized in heart failure provided the reference diagnosis of acute heart failure as the cause of dyspnea. Diagnostic criteria used by the independent cardiologist for determination of cause of dyspnea included:  (1) presence or absence of clinical symptoms and signs of acute heart failure; (2) investigations including blood tests, chest radio- graphs, respiratory function test, and transthoracic echocardiography, (3) clinical response to heart failure therapy such as diuretics, and (4) final diagnosis of attending cardiologist/physician. The independent cardiologist was not blinded to the BNP level. |
| Collins et al, 2006 | Prospective cohort | 439 | Emergency department | Triage Biosite |  | Adjudication by two senior cardiology fellows who reviewed the entire medical record blinded to BNP values with discordant diagnoses adjudicated by the principal investigator. |
| Coste et al, 2006 | Prospective cohort | 699 | Emergency department | Triage Biosite |  | Diagnosis consensually established by 2 independent cardiologists who used all usual techniques (except BNP results) to rule in or rule out the diagnosis of heart failure, as recommended by the European Society of Cardiology guidelines. |
| deFilippi et al, 2007 | Prospective cohort | 831 | Emergency department | Triage Biosite |  | Adjudication by cardiologist who reviewed case report forms blinded to natriuretic peptide results. Subset of 50 random cases adjudicated by second cardiologist, demonstrating good agreement. |
| Ibrahim et al, 2017 | Prospective cohort | 1106 | Emergency department |  | B·R·A·H·M·S | Independent adjudication by an ED specialist and a cardiologist. They were blinded to NT-proBNP measurements but could access medical records, case report forms, and other test results including cardiac imaging as available. |
| Lokuge et al, 2010 | Prospective cohort | 612 | Emergency department | AbbottnAxSYM |  | The final diagnosis of HF was made by 1 emergency physician and 1 cardiologist who independently reviewed all available information, including case notes, blood tests, ECG and chest x-ray reports, response to treatment, echocardiogram, and pulmonary function test results but blinded to the BNP result. The reviewers diagnosed acute heart failure based on the European Society of Cardiology diagnostic criteria. |
| Maisel et al, 2002 | Prospective cohort | 1445 | Emergency department | Triage Biosite |  | Independent adjudication by two cardiologists who reviewed all medical records but blinded to BNP level. |
| Maisel et al, 2010 | Prospective cohort | 1623 | Emergency department |  | B·R·A·H·M·S | Independent adjudication by two cardiologists who reviewed all medical records. |
| Mueller et al, 2005 | Prospective cohort | 251 | Emergency department | Abbott AxSYM | B·R·A·H·M·S | Retrospective review of all medical records by a clinician based on Framingham criterion |
| Nakata et al, 2015 | Retrospective cohort | 269 | Emergency department | E Test TOSOH II |  | Diagnosis was confirmed retrospectively after discharge based on the Framingham criteria by one of the investigators who was independent of the emergency department and was blinded to the plasma BNP levels. |
| Plichart et al, 2017 | Prospective cohort | 383 | Acute geriatric ward | Triage Biosite |  | Diagnosis adjudicated by a cardiologist based on all medical records in accordance to the European Society of Cardiology guidelines but blinded to BNP levels. |
| Villacorta et al, 2002 | Prospective cohort | 70 | Emergency department | Triage Biosite |  | Diagnosis was adjudicated by a cardiologist who reviewed all pateints’ medical data but blinded to BNP values. |
| Wussler et al, 2019 | Prospective cohort | 2053 | Emergency department | Abbott AxSYM or Architect | B·R·A·H·M·S | Adjudicated by 2 independent cardiologist-internists who had access to all patients' medical records, including clinical history, physical examination, 12-lead electrocardiograms, laboratory findings, chest radiographs, echocardiograms, lung function test results, computed tomography scans, and response to therapy, as well as autopsy data for patients who died in the hospital. |

Abbreviations: ESC= European society of cardiology; ED= emergency department; ECG= electrocardiogram.

## **Supplementary Table 2. Baseline characteristics of patients with BNP measurements within each study.**

|  | Overall | Chenevier-Gobeaux et al | Chung et al | Collins et al | Coste et al | De Filippi et al | Lokuge et al |
| --- | --- | --- | --- | --- | --- | --- | --- |
| Number of participants | 8493 | 380 | 143 | 330 | 699 | 831 | 299 |
| Men | 4559 (53.7) | 189 (49.7) | 63 (44.1) | 154 (46.7) | 428 (61.2) | 380 (45.7) | 161 (53.8) |
| Age, years |  |  |  |  |  |  |  |
| < 50 | 1126 (13.3) | 5 (1.3) | 3 (2.1) | 60 (20.8) | 52 (7.4) | 125 (15.0) | 12 (4.0) |
| 50-75 | 3639 (43.1) | 121 (31.8) | 41 (28.7) | 144 (49.8) | 278 (39.8) | 443 (53.3) | 125 (41.8) |
| >75 | 3687 (43.6) | 254 (66.8) | 99 (69.2) | 85 (29.4) | 369 (52.8) | 263 (31.6) | 162 (54.2) |
| Ethnicity |  |  |  |  |  |  |  |
| Black | 964 (27.7) | NR | NR | 167 (50.6) | NR | 318 (38.3) | NR |
| Caucasian | 2282 (65.5) | NR | NR | 0 (0.0) | NR | 499 (60.0) | NR |
| Other | 237 (6.8) | NR | NR | 163 (49.4) | NR | 14 (1.7) | NR |
| Past medical history |  |  |  |  |  |  |  |
| Prior Heart failure | 2943 (36.3) | 128 (33.8) | 80 (55.9) | 198 (63.9) | 174 (24.9) | 287 (36.1) | 123 (41.1) |
| Ischemic heart disease | 2632 (36.4) | 124 (32.6) | 68 (47.6) | 132 (49.6) | NR | 263 (33.1) | 129 (43.1) |
| Diabetes Mellitus | 1756 (26.5) | NR | 38 (26.6) | NR | NR | 305 (38.2) | 62 (20.7) |
| Hypertension | 4167 (62.7) | 153 (40.3) | 77 (53.8) | 234 (73.1) | NR | NR | 167 (55.9) |
| Hyperlipidemia | 1350 (37.5) | NR | NR | NR | NR | NR | NR |
| Current or ex-smoker | 864 (31.7) | NR | 55 (38.5) | NR | NR | NR | 182 (60.9) |
| Asthma | 372 (19.0) | NR | NR | NR | NR | NR | 52 (17.4) |
| COPD | 2077 (34.4) | 127 (33.4) | NR | NR | NR | NR | 134 (44.8) |
| Atrial fibrillation | 1380 (21.5) | NR | 41 (28.7) | NR | NR | 175 (22.0) | 44 (14.7) |
| Chronic Kidney Disease | 931 (22.2) | NR | NR | NR | NR | NR | 30 (10.0) |
| Body mass index, kg/m^2^ |  |  |  |  |  |  |  |
| <25 | 2503 (40.6) | NR | NR | 100 (31.7) | NR | 195 (31.5) | NR |
| 25-30 | 1727 (28.0) | NR | NR | 61 (19.4) | NR | 172 (27.8) | NR |
| ≥30 | 1928 (31.3) | NR | NR | 154 (48.9) | NR | 252 (40.7) | NR |
| Physiological parameters |  |  |  |  |  |  |  |
| Heart rate, beats per minute | 91.7 (23.7) | NR | NR | NR | 90.2 (19.7) | NR | 95.3 (23.4) |
| Systolic blood pressure, mmHg | 140.0 (27.9) | NR | NR | NR | 146.7 (32.1) | NR | 143.1 (30.1) |
| Diastolic blood pressure, mmHg | 79.7 (17.0) | NR | NR | NR | 83.1 (20.5) | NR | 73.6 (18.3) |
| Clinical hematology and biochemistry |  |  |  |  |  |  |  |
| Hemoglobin, g/dL | 13.1 (4.9) | NR | NR | NR | NR | NR | 13.4 (1.8) |
| eGFR, mL/min/1.73m^2^ | 65.9 (30.5) | 54.7 (19.9) | 56.2 (25.3) | 65.9 (29.9) | NR | 63.3 (34.0) | NR |
| BNP, pg/mL | 255.1 [60.0, 801.0] | 286.0 [85.0, 860.5] | 488.0 [118.0, 1140.0] | 278.0 [58.0, 786.8] | 450.0 [130.5, 1020.5] | 337.0 [88.5, 811.0] | 273.0 [83.5, 933.0] |
| Adjudicated diagnosis of heart failure | 4105 (48.3) | 115 (30.3) | 72 (50.3) | 165 (50.0) | 417 (59.7) | 437 (52.6) | 147 (49.1) |

|  | Maisel et al (1) | Maisel et al (2) | Mueller et al | Nakata et al | Plichart et al | Villacorta et al | Wussler et al |
| --- | --- | --- | --- | --- | --- | --- | --- |
| Number of participants | 1586 | 1638 | 251 | 269 | 378 | 70 | 1619 |
| Men | 883 (55.7) | 857 (52.3) | 234 (93.2) | 174 (64.7) | 128 (33.9) | 33 (47.1) | 875 (54.0) |
| Age, years |  |  |  |  |  |  |  |
| < 50 | 349 (22.0) | 336 (20.5) | 24 (9.6) | 16 (5.9) | 0 (0.0) | 10 (14.3) | 134 (8.3) |
| 50-75 | 771 (48.6) | 810 (49.5) | 115 (45.8) | 136 (50.6) | 0 (0.0) | 23 (32.9) | 621 (38.4) |
| >75 | 466 (29.4) | 492 (30.0) | 112 (44.6) | 117 (43.5) | 378 (100.0) | 37 (52.9) | 864 (53.4) |
| Ethnicity |  |  |  |  |  |  |  |
| Black | NR | 476 (29.3) | 0 (0.0) | NR | 0 (0.0) | 3 (4.3) | NR |
| Caucasian | NR | 1087 (67.0) | 251 (100.0) | NR | 378 (100.0) | 67 (95.7) | NR |
| Other | NR | 60 (3.7) | 0 (0.0) | NR | 0 (0.0) | 0 (0.0) | NR |
| Past medical history |  |  |  |  |  |  |  |
| Prior Heart failure | 527 (33.2) | 568 (35.6) | 75 (29.9) | NR | 216 (57.1) | 26 (37.1) | 541 (33.5) |
| Ischemic heart disease | 567 (39.3) | 501 (31.6) | 117 (46.6) | NR | 126 (33.3) | 30 (42.9) | 575 (35.6) |
| Diabetes Mellitus | 367 (25.4) | 461 (28.5) | 58 (23.1) | NR | 52 (13.8) | 22 (31.4) | 391 (24.2) |
| Hypertension | 879 (55.4) | 1078 (66.9) | 141 (56.2) | NR | 259 (68.5) | 36 (51.4) | 1143 (70.9) |
| Hyperlipidemia | NR | 569 (36.8) | NR | NR | 68 (18.0) | 18 (25.7) | 695 (43.3) |
| Current or ex-smoker | NR | 468 (29.4) | 46 (18.3) | NR | 77 (20.4) | 36 (53.7) | NR |
| Asthma | NR | 318 (20.0) | NR | NR | NR | 2 (2.9) | NR |
| COPD | 600 (41.4) | 470 (29.5) | 72 (28.7) | NR | 88 (23.3) | 31 (44.3) | 555 (34.4) |
| Atrial fibrillation | 256 (17.9) | 242 (16.8) | 83 (33.1) | NR | 134 (35.4) | 10 (14.3) | 395 (24.4) |
| Chronic Kidney Disease | NR | 244 (15.4) | 74 (29.5) | NR | 94 (24.9) | 6 (8.7) | 483 (29.9) |
| Body mass index, kg/m^2^ |  |  |  |  |  |  |  |
| <25 | 526 (38.4) | 490 (35.1) | 93 (37.1) | 204 (79.4) | 168 (60.0) | 20 (28.6) | 707 (44.2) |
| 25-30 | 366 (26.7) | 389 (27.9) | 100 (39.8) | 40 (15.6) | 69 (24.6) | 36 (51.4) | 494 (30.9) |
| ≥30 | 477 (34.8) | 517 (37.0) | 58 (23.1) | 13 (5.1) | 43 (15.4) | 14 (20.0) | 400 (25.0) |
| Physiological parameters |  |  |  |  |  |  |  |
| Heart rate, beats per minute | 92.1 (23.0) | 91.4 (22.8) | 93.8 (26.1) | 97.6 (26.9) | 86.4 (19.2) | 89.2 (18.0) | 93.0 (24.6) |
| Systolic blood pressure, mmHg | 140.9 (30.1) | 140.8 (28.7) | 140.1 (31.9) | 138.2 (29.4) | 129.6 (24.8) | 134.7 (29.5) | 138.6 (26.7) |
| Diastolic blood pressure, mmHg | 78.8 (18.3) | 80.8 (17.3) | 81.8 (16.3) | 79.4 (20.2) | 70.5 (13.4) | 80.9 (17.5) | 79.7 (17.7) |
| Clinical hematology and biochemistry |  |  |  |  |  |  |  |
| Hemoglobin, g/dL | 12.8 (2.2) | 13.7 (2.0) | 12.7 (2.4) | 11.7 (1.8) | 26.4 (37.4) | 13.2 (2.1) | 12.9 (2.2) |
| eGFR, mL/min/1.73m^2^ | 69.6 (27.5) | 94.0 (44.6) | 65.7 (23.9) | 39.5 (20.4) | 56.4 (21.6) | 65.2 (28.6) | 70.5 (30.4) |
| BNP, pg/mL | 156.2 [29.8, 680.9] | 295.7 [69.2, 911.2] | 233.8 [60.6, 623.9] | 291.0 [120.0, 670.8] | 320.0 [75.2, 1052.0] | 338.0 [77.0, 989.0] | 164.0 [36.0, 573.8] |
| Adjudicated diagnosis of heart failure | 744 (46.9) | 137 (54.6) | 131 (48.7) | 235 (62.2) | 36 (51.4) | 903 (55.8) | 566 (34.6) |

Presented as No. (%), mean (SD) or median [inter-quartile range]. Abbreviations: COPD= chronic obstructive pulmonary disease; eGFR= estimated glomerular filtration rate; NT-proBNP= N-terminal pro-B-type natriuretic peptide; CVD= cardiovascular disease; NR= not reported.

## **Supplementary Table 3. Baseline characteristics of patients with MR-proANP measurements within each study.**

|  | Overall | Ibrahim et al, 2017 | Maisel et al, 2010 | Mueller et al, 2005 | Wussler et al, 2019 |
| --- | --- | --- | --- | --- | --- |
| Number of participants | 3899 | 607 | 1635 | 251 | 1406 |
| Men | 2258 (57.9) | 397 (65.4) | 856 (52.4) | 234 (93.2) | 771 (54.8) |
| Age, years |  |  |  |  |  |
| < 50 | 680 (17.4) | 196 (32.3) | 335 (20.5) | 24 (9.6) | 125 (8.9) |
| 50-75 | 1826 (46.8) | 350 (57.7) | 807 (49.4) | 115 (45.8) | 554 (39.4) |
| >75 | 1393 (35.7) | 61 (10.0) | 493 (30.2) | 112 (44.6) | 727 (51.7) |
| Ethnicity |  |  |  |  |  |
| Black | 473 (19.1) | 0 (0.0) | 473 (29.2) | 0 (0.0) | NR |
| Caucasian | 1338 (54.0) | 0 (0.0) | 1087 (67.1) | 251 (100.0) | NR |
| Other | 667 (26.9) | 607 (100.0) | 60 (3.7) | 0 (0.0) | NR |
| Past medical history |  |  |  |  |  |
| Prior Heart failure | 1199 (31.2) | 88 (14.5) | 565 (35.5) | 75 (29.9) | 471 (33.7) |
| Ischemic heart disease | 1150 (30.0) | 52 (8.6) | 499 (31.5) | 117 (46.6) | 482 (34.4) |
| Diabetes Mellitus | 1047 (27.0) | 203 (33.4) | 460 (28.5) | 58 (23.1) | 326 (23.2) |
| Hypertension | 2529 (65.4) | 329 (54.2) | 1077 (66.9) | 141 (56.2) | 982 (70.1) |
| Hyperlipidemia | 1421 (40.2) | 274 (45.1) | 566 (36.7) | NR | 581 (42.1) |
| Current or ex-smoker | 672 (27.5) | 160 (26.5) | 466 (29.4) | 46 (18.3) | NR |
| Asthma | 488 (22.2) | 171 (28.2) | 317 (20.0) | NR | NR |
| COPD | 1060 (27.5) | 46 (7.6) | 469 (29.5) | 72 (28.7) | 473 (33.7) |
| Atrial fibrillation | 722 (19.8) | 54 (9.9) | 241 (16.7) | 83 (33.1) | 344 (24.5) |
| Chronic Kidney Disease | 793 (20.7) | 54 (8.9) | 243 (15.4) | 74 (29.5) | 422 (30.1) |
| Body mass index, kg/m^2^ |  |  |  |  |  |
| <25 | 1311 (40.1) | 111 (46.6) | 490 (35.2) | 93 (37.1) | 617 (44.5) |
| 25-30 | 973 (29.8) | 59 (24.8) | 388 (27.9) | 100 (39.8) | 426 (30.7) |
| ≥30 | 986 (30.2) | 68 (28.6) | 515 (37.0) | 58 (23.1) | 345 (24.9) |
| Physiological parameters |  |  |  |  |  |
| Heart rate, beats per minute | 92.3 (23.3) | 91.8 (21.4) | 91.4 (22.8) | 93.8 (26.1) | 93.2 (24.0) |
| Systolic blood pressure, mmHg | 139.6 (27.8) | 138.2 (26.5) | 140.8 (28.6) | 140.1 (31.9) | 138.7 (26.5) |
| Diastolic blood pressure, mmHg | 80.7 (17.2) | 80.7 (16.3) | 80.8 (17.3) | 81.8 (16.3) | 80.5 (17.5) |
| Clinical hematology and biochemistry |  |  |  |  |  |
| Hemoglobin, g/dL | 13.1 (2.1) | 13.4 (2.0) | 12.9 (2.2) | 13.7 (2.0) | 13.1 (2.1) |
| eGFR, mL/min/1.73m^2^ | 72.0 (32.0) | 82.4 (29.3) | 70.5 (30.3) | 94.0 (44.6) | 65.3 (29.2) |
| Mr-proANP, pmol/L | 191.0 [71.3, 385.0] | 84.9 [41.8, 257.1] | 174.5 [66.5, 369.4] | 212.0 [101.0, 412.0] | 243.5 [109.2, 439.8] |
| Adjudicated diagnosis of heart failure | 1611 (41.3) | 148 (24.4) | 564 (34.5) | 137 (54.6) | 762 (54.2) |

Presented as No. (%), mean (SD) or median [inter-quartile range]. Abbreviations: COPD= chronic obstructive pulmonary disease; eGFR= estimated glomerular filtration rate; NT-proBNP= N-terminal pro-B-type natriuretic peptide; CVD= cardiovascular disease; NR= not reported.

## **Supplementary Table 4. Baseline characteristics of study patients stratified by prior history of heart failure.**

|  | BNP | | | MR-proANP | | |
| --- | --- | --- | --- | --- | --- | --- |
|  | Overall | No prior history of acute heart failure | Prior history of acute heart failure | Overall | No prior history of acute heart failure | Prior history of acute heart failure |
| Number of participants | 8493 | 5175 | 2943 | 3899 | 2648 | 1199 |
| Men | 4559 (53.7) | 2657 (51.3) | 1675 (56.9) | 2258 (57.9) | 1474 (55.7) | 755 (63.0) |
| Age, years |  |  |  |  |  |  |
| <50 | 1126 (13.3) | 859 (16.7) | 231 (7.9) | 680 (17.4) | 592 (22.4) | 80 (6.7) |
| 50-75 | 3639 (43.1) | 2299 (44.6) | 1157 (39.6) | 1826 (46.8) | 1298 (49.0) | 507 (42.3) |
| >75 | 3687 (43.6) | 1998 (38.8) | 1534 (52.5) | 1393 (35.7) | 758 (28.6) | 612 (51.0) |
| Ethnicity |  |  |  |  |  |  |
| Black | 964 (27.7) | 545 (27.0) | 389 (28.5) | 473 (19.1) | 300 (17.5) | 167 (23.0) |
| Caucasian | 2282 (65.5) | 1367 (67.8) | 860 (62.9) | 1338 (54.0) | 852 (49.8) | 451 (62.2) |
| Other | 237 (6.8) | 105 (5.2) | 118 (8.6) | 667 (26.9) | 558 (32.6) | 107 (14.8) |
| Past medical history |  |  |  |  |  |  |
| Ischaemic heart disease | 2632 (36.4) | 1115 (24.7) | 1484 (56.0) | 1150 (30.0) | 445 (16.9) | 687 (58.6) |
| Diabetes mellitus | 1756 (26.5) | 912 (21.8) | 822 (34.4) | 1047 (27.0) | 592 (22.4) | 444 (37.2) |
| Hypertension | 4167 (62.7) | 2283 (55.3) | 1831 (74.2) | 2529 (65.4) | 1525 (57.9) | 965 (81.0) |
| Hyperlipidemia | 1350 (37.5) | 662 (29.3) | 671 (51.2) | 1421 (40.2) | 780 (32.2) | 621 (57.5) |
| Current or ex-smoker | 864 (31.7) | 559 (34.5) | 289 (27.0) | 672 (27.5) | 501 (29.5) | 155 (21.8) |
| Asthma | 372 (19.0) | 277 (22.5) | 87 (12.5) | 488 (22.2) | 397 (26.0) | 83 (13.1) |
| COPD | 2077 (34.4) | 1343 (34.8) | 716 (33.4) | 1060 (27.5) | 696 (26.4) | 345 (29.3) |
| Atrial fibrillation | 1380 (21.5) | 645 (16.1) | 718 (30.4) | 722 (19.8) | 345 (14.3) | 368 (31.4) |
| Chronic kidney disease | 931 (22.2) | 363 (13.7) | 560 (36.7) | 793 (20.7) | 338 (12.8) | 447 (38.0) |
| Body mass index, kg/m^2^ |  |  |  |  |  |  |
| <25 | 2503 (40.6) | 1453 (39.9) | 813 (37.4) | 1311 (40.1) | 917 (42.6) | 377 (35.2) |
| 25-29 | 1727 (28.0) | 1016 (27.9) | 645 (29.7) | 973 (29.8) | 597 (27.7) | 362 (33.8) |
| ≥30 | 1928 (31.3) | 1172 (32.2) | 713 (32.8) | 986 (30.2) | 640 (29.7) | 331 (30.9) |
| Physiological parameters |  |  |  |  |  |  |
| Heart rate, beats per minute | 92.1 (23.4) | 93.3 (22.9) | 89.6 (23.4) | 92.3 (23.3) | 93.5 (22.7) | 89.5 (24.2) |
| Systolic blood pressure, mmHg | 139.8 (28.9) | 141.5 (27.8) | 137.0 (30.3) | 139.6 (27.8) | 141.4 (26.7) | 135.2 (29.5) |
| Diastolic blood pressure, mmHg | 79.2 (18.0) | 80.2 (17.4) | 77.3 (18.5) | 80.7 (17.2) | 81.8 (16.8) | 78.2 (17.8) |
| Clinical haematology and biochemistry |  |  |  |  |  |  |
| Haemoglobin, g/dL | 13.1 (4.9) | 13.3 (5.0) | 12.7 (5.1) | 13.1 (2.1) | 13.3 (2.1) | 12.6 (2.1) |
| eGFR, mL/min/1.73 m^2^ | 65.9 (30.5) | 72.3 (31.1) | 55.4 (26.9) | 72.0 (32.0) | 79.3 (31.4) | 56.7 (27.5) |
| BNP, pg/mL | 255.1 [60.0, 801.0] | 131.0 [34.0, 513.0] | 615.0 [222.0, 1208.5] | - | - | - |
| MR-proANP, pmol/L | - | - | - | 191.0 [71.3, 385.0] | 114.0 [53.1, 265.2] | 386.6 [231.7, 604.7] |
| Adjudicated diagnosis of heart failure | 4105 (48.3) | 1724 (33.3) | 2219 (75.4) | 1611 (41.3) | 714 (27.0) | 884 (73.7) |

Presented as No. (%), mean (SD) or median [inter-quartile range].

Abbreviations: COPD= chronic obstructive pulmonary disease; eGFR= estimated glomerular filtration rate;

BNP= B-type natriuretic peptide; MR-proANP = Mid-regional pro-atrial natriuretic peptide.

**Supplementary Table 5. Diagnostic performance of different CoDE-HF scores for BNP.**

**a)** Patients without a prior history of heart failure

|  | **Threshold** | **True negative** | **False negative** | **True positive** | **False positive** | **NPV**  **(95% CI)** | **Sensitivity (95% CI)** | **Proportion ruled out** |
| --- | --- | --- | --- | --- | --- | --- | --- | --- |
| **Low probability CoDE-HF scores** | | | | | | | | |
| More conservative | 3.9 | 1190 | 12 | 1712 | 2261 | 99.1 (97.5-99.6) | 99.4 (98.5-99.8) | 23% |
| **Selected** | **5.4** | **1508** | **20** | **1704** | **1943** | **98.5 (97.1-99.3)** | **98.9 (98.0-99.3)** | **30%** |
| Less conservative | 13.1 | 2239 | 84 | 1640 | 1212 | 95.9 (93.5-97.4) | 95.0 (93.4-96.3) | 45% |

|  |  | **True negative** | **False negative** | **True positive** | **False positive** | **PPV**  **(95% CI)** | **Specificity (95% CI)** | **Proportion ruled in** |
| --- | --- | --- | --- | --- | --- | --- | --- | --- |
| **High probability CoDE-HF scores** | | | | | | | | |
| Less conservative | 33.0 | 2819 | 229 | 1495 | 632 | 70.0 (62.2-76.9) | 80.8 (75.7-85.0) | 41% |
| **Selected** | **58.0** | **3122** | **484** | **1240** | **329** | **78.6 (70.4-85.0)** | **90.2 (86.8-92.8)** | **30%** |
| More conservative | 61.9 | 3171 | 579 | 1145 | 280 | 80.1 (71.1-86.8) | 92.0 (88.8-94.3) | 28% |

**b)** Patients with a prior history of heart failure

|  |  | **True negative** | **False negative** | **True positive** | **False positive** | **PPV**  **(95% CI)** | **Specificity (95% CI)** | **Proportion ruled in** |
| --- | --- | --- | --- | --- | --- | --- | --- | --- |
| **High probability CoDE-HF scores** | | | | | | | | |
| Less conservative | 81.4 | 582 | 619 | 1600 | 142 | 91.6 (87.0-94.6) | 80.8 (74.1-86.1) | 59% |
| **Selected** | **90.7** | **664** | **1126** | **1093** | **60** | **94.9 (90.9-97.1)** | **92.6 (87.7-95.7)** | **39%** |
| More conservative | 94.2 | 704 | 1581 | 638 | 20 | 97.0 (95.3-98.0) | 97.5 (95.3-98.7) | 22% |

**Supplementary Table 6. Diagnostic performance of different CoDE-HF scores for MR-proANP.**

**a)** Patients without a prior history of heart failure

|  | **Threshold** | **True negative** | **False negative** | **True positive** | **False positive** | **NPV**  **(95% CI)** | **Sensitivity (95% CI)** | **Proportion ruled out** |
| --- | --- | --- | --- | --- | --- | --- | --- | --- |
| **Low probability CoDE-HF scores** | | | | | | | | |
| More conservative | 5.2 | 1101 | 11 | 703 | 833 | 99.0 (98.1-99.5) | 98.4 (96.2-99.4) | 42% |
| **Selected** | **8.1** | **1259** | **19** | **695** | **675** | **98.5 (97.7-99.0)** | **97.3 (95.5-98.4)** | **48%** |
| Less conservative | 12.0 | 1386 | 34 | 680 | 548 | 97.4 (94.7-98.7) | 95.2 (93.4-96.6) | 54% |

|  |  | **True negative** | **False negative** | **True positive** | **False positive** | **PPV**  **(95% CI)** | **Specificity (95% CI)** | **Proportion ruled in** |
| --- | --- | --- | --- | --- | --- | --- | --- | --- |
| **High probability CoDE-HF scores** | | | | | | | | |
| Less conservative | 33.5 | 1678 | 105 | 609 | 256 | 70.0 (65.6-74.1) | 86.1 (79.2-90.9) | 33% |
| **Selected** | **46.0** | **1755** | **166** | **548** | **179** | **75.1 (70.9-78.9)** | **90.4 (86.1-93.5)** | **28%** |
| More conservative | 65.8 | 1848 | 319 | 395 | 86 | 80.8 (74.8-85.7) | 95.1 (91.9-97.1) | 18% |

**b)** Patients with a prior history of heart failure

|  |  | **True negative** | **False negative** | **True positive** | **False positive** | **PPV**  **(95% CI)** | **Specificity (95% CI)** | **Proportion ruled in** |
| --- | --- | --- | --- | --- | --- | --- | --- | --- |
| **High probability CoDE-HF scores** | | | | | | | | |
| Less conservative | 86.7 | 268 | 276 | 608 | 47 | 91.7 (86.2-95.2) | 80.4 (67.4-89.1) | 55% |
| **Selected** | **91.7** | **290** | **425** | **459** | **25** | **94.2 (89.5-96.9)** | **90.1 (81.4-95.0)** | **40%** |
| More conservative | 94.7 | 302 | 589 | 295 | 13 | 95.8 (92.9-97.5) | 95.4 (90.5-97.9) | 26% |

# **IV. Supplementary Figures**

## **Supplementary Figure 1. Flow diagram of study participants.**


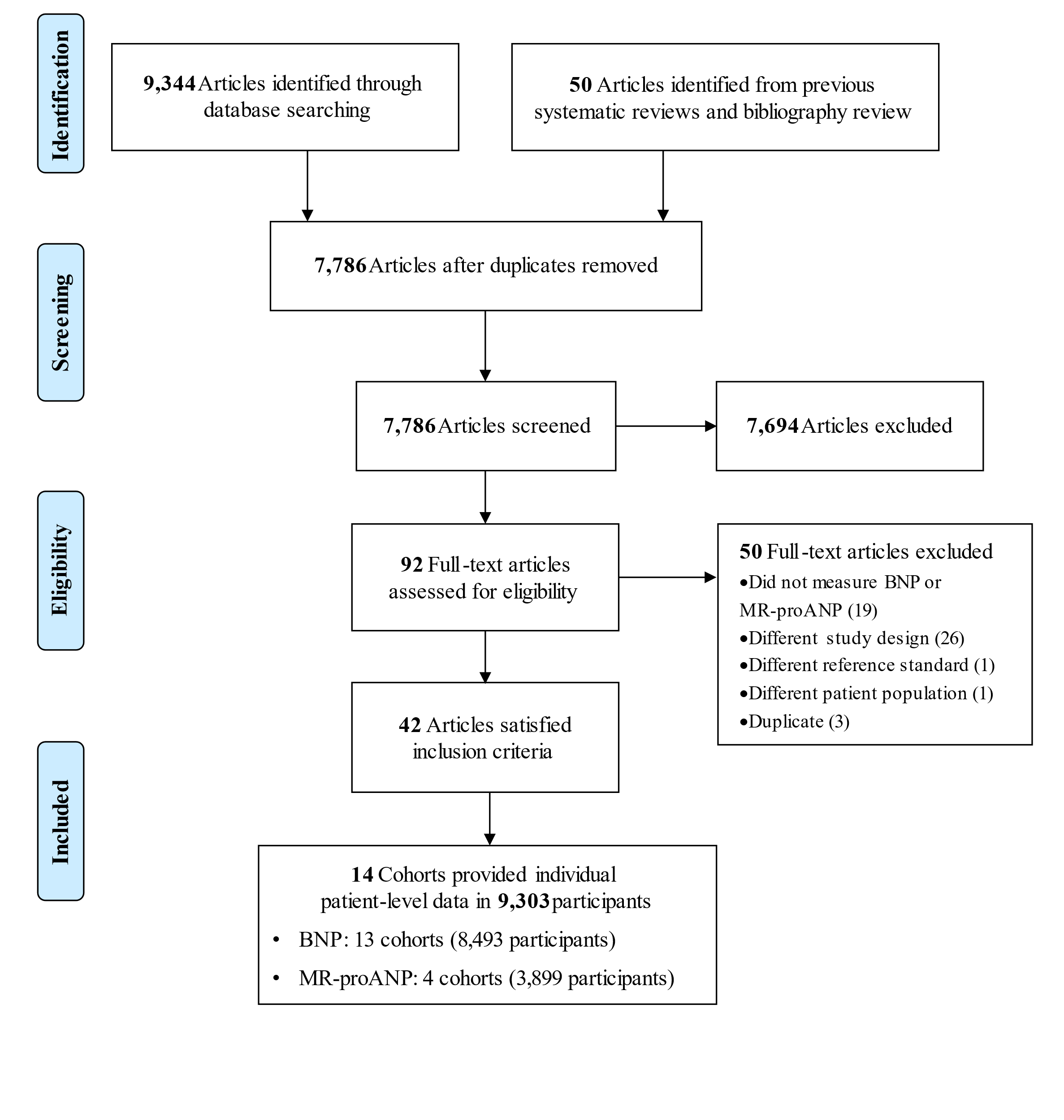


**Supplementary Figure 2.**

**a)** **Positive predictive value of guideline-recommended BNP thresholds across patient subgroups.**

**
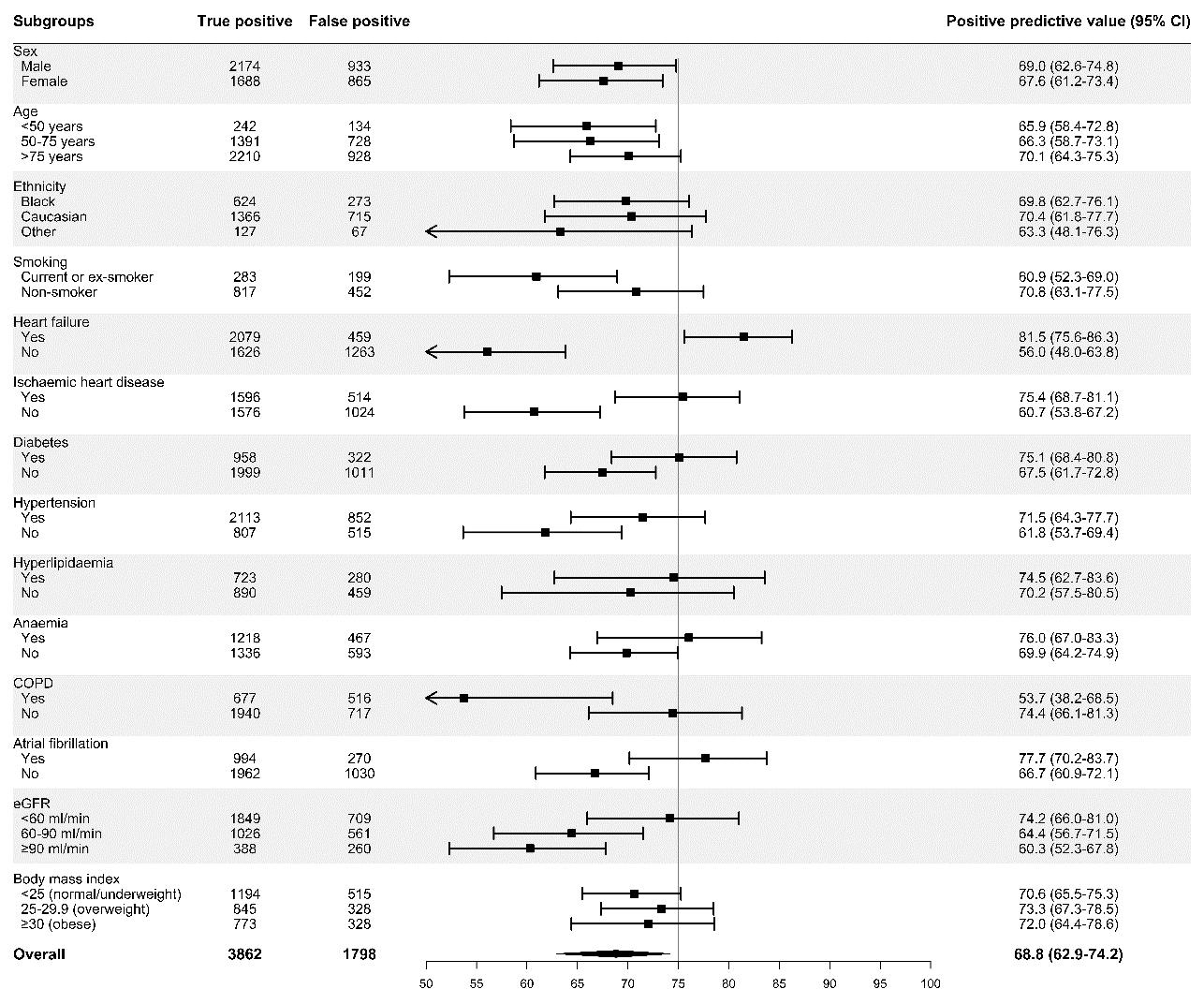
**

**b)** **Positive predictive value of guideline-recommended MR-proANP thresholds across patient subgroups.**


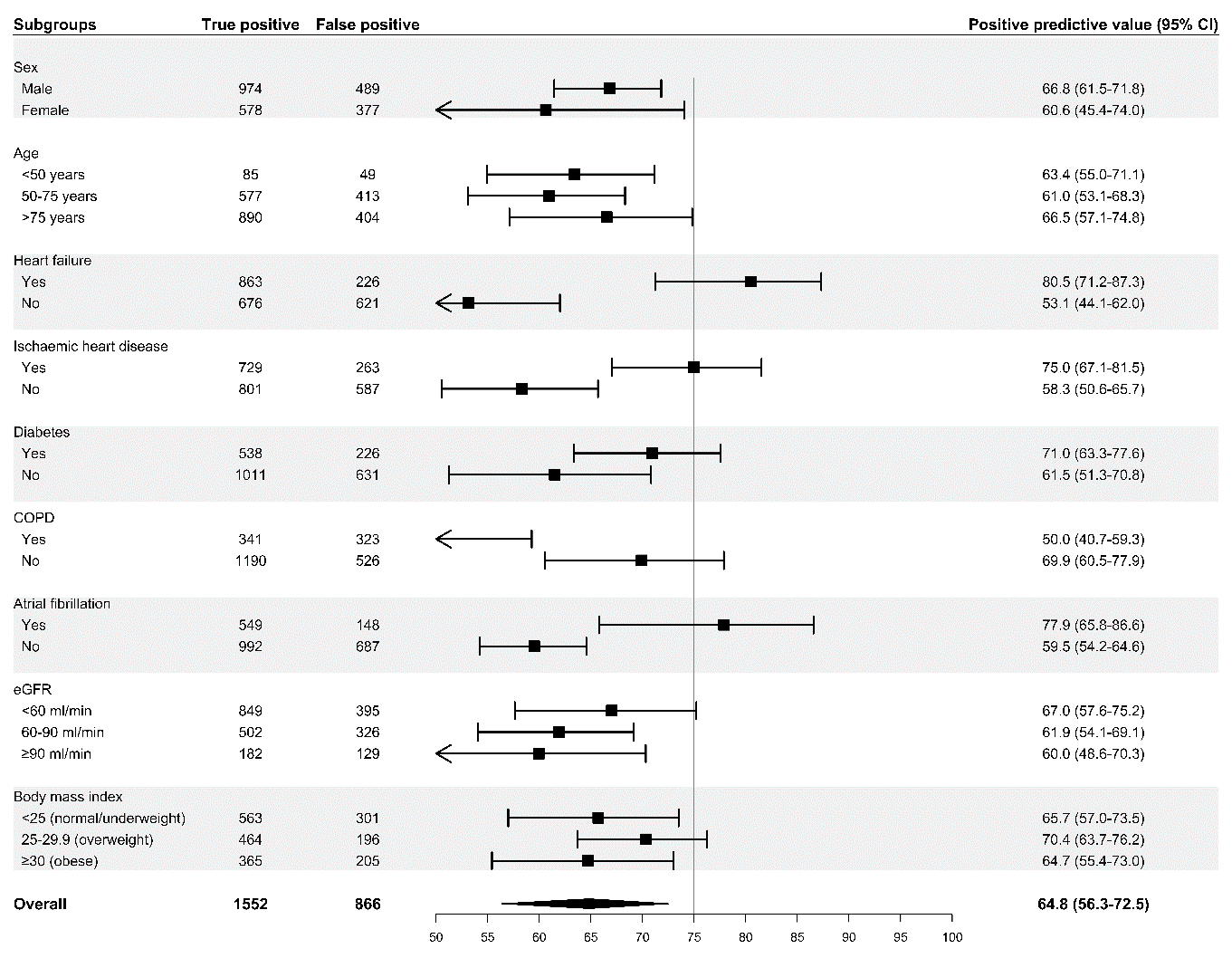


**Supplementary Figure 3. Negative predictive value of optimised MR-proANP threshold of 80 pmol/L across patient subgroups.**

**
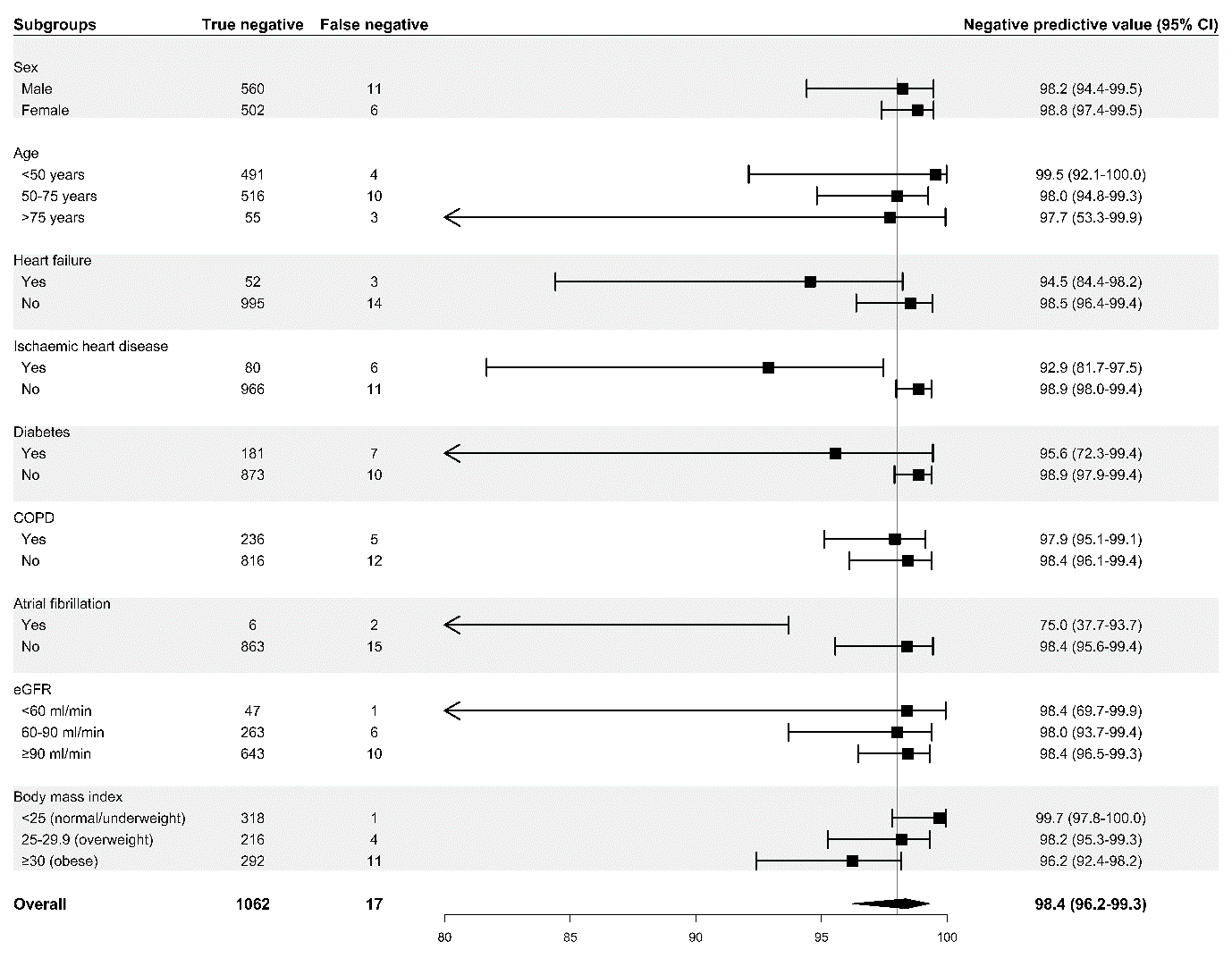
**

**Supplementary Figure 4. Discrimination of the CoDE-HF score with BNP. Receiver operator curve in patients**


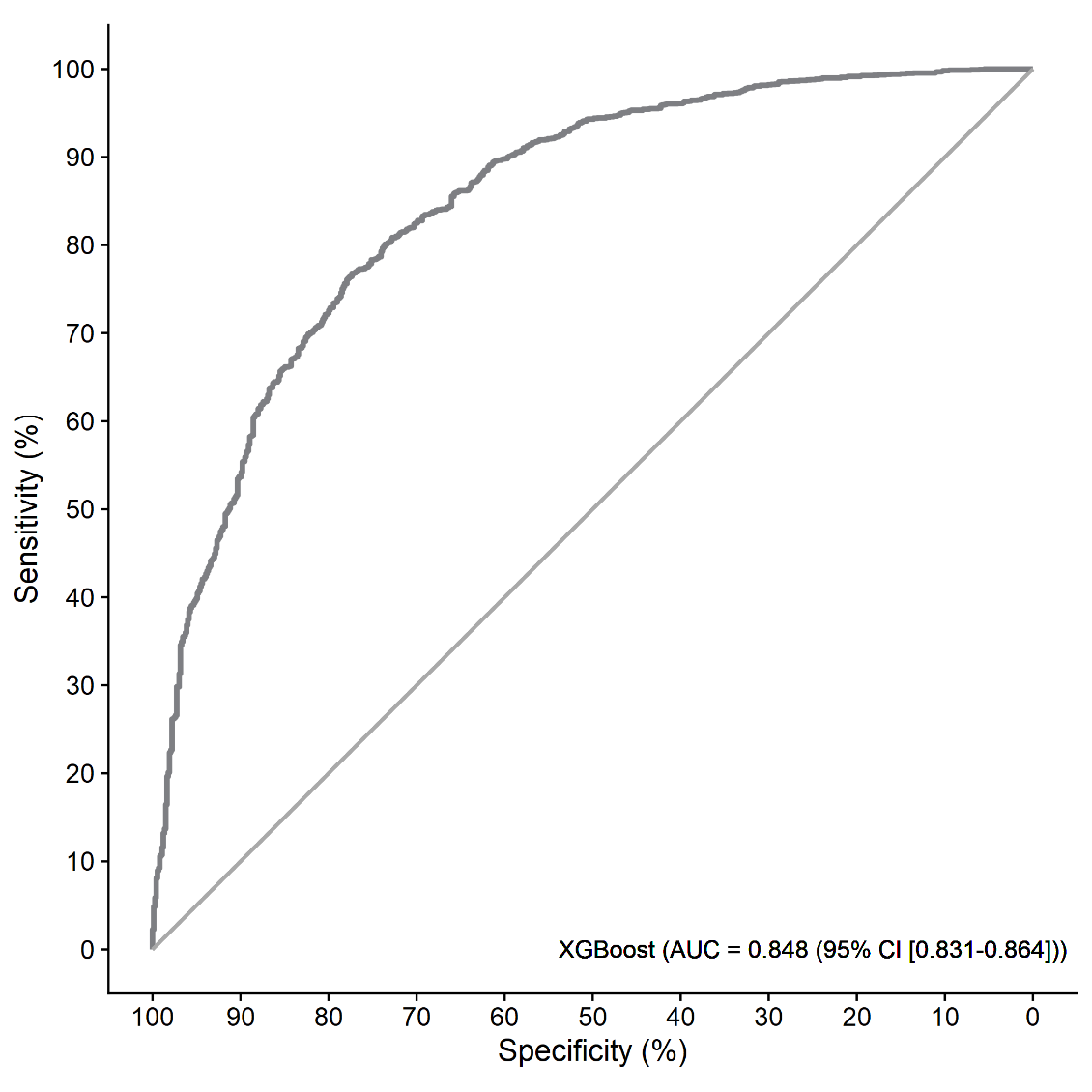

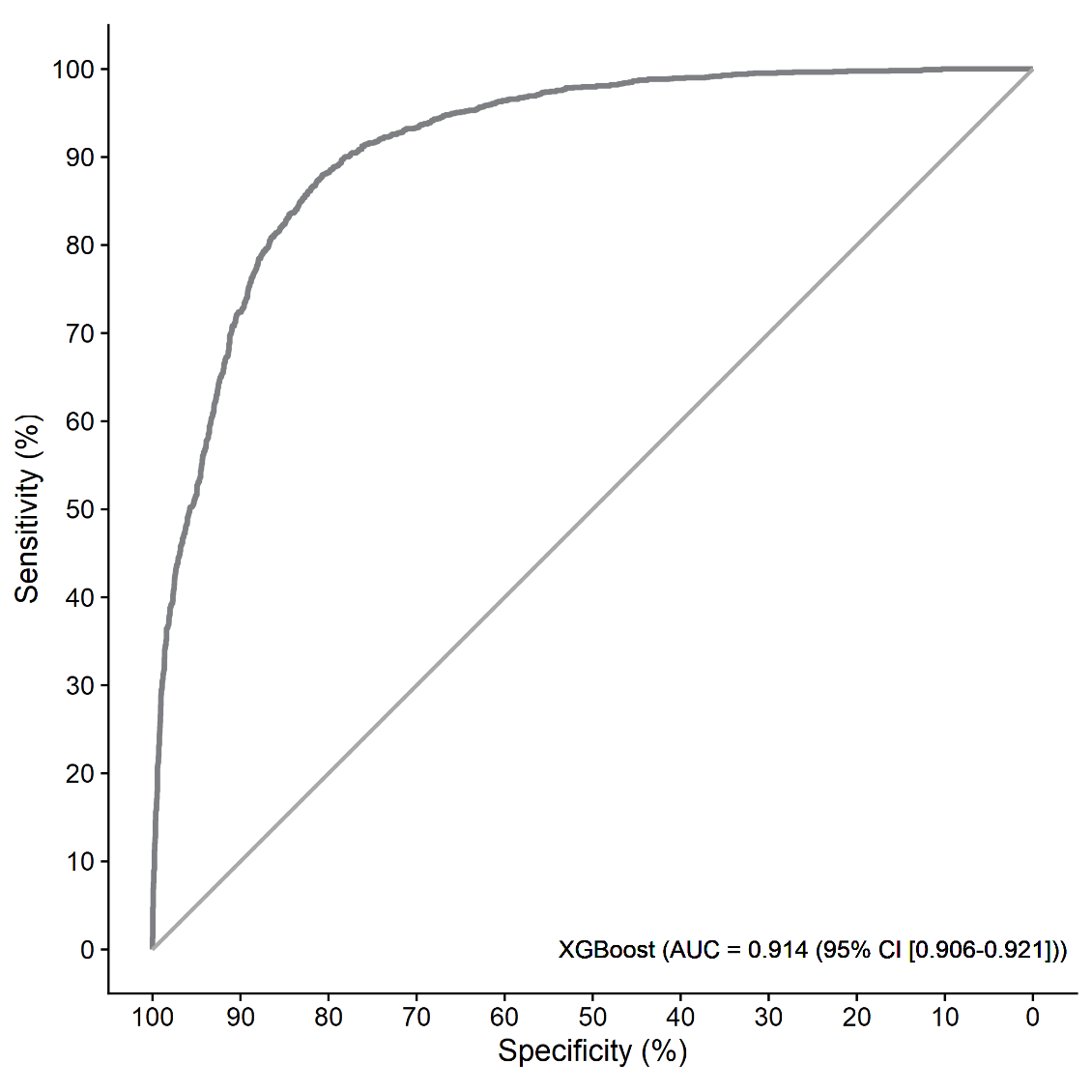
**a)** without prior heart failure for the CoDE-HF score (area under curve 0.914 [0.906-0.921])

**b)** with prior heart failure for the CoDE-HF score (area under curve 0.848 [0.831-0.864])

**a) b)**

**Supplementary Figure 5. Discrimination of the CoDE-HF score with MR-proANP. Receiver operator curve in patients**

**a)** without prior heart failure for the CoDE-HF score (area under curve 0.929 [0.919-0.939])

**b)** with prior heart failure for the CoDE-HF score (area under curve 0.857 [0.831-0.882])


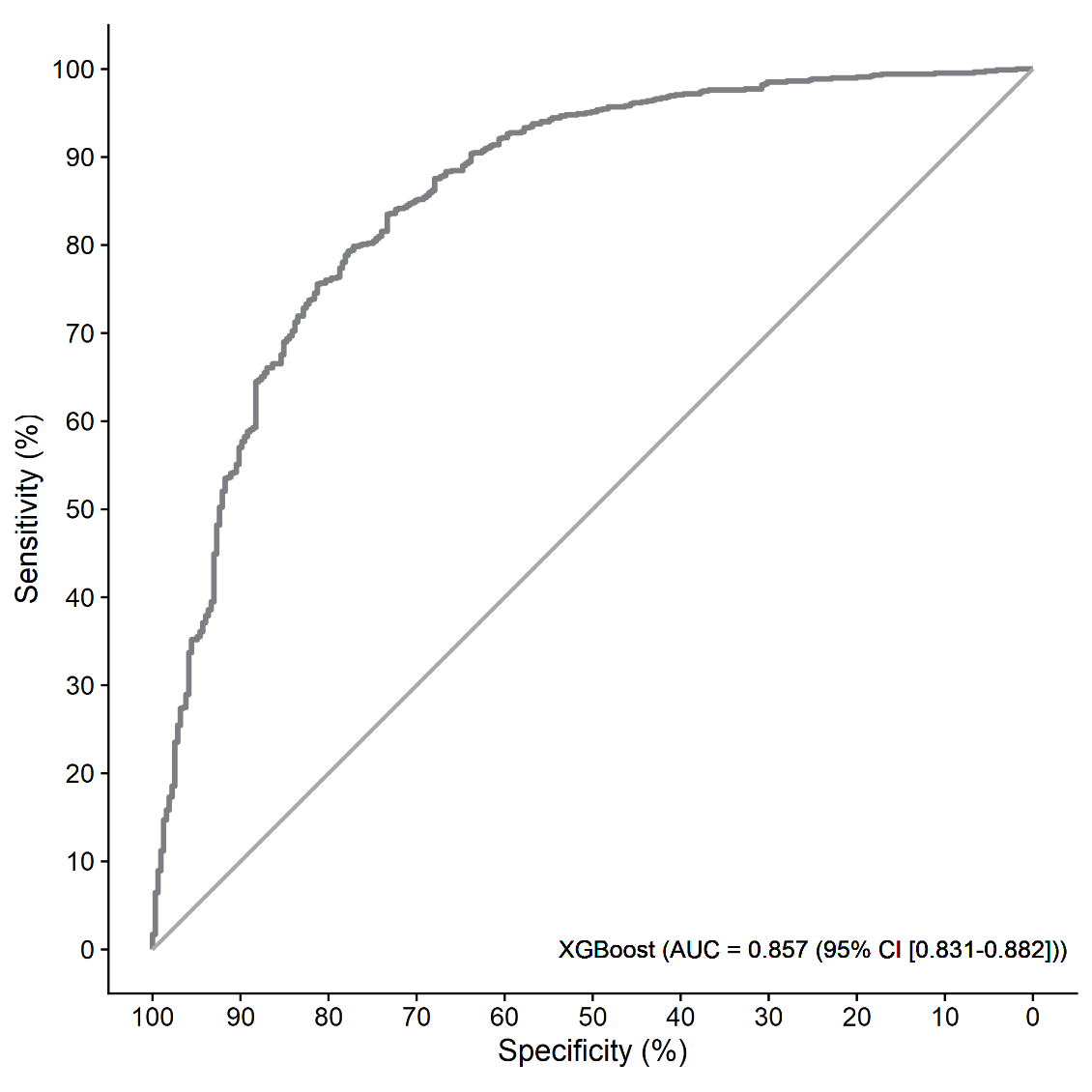

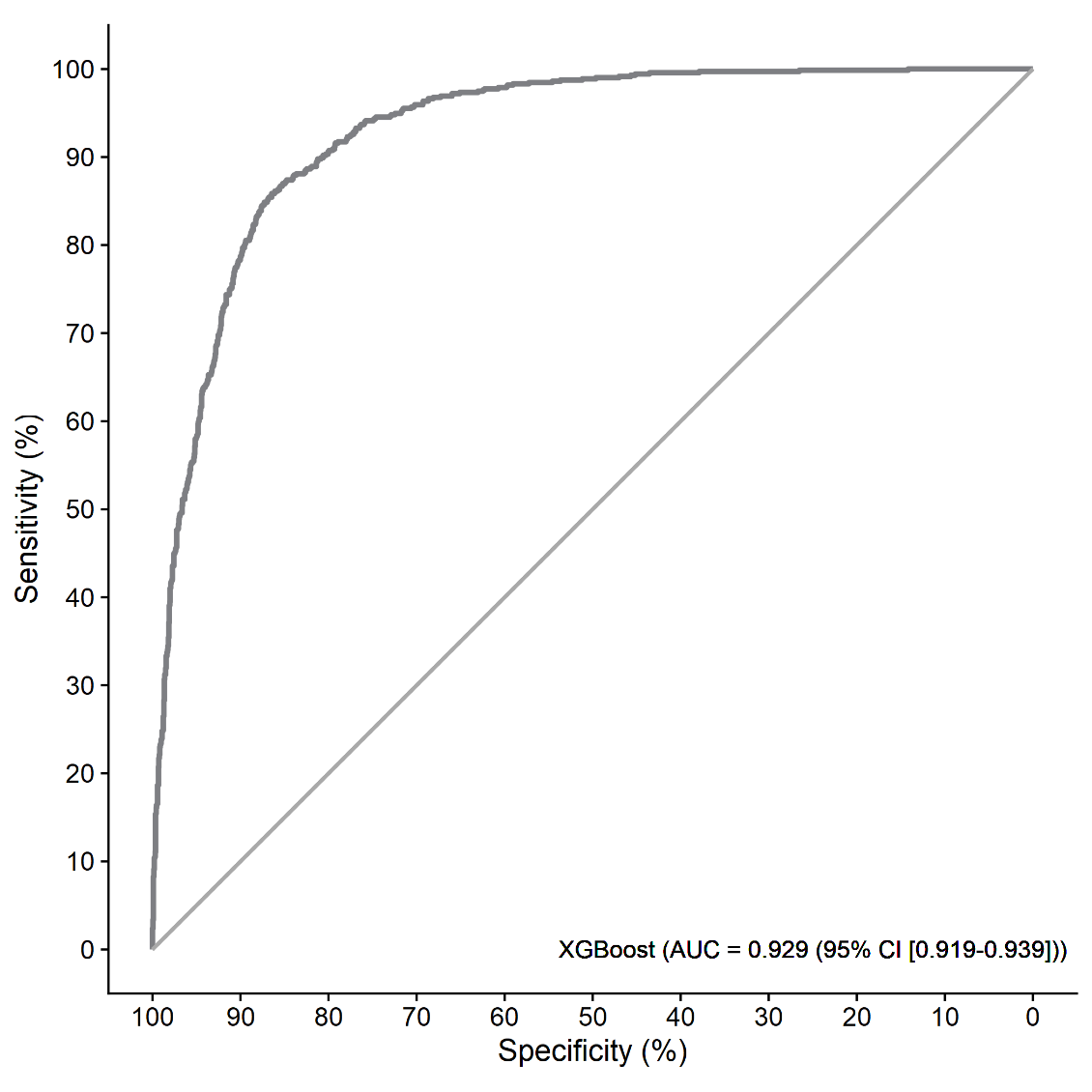
**a) b)**

**Supplementary Figure 6. Calibration plot of CoDE-HF score with MR-proANP in patients with**

**(a)** without prior heart failure for the CoDE-HF score

**(b)** with prior heart failure for the CoDE-HF score


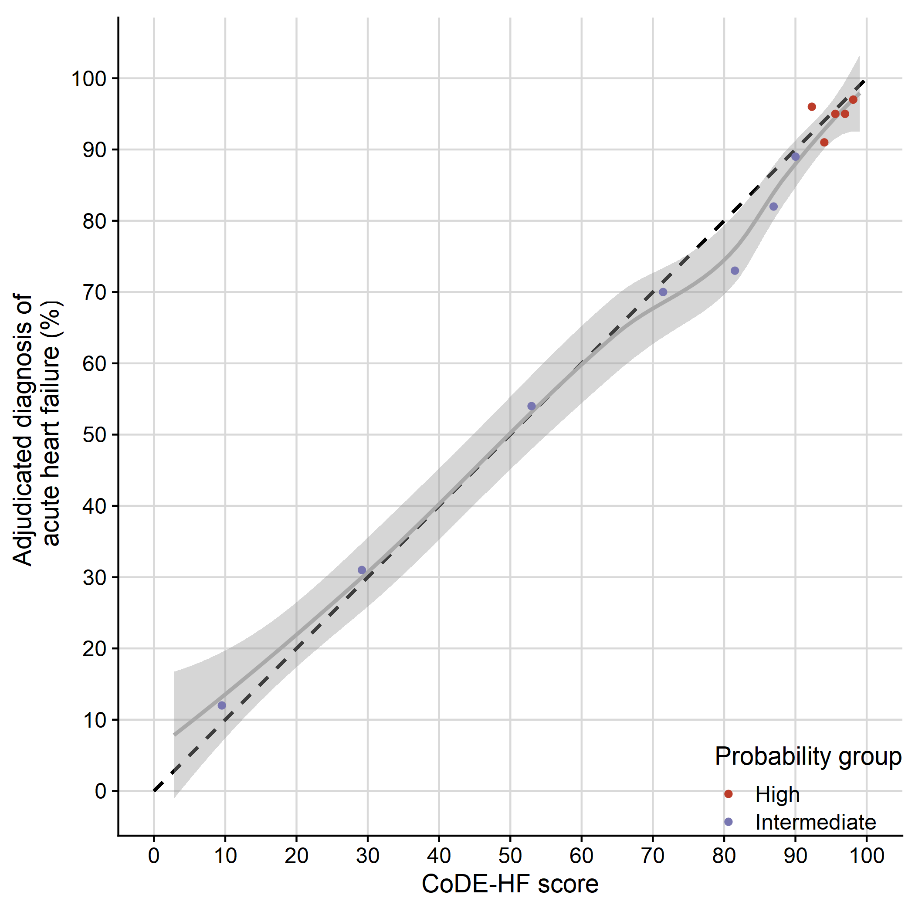

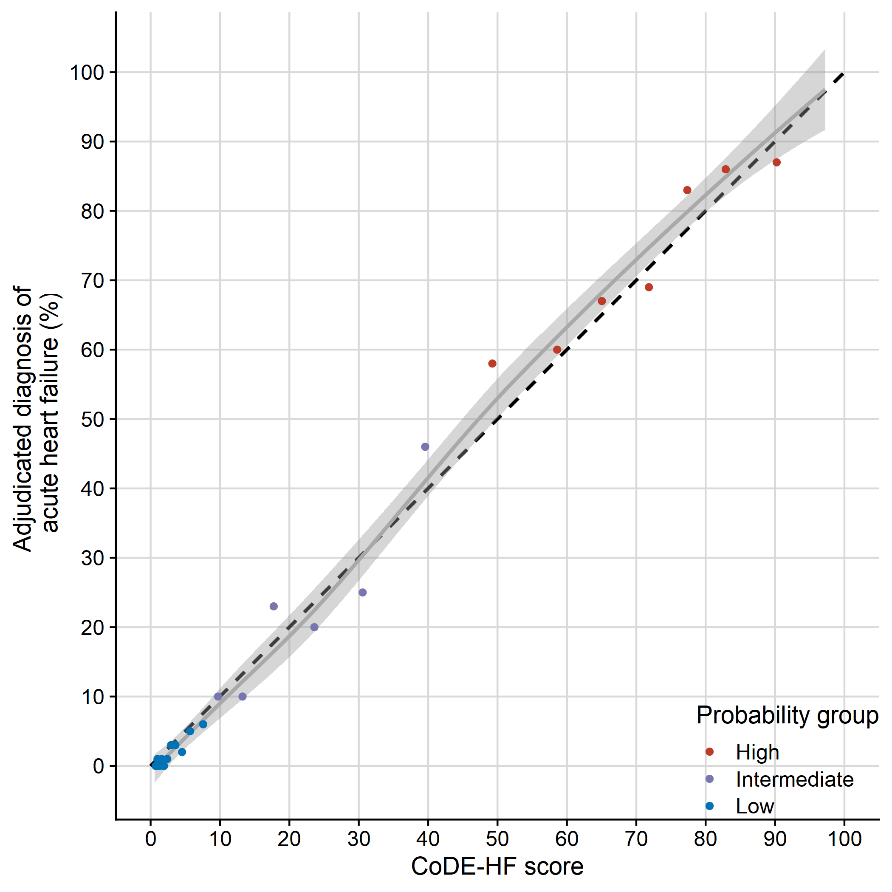
**a) b)**

**Supplementary Figure 7. Diagnostic performance of the CoDE-HF score with MR-proANP across patient subgroups.**

**(a)** Negative predictive value of the CoDE-HF rule-out score of 8.1 in patients without prior heart failure across patient subgroups.

**(b)** Positive predictive value of the CoDE-HF rule-in score of 46.0 in patients without prior heart failure across patient subgroups.

**(c)** Positive predictive value of the CoDE-HF rule-in score of 91.7 in patients with prior heart failure across patient subgroups.

**a)**

**
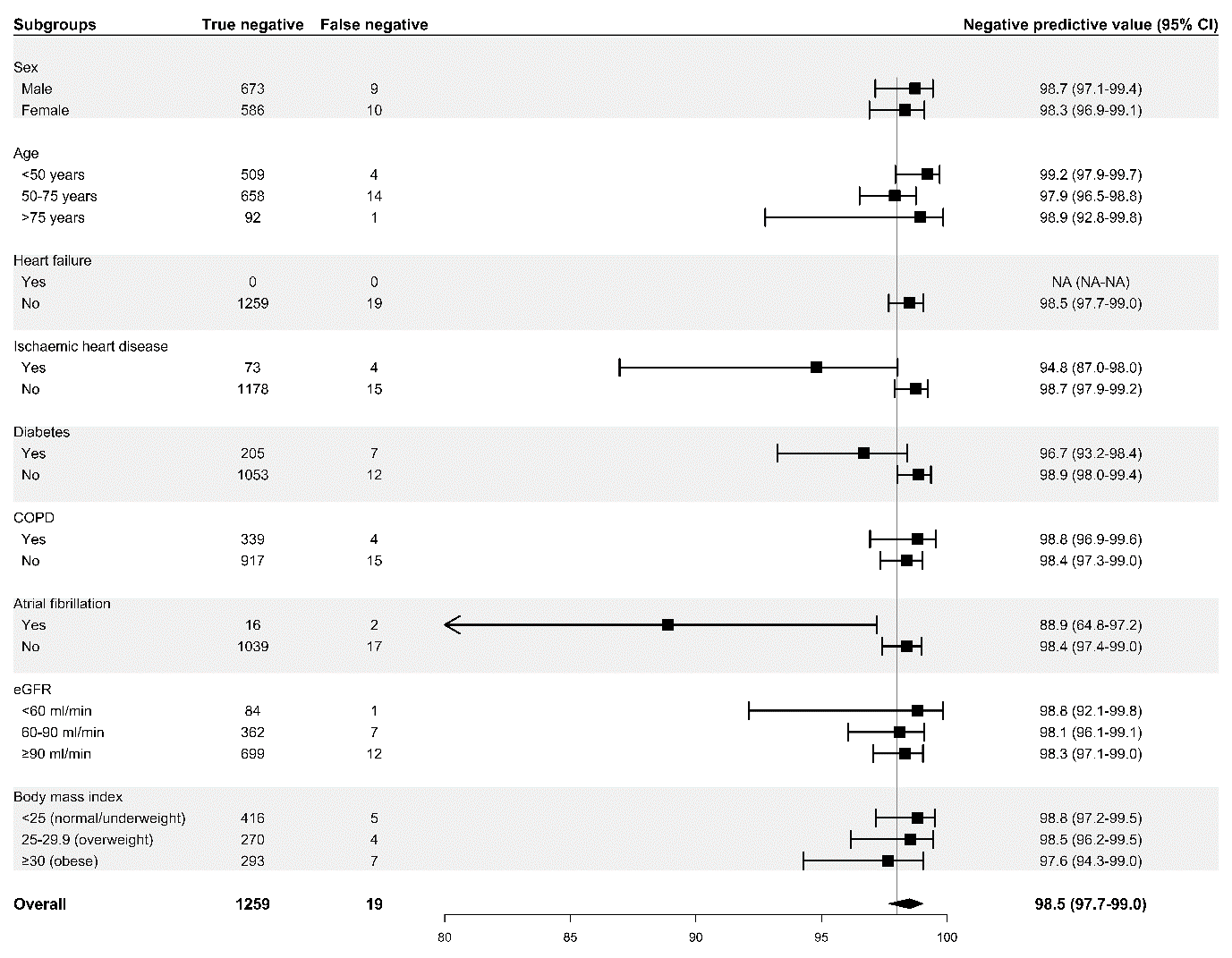
**

**b)**


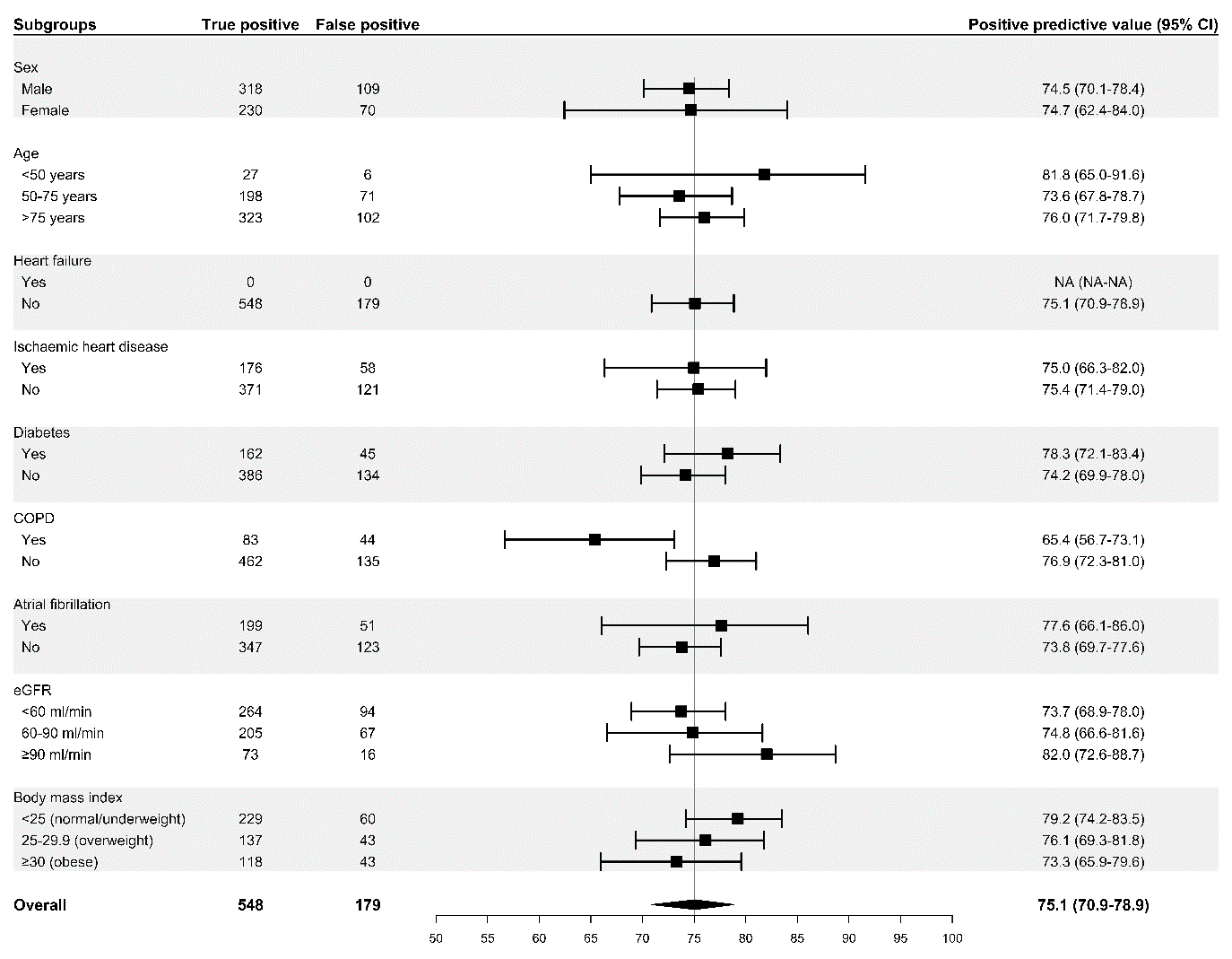


**c)**


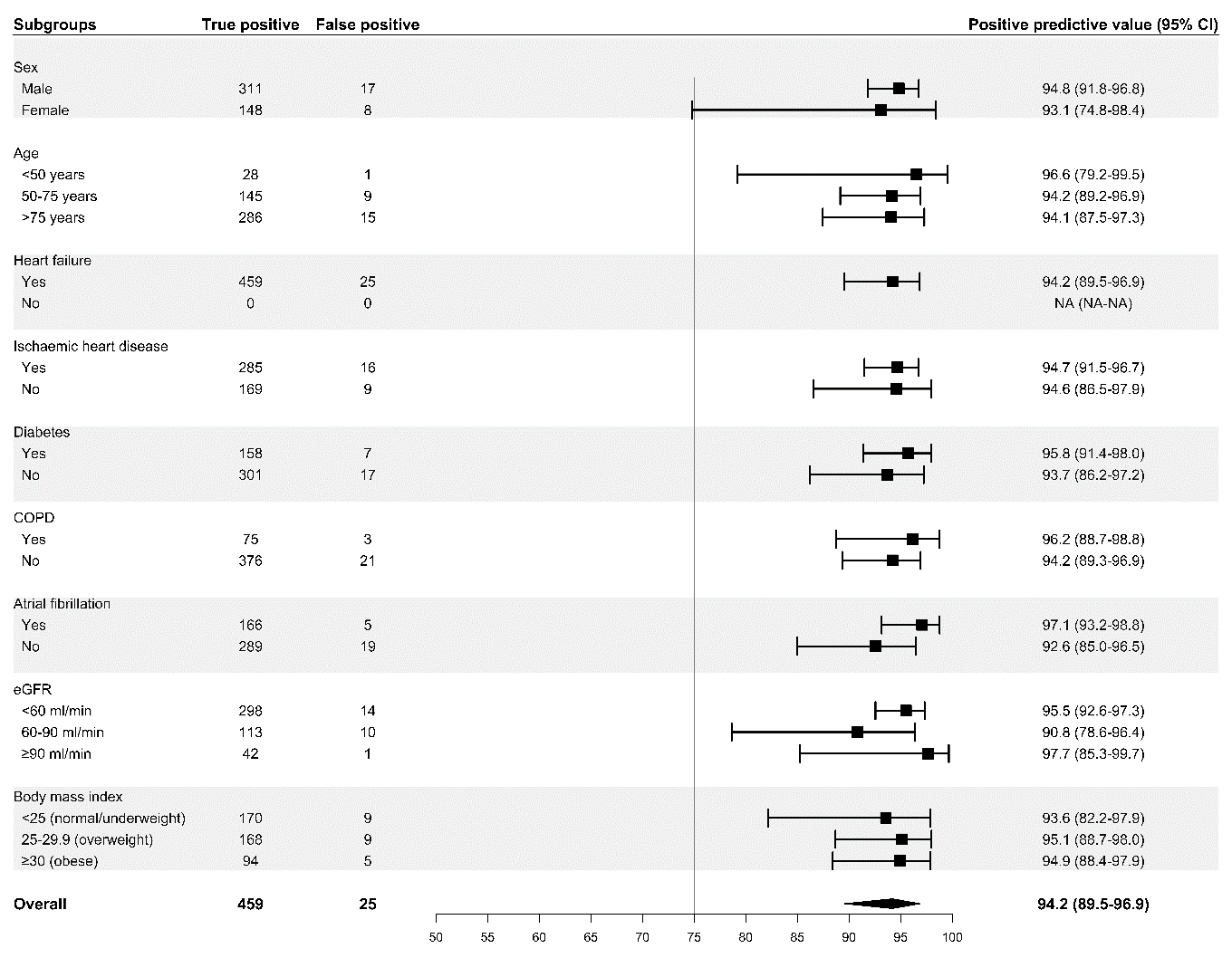


**Supplementary Figure 8. Decision curve analysis for CoDE-HF versus a) BNP alone and b) MR-proANP alone.**

1.
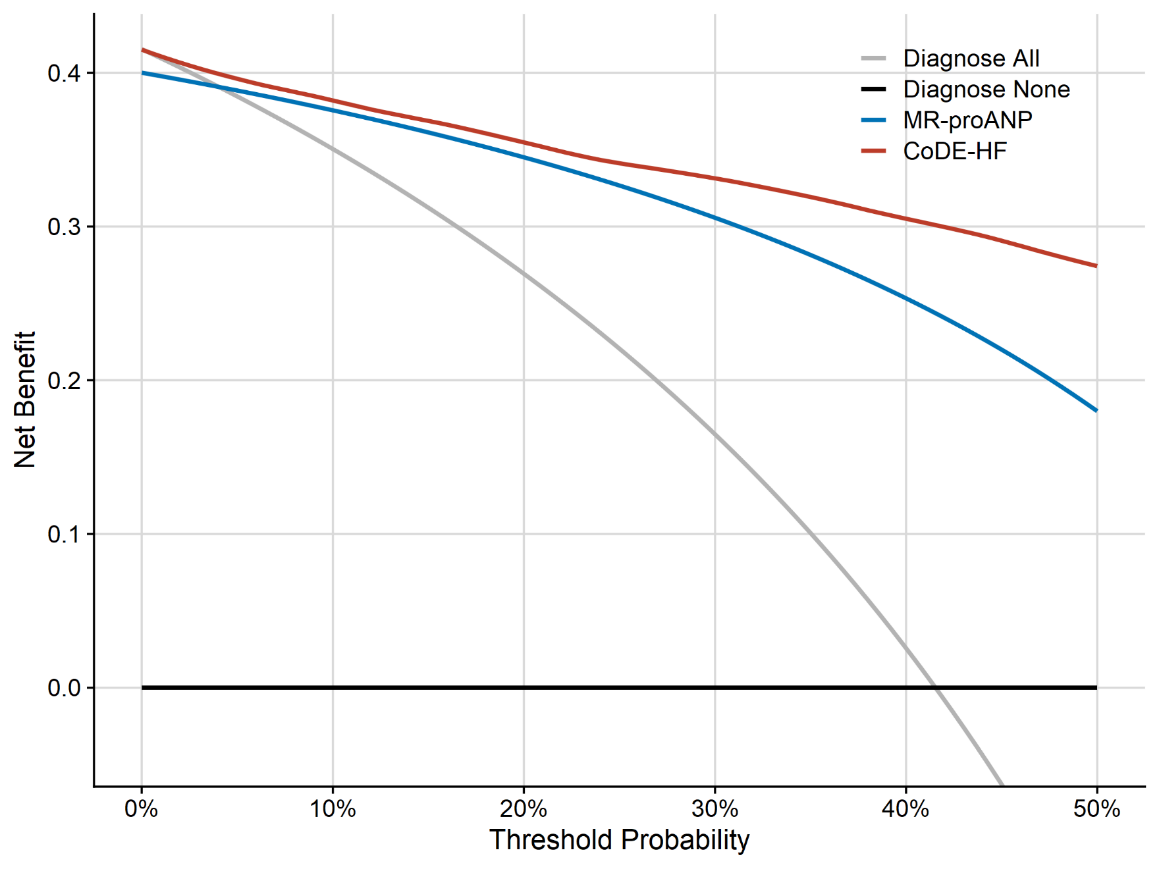

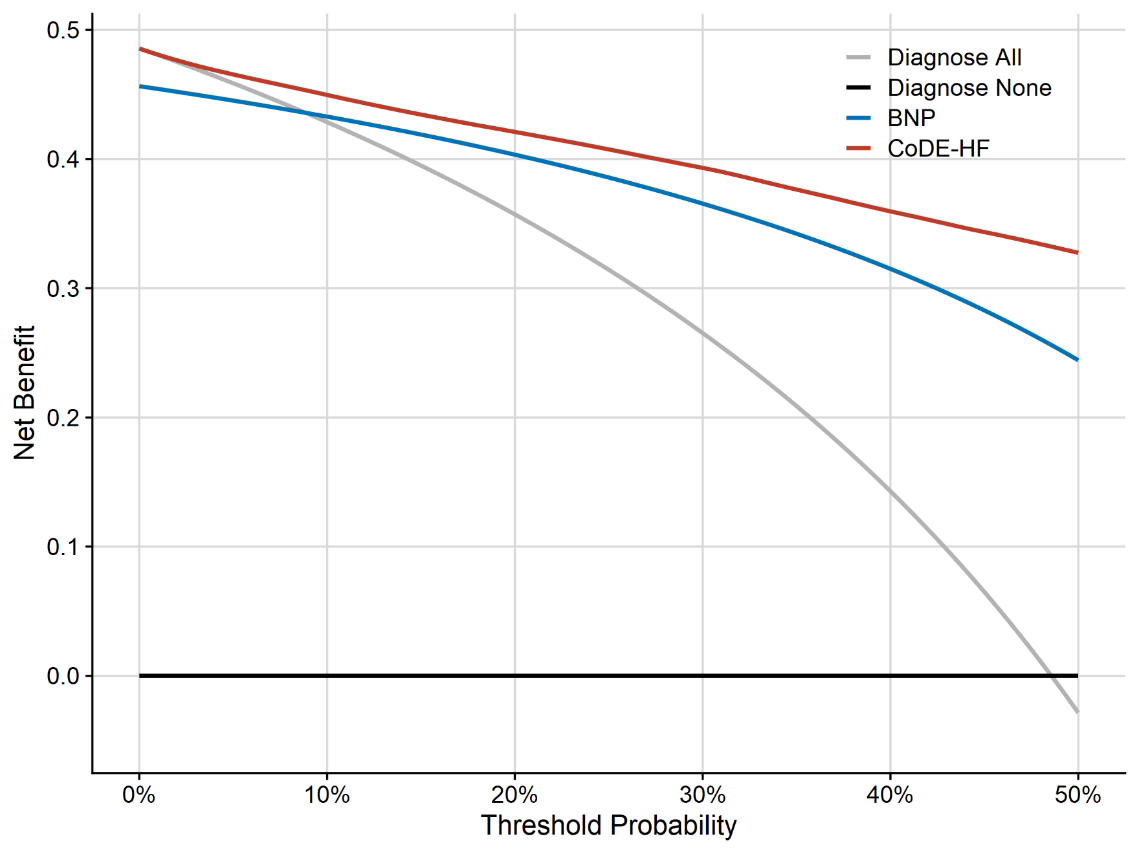
**b)**

The decision curve analysis presents the net benefit of the CoDE-HF score and NT-proBNP alone in comparison to hypothetical default approaches to diagnose all patients or no patients with acute heart failure. Net benefit for each approach is calculated across a range of possible threshold probabilities. Threshold probability is defined in this context as the minimum probability at which a diagnosis and treatment for acute heart failure is likely to be beneficial for patients. Net benefit is calculated using the following formula:

$Net benefit =sensitivity \times prevalence-\left( 1-specificity \right)\times\left( 1-prevalence \right)\times w$

*w* is the odds at the threshold probability.

**Supplementary Figure 9. Internal-external cross-validation of CoDE-HF with BNP.**

**a)** Patients without a prior history of heart failure across studies.

**
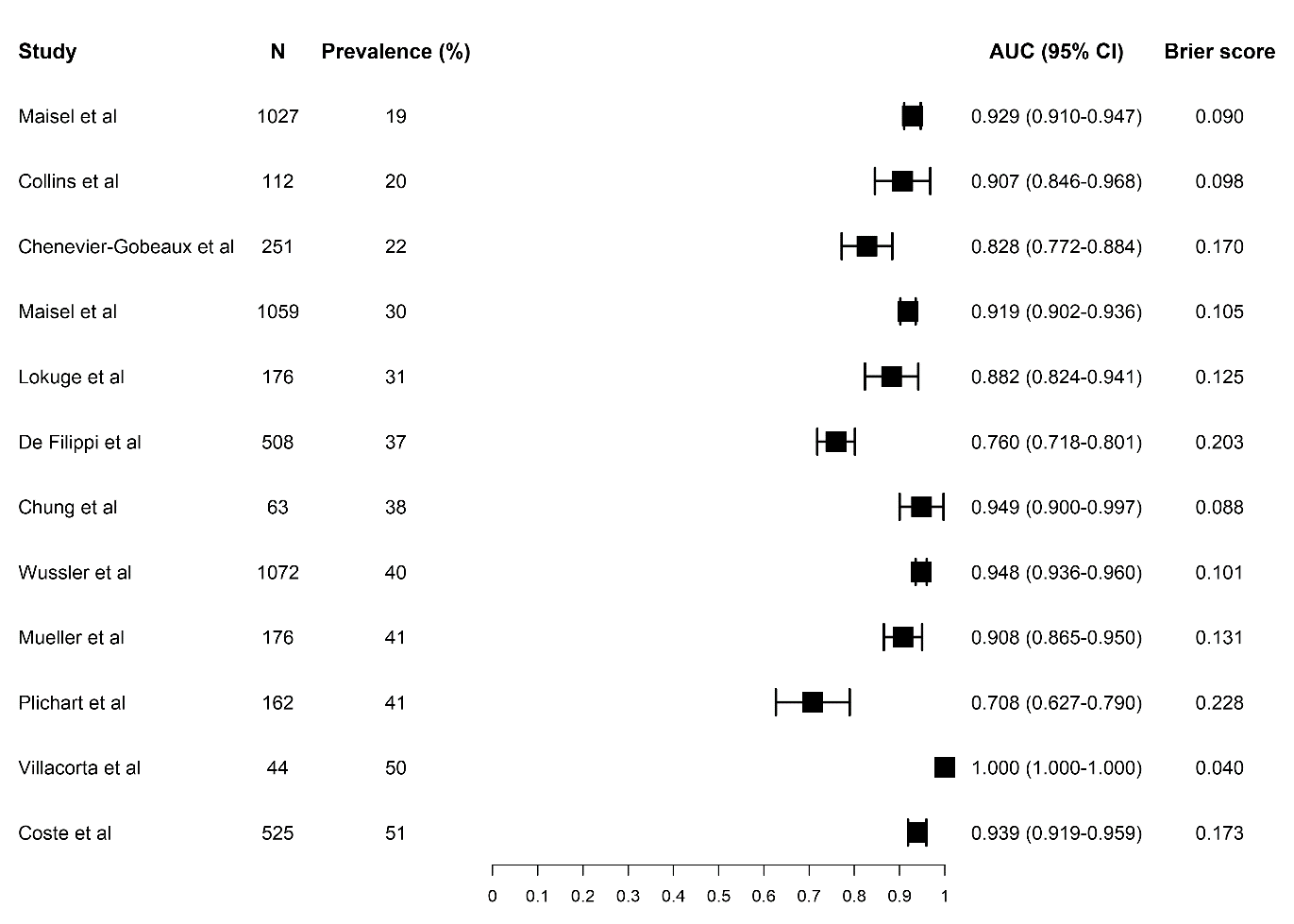
**

**b)** Patients with prior history of heart failure across studies**.**

**
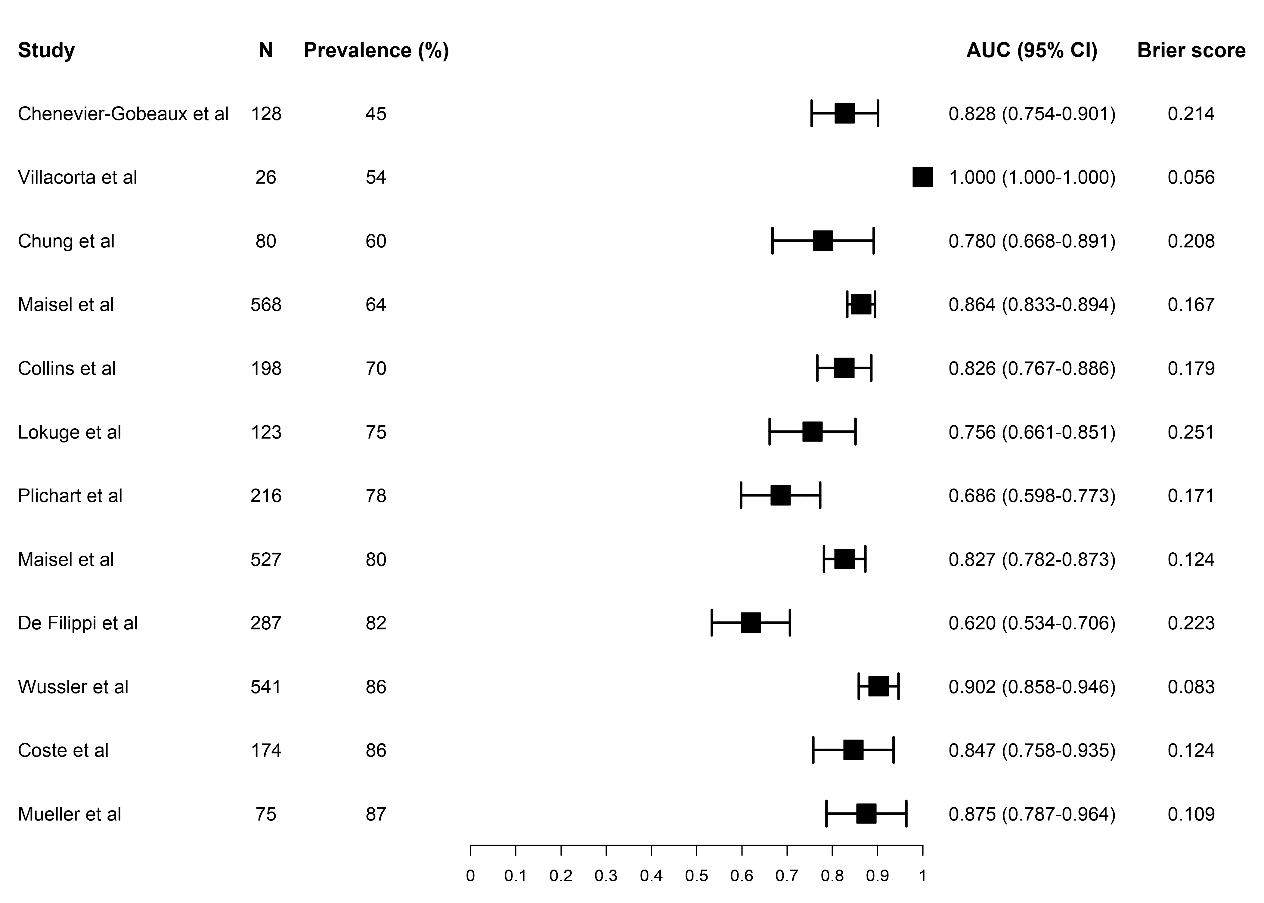
**

**Supplementary Figure 10. Internal-external cross-validation of CoDE-HF with MR-proANP.**

**a)** Patients without a prior history of heart failure across studies.

**
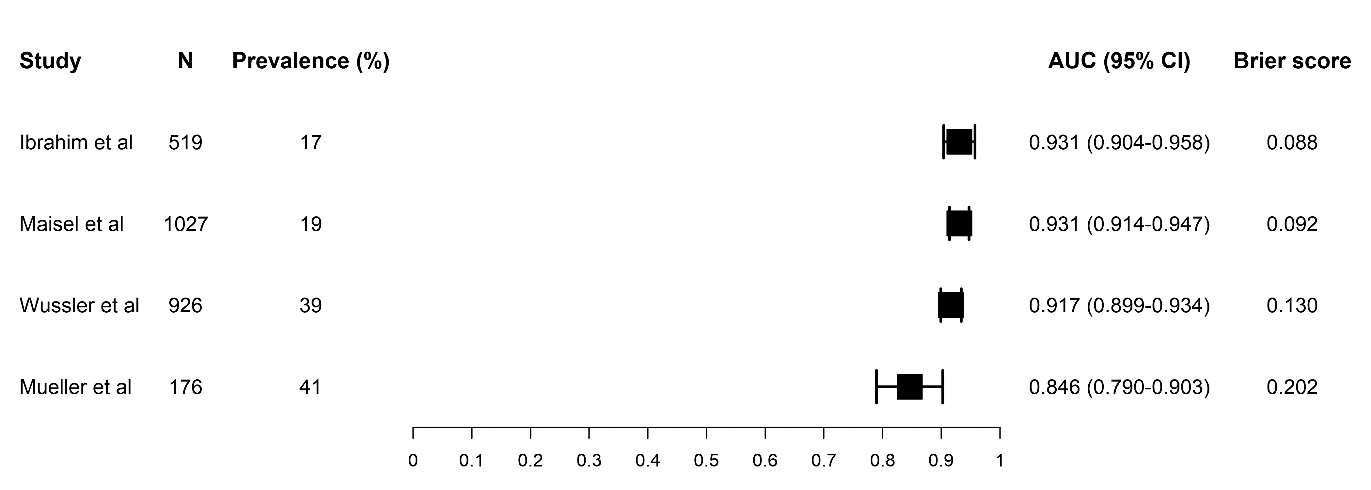
**

**b)** Patients with prior history of heart failure across studies.

**
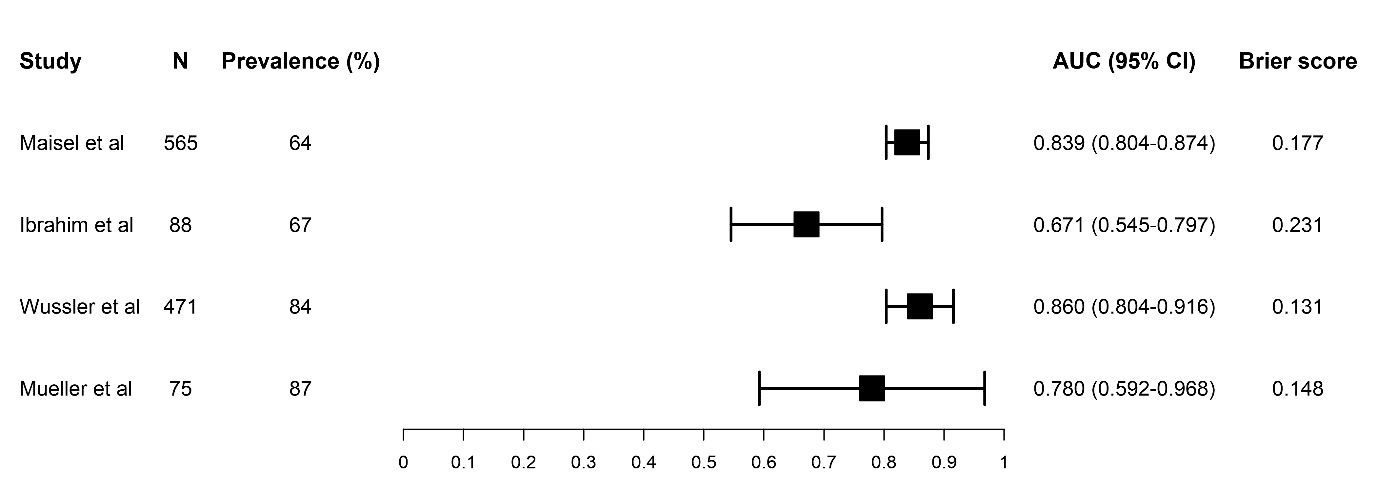
**

**Supplementary Figure 11.** Cumulative incidence of all-cause mortality stratified by CoDE-HF probability group for (a) using the BNP

and (b) Using the MR-proANP.

**a) b)**


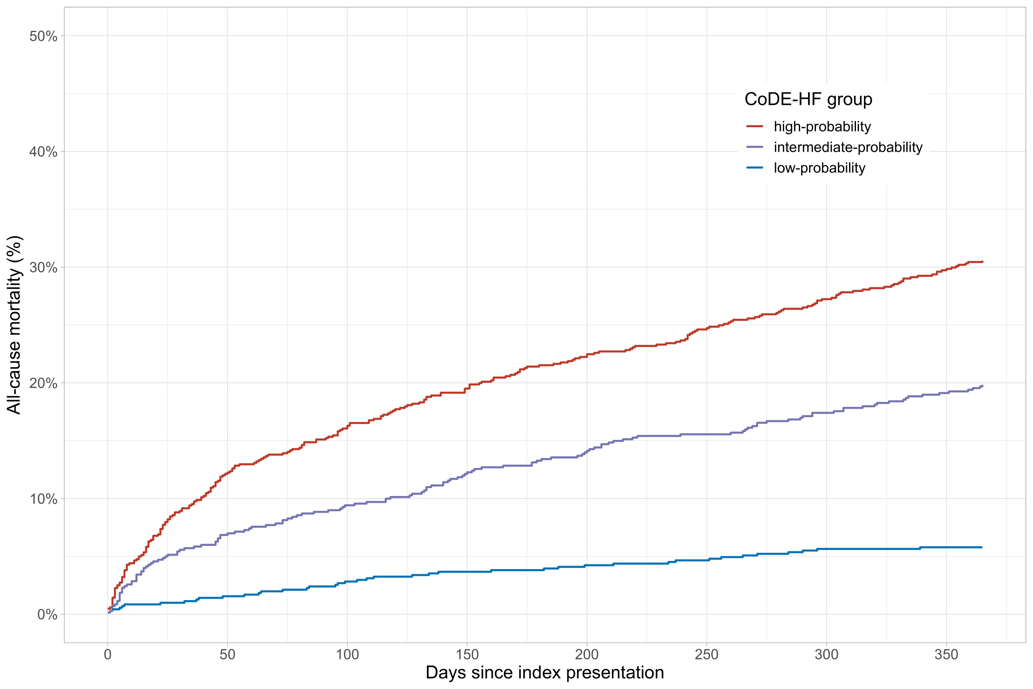

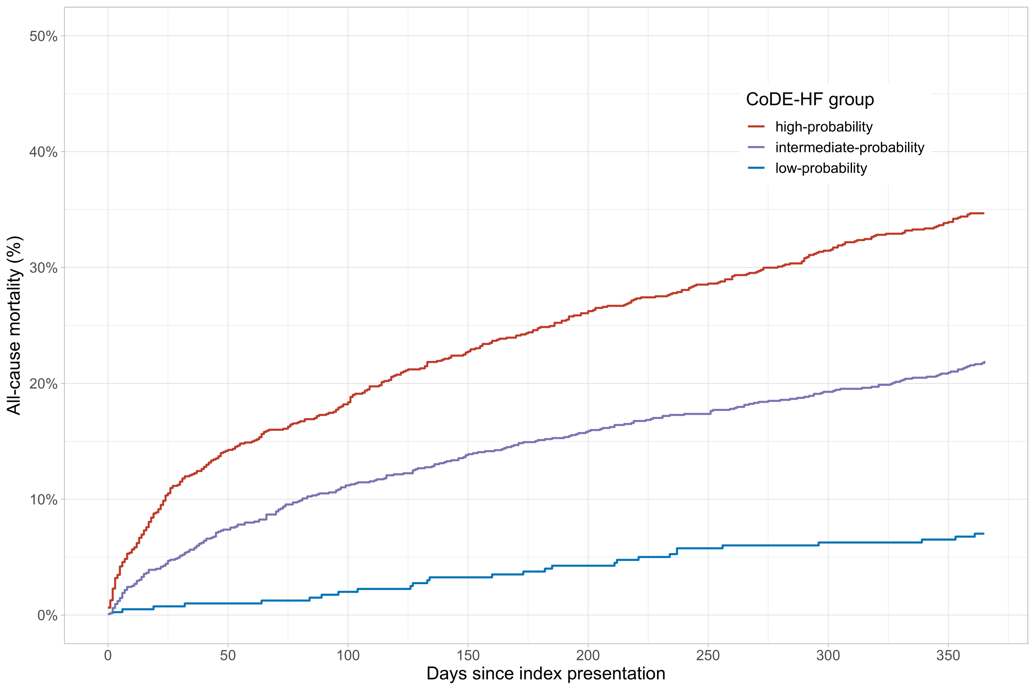


# **V. Checklists**

## **PRISMA-IPD Checklist of items to include when reporting a systematic review and meta-analysis of individual participant data (IPD)**

| **PRISMA-IPD**  **Section/topic** | **Item No** | **Checklist item** | **Reported on page** |
| --- | --- | --- | --- |
| **Title** | | | |
| Title | 1 | Identify the report as a systematic review and meta-analysis of individual participant data. | - |
| **Abstract** | | | |
| Structured summary | 2 | Provide a structured summary including as applicable: | 2 |
|  |  | **Background**: state research question and main objectives, with information on participants, interventions, comparators and outcomes. |  |
|  |  | **Methods**: report eligibility criteria; data sources including dates of last bibliographic search or elicitation, noting that IPD were sought; methods of assessing risk of bias. |  |
|  |  | **Results**: provide number and type of studies and participants identified and number (%) obtained; summary effect estimates for main outcomes (benefits and harms) with confidence intervals and measures of statistical heterogeneity. Describe the direction and size of summary effects in terms meaningful to those who would put findings into practice. |  |
|  |  | **Discussion:** state main strengths and limitations of the evidence, general interpretation of the results and any important implications. |  |
|  |  | **Other:** report primary funding source, registration number and registry name for the systematic review and IPD meta-analysis. |  |
| **Introduction** | | | |
| Rationale | 3 | Describe the rationale for the review in the context of what is already known. | 4 |
| Objectives | 4 | Provide an explicit statement of the questions being addressed with reference, as applicable, to participants, interventions, comparisons, outcomes and study design (PICOS). Include any hypotheses that relate to particular types of participant-level subgroups. | 5 |
| **Methods** | | | |
| Protocol and registration | 5 | Indicate if a protocol exists and where it can be accessed. If available, provide registration information including registration number and registry name. Provide publication details, if applicable. | 6 |
| Eligibility criteria | 6 | Specify inclusion and exclusion criteria including those relating to participants, interventions, comparisons, outcomes, study design and characteristics (e.g. years when conducted, required minimum follow-up). Note whether these were applied at the study or individual level i.e. whether eligible participants were included (and ineligible participants excluded) from a study that included a wider population than specified by the review inclusion criteria. The rationale for criteria should be stated. | 6 |
| Identifying studies - information sources | 7 | Describe all methods of identifying published and unpublished studies including, as applicable: which bibliographic databases were searched with dates of coverage; details of any hand searching including of conference proceedings; use of study registers and agency or company databases; contact with the original research team and experts in the field; open adverts and surveys. Give the date of last search or elicitation. | 6 |
| Identifying studies - search | 8 | Present the full electronic search strategy for at least one database, including any limits used, such that it could be repeated. | Supplement, page 6 |
| Study selection processes | 9 | State the process for determining which studies were eligible for inclusion. | 6 |
| Data collection processes | 10 | Describe how IPD were requested, collected and managed, including any processes for querying and confirming data with investigators. If IPD were not sought from any eligible study, the reason for this should be stated (for each such study). | 6 |
|  |  | If applicable, describe how any studies for which IPD were not available were dealt with. This should include whether, how and what aggregate data were sought or extracted from study reports and publications (such as extracting data independently in duplicate) and any processes for obtaining and confirming these data with investigators. |  |
| Data items | 11 | Describe how the information and variables to be collected were chosen. List and define all study level and participant level data that were sought, including baseline and follow-up information. If applicable, describe methods of standardising or translating variables within the IPD datasets to ensure common scales or measurements across studies. | 6 |
| IPD integrity | A1 | Describe what aspects of IPD were subject to data checking (such as sequence generation, data consistency and completeness, baseline imbalance) and how this was done. | 6 |
| Risk of bias assessment in individual studies. | 12 | Describe methods used to assess risk of bias in the individual studies and whether this was applied separately for each outcome. If applicable, describe how findings of IPD checking were used to inform the assessment. Report if and how risk of bias assessment was used in any data synthesis. | 6-7 |
| Specification of outcomes and effect measures | 13 | State all treatment comparisons of interests. State all outcomes addressed and define them in detail. State whether they were pre-specified for the review and, if applicable, whether they were primary/main or secondary/additional outcomes. Give the principal measures of effect (such as risk ratio, hazard ratio, difference in means) used for each outcome. | 7 |
| Synthesis methods | 14 | Describe the meta-analysis methods used to synthesise IPD. Specify any statistical methods and models used. Issues should include (but are not restricted to):   - Use of a one-stage or two-stage approach. - How effect estimates were generated separately within each study and combined across studies (where applicable). - Specification of one-stage models (where applicable) including how clustering of patients within studies was accounted for. - Use of fixed or random effects models and any other model assumptions, such as proportional hazards. - How (summary) survival curves were generated (where applicable). - Methods for quantifying statistical heterogeneity (such as I^2^ and τ^2^). - How studies providing IPD and not providing IPD were analysed together (where applicable). - How missing data within the IPD were dealt with (where applicable). | 7 |
| Exploration of variation in effects | A2 | If applicable, describe any methods used to explore variation in effects by study or participant level characteristics (such as estimation of interactions between effect and covariates). State all participant-level characteristics that were analysed as potential effect modifiers, and whether these were pre-specified. | 7 |
| Risk of bias across studies | 15 | Specify any assessment of risk of bias relating to the accumulated body of evidence, including any pertaining to not obtaining IPD for particular studies, outcomes or other variables. | 6-7 |
| Additional analyses | 16 | Describe methods of any additional analyses, including sensitivity analyses. State which of these were pre-specified. | 7-9 |
| **Results** | | | |
| Study selection and IPD obtained | 17 | Give numbers of studies screened, assessed for eligibility, and included in the systematic review with reasons for exclusions at each stage. Indicate the number of studies and participants for which IPD were sought and for which IPD were obtained. For those studies where IPD were not available, give the numbers of studies and participants for which aggregate data were available. Report reasons for non-availability of IPD. Include a flow diagram. | Supplement page 33 |
| Study characteristics | 18 | For each study, present information on key study and participant characteristics (such as description of interventions, numbers of participants, demographic data, unavailability of outcomes, funding source, and if applicable duration of follow-up). Provide (main) citations for each study. Where applicable, also report similar study characteristics for any studies not providing IPD. | Supplement page 18-22 |
| IPD integrity | A3 | Report any important issues identified in checking IPD or state that there were none. | 11 and Supplement,  page 32 |
| Risk of bias within studies | 19 | Present data on risk of bias assessments. If applicable, describe whether data checking led to the up-weighting or down-weighting of these assessments. Consider how any potential bias impacts on the robustness of meta-analysis conclusions. | 11 |
| Results of individual studies | 20 | For each comparison and for each main outcome (benefit or harm), for each individual study report the number of eligible participants for which data were obtained and show simple summary data for each intervention group (including, where applicable, the number of events), effect estimates and confidence intervals. These may be tabulated or included on a forest plot. | Supplement,page 34-35, 46-47 |
| Results of syntheses | 21 | Present summary effects for each meta-analysis undertaken, including confidence intervals and measures of statistical heterogeneity. State whether the analysis was pre-specified, and report the numbers of studies and participants and, where applicable, the number of events on which it is based. | 10-11 |
|  |  | When exploring variation in effects due to patient or study characteristics, present summary interaction estimates for each characteristic examined, including confidence intervals and measures of statistical heterogeneity. State whether the analysis was pre-specified. State whether any interaction is consistent across trials. |  |
|  |  | Provide a description of the direction and size of effect in terms meaningful to those who would put findings into practice. |  |
| Risk of bias across studies | 22 | Present results of any assessment of risk of bias relating to the accumulated body of evidence, including any pertaining to the availability and representativeness of available studies, outcomes or other variables. | Supplement, page 21 |
| Additional analyses | 23 | Give results of any additional analyses (e.g. sensitivity analyses). If applicable, this should also include any analyses that incorporate aggregate data for studies that do not have IPD. If applicable, summarise the main meta-analysis results following the inclusion or exclusion of studies for which IPD were not available. | 11-13 |
| **Discussion** | | | |
| Summary of evidence | 24 | Summarise the main findings, including the strength of evidence for each main outcome. | 14 |
| Strengths and limitations | 25 | Discuss any important strengths and limitations of the evidence including the benefits of access to IPD and any limitations arising from IPD that were not available. | 14 & 17 |
| Conclusions | 26 | Provide a general interpretation of the findings in the context of other evidence. | 18 |
| Implications | A4 | Consider relevance to key groups (such as policy makers, service providers and service users). Consider implications for future research. | 16 |
| **Funding** | | | |
| Funding | 27 | Describe sources of funding and other support (such as supply of IPD), and the role in the systematic review of those providing such support. | 20 |

**A1 – A3 denote new items that are additional to standard PRISMA items. A4 has been created as a result of re-arranging content of the standard PRISMA statement to suit the way that systematic review IPD meta-analyses are reported.**

© Reproduced with permission of the PRISMA IPD Group, which encourages sharing and reuse for non-commercial purpose

## **TRIPOD Checklist: Prediction Model Development and Validation**

| Section/Topic | Item |  | Checklist Item | Page |
| --- | --- | --- | --- | --- |
| Title and abstract | | | | |
| Title | 1 | D;V | Identify the study as developing and/or validating a multivariable prediction model, the target population, and the outcome to be predicted. | 1 |
| Abstract | 2 | D;V | Provide a summary of objectives, study design, setting, participants, sample size, predictors, outcome, statistical analysis, results, and conclusions. | 2-3 |
| Introduction | | | | |
| Background and objectives | 3a | D;V | Explain the medical context (including whether diagnostic or prognostic) and rationale for developing or validating the multivariable prediction model, including references to existing models. | 4-5 |
|  | 3b | D;V | Specify the objectives, including whether the study describes the development or validation of the model or both. | 5 |
| Methods | | | | |
| Source of data | 4a | D;V | Describe the study design or source of data (e.g., randomized trial, cohort, or registry data), separately for the development and validation data sets, if applicable. | 6 |
|  | 4b | D;V | Specify the key study dates, including start of accrual; end of accrual; and, if applicable, end of follow-up. | 6 |
| Participants | 5a | D;V | Specify key elements of the study setting (e.g., primary care, secondary care, general population) including number and location of centres. | 6 |
|  | 5b | D;V | Describe eligibility criteria for participants. | 6 |
|  | 5c | D;V | Give details of treatments received, if relevant. | NA |
| Outcome | 6a | D;V | Clearly define the outcome that is predicted by the prediction model, including how and when assessed. | 6 |
|  | 6b | D;V | Report any actions to blind assessment of the outcome to be predicted. | 6 |
| Predictors | 7a | D;V | Clearly define all predictors used in developing or validating the multivariable prediction model, including how and when they were measured. | 8 |
|  | 7b | D;V | Report any actions to blind assessment of predictors for the outcome and other predictors. | NA |
| Sample size | 8 | D;V | Explain how the study size was arrived at. | 7-8 |
| Missing data | 9 | D;V | Describe how missing data were handled (e.g., complete-case analysis, single imputation, multiple imputation) with details of any imputation method. | 8 |
| Statistical analysis methods | 10a | D | Describe how predictors were handled in the analyses. | 8 |
|  | 10b | D | Specify type of model, all model-building procedures (including any predictor selection), and method for internal validation. | 8 |
|  | 10c | V | For validation, describe how the predictions were calculated. | 8 |
|  | 10d | D;V | Specify all measures used to assess model performance and, if relevant, to compare multiple models. | 9 |
|  | 10e | V | Describe any model updating (e.g., recalibration) arising from the validation, if done. | NA |
| Risk groups | 11 | D;V | Provide details on how risk groups were created, if done. | 9 |
| Development vs. validation | 12 | V | For validation, identify any differences from the development data in setting, eligibility criteria, outcome, and predictors. | 7-8 |
| Results | | | | |
| Participants | 13a | D;V | Describe the flow of participants through the study, including the number of participants with and without the outcome and, if applicable, a summary of the follow-up time. A diagram may be helpful. | 10 |
|  | 13b | D;V | Describe the characteristics of the participants (basic demographics, clinical features, available predictors), including the number of participants with missing data for predictors and outcome. | 10 |
|  | 13c | V | For validation, show a comparison with the development data of the distribution of important variables (demographics, predictors and outcome). | 10 |
| Model development | 14a | D | Specify the number of participants and outcome events in each analysis. | 10 |
|  | 14b | D | If done, report the unadjusted association between each candidate predictor and outcome. | 10 |
| Model specification | 15a | D | Present the full prediction model to allow predictions for individuals (i.e., all regression coefficients, and model intercept or baseline survival at a given time point). | NA |
|  | 15b | D | Explain how to the use the prediction model. | 11 |
| Model performance | 16 | D;V | Report performance measures (with CIs) for the prediction model. | 11-12 |
| Model-updating | 17 | V | If done, report the results from any model updating (i.e., model specification, model performance). | NA |
| Discussion | | | | |
| Limitations | 18 | D;V | Discuss any limitations of the study (such as nonrepresentative sample, few events per predictor, missing data). | 17 |
| Interpretation | 19a | V | For validation, discuss the results with reference to performance in the development data, and any other validation data. | 16 |
|  | 19b | D;V | Give an overall interpretation of the results, considering objectives, limitations, results from similar studies, and other relevant evidence. | 14 |
| Implications | 20 | D;V | Discuss the potential clinical use of the model and implications for future research. | 16 |
| Other information | | | | |
| Supplementary information | 21 | D;V | Provide information about the availability of supplementary resources, such as study protocol, Web calculator, and data sets. | 7 |
| Funding | 22 | D;V | Give the source of funding and the role of the funders for the present study. | 20 |

*Items relevant only to the development of a prediction model are denoted by D, items relating solely to a validation of a prediction model are denoted by V, and items relating to both are denoted D;V. We recommend using the TRIPOD Checklist in conjunction with the TRIPOD Explanation and Elaboration document.

# **References**

1. Chen T, Guestrin C. XGBoost: A Scalable Tree Boosting System. ArXiv e-prints 2016.

2. Friedman JH. Greedy Function Approximation: A Gradient Boosting Machine. The Annals of Statistics 2001;29:1189-1232.

3. Friedman J, Hastie T, Tibshirani R. Additive logistic regression: a statistical view of boosting. Ann Statist 2000;28:337-407.
